# Incidence, associated factors and impact of the post-COVID-19 condition in Brazil: Study protocol of an observational cohort during the Omicron phase

**DOI:** 10.1371/journal.pone.0322466

**Published:** 2025-04-24

**Authors:** Caroline Cabral Robinson, Daniel Sganzerla, Joselia Larger Manfio, Renata Kochhann, Adriana Raquel Binsfeld Hess, Patricia Ferreira da Silva, Nathan Iori Camargo, Andressa Fiorenzano Nunes, Nícolas Taciano Kunkel, Priscila Kieling Binsfeld, Tatiane Aparecida de Miranda, Bruna Machado Barroso, Thalia de Paula Morais, Diogo Rosas Ferreira, Sérgio Renato da Rosa Decker, Regis Goulart Rosa, Maicon Falavigna

**Affiliations:** 1 Inova Research - INOVA MEDICAL - HEALTH SERVICES AND MANAGEMENT LTD, Cachoeirinha, Rio Grande do Sul, Brazil; 2 Research Projects Office, Hospital Moinhos de Vento, Porto Alegre, Rio Grande do Sul, Brazil; 3 Hess Pesquisa e Desenvolvimento Ltda, Taquara, Rio Grande do Sul, Brazil; 4 Programa de Pós-graduação em Tecnologias da Informação e Gestão em Saúde, Universidade Federal de Ciências da Saúde de Porto Alegre, Porto Alegre, Rio Grande do Sul, Brazil; 5 Departamento de Medicina, Universidade Federal do Paraná, Maringá, Paraná, Brazil; 6 Programa de Pós-Graduação em Ciências Farmacêuticas, Universidade Estadual do Oeste do Paraná, Cascavel, Paraná, Brazil; 7 Faculdade SOGIPA, Porto Alegre, Rio Grande do Sul, Brazil; 8 Programa de Pós-graduação em Nutrição, Universidade Federal de Santa Catarina, Florianópolis, Santa Catarina, Brazil; 9 Othus Solutions, Porto Alegre, Rio Grande do Sul, Brazil; 10 Programa de Pós-graduação em Epidemiologia, Universidade Federal do Rio Grande do Sul, Porto Alegre, Rio Grande do Sul, Brazil; 11 Internal Medicine Department, Moinhos de Vento Hospital, Porto Alegre, Brazil; 12 Programa de Pós-graduação em Cardiologia e Ciências Cardiovasculares, Universidade Federal do Rio Grande do Sul, Porto Alegre, Brazil; 13 Faculdade de Medicina, Universidade do Vale do Rio dos Sinos, Porto Alegre, Brazil; 14 Departamento de Medicina Interna, Faculdade de Medicina, Universidade Federal do Rio Grande do Sul, Porto Alegre, Brazil; 15 National Institute of Science and Technology for Health Technology Assessment, Clinical Research Center, Hospital de Clínicas de Porto Alegre, Porto Alegre, Brazil; Federal University of Ceara, BRAZIL

## Abstract

The novel coronavirus (SARS-CoV-2) has caused a significant impact in Brazil, with over 37 million cases and 690,000 deaths. Survivors often experience prolonged symptoms, termed post-COVID-19 condition or long COVID, affecting various aspects of life. Data on post-COVID-19 in Brazil, particularly amid the Omicron variant, are limited. This study aims to address this gap by assessing the incidence, associated factors, and impact of post-COVID-19 in adults infected during the Omicron phase, using the World Health Organization case definition. This Brazilian cohort study involves virtual recruitment and a nested case-control design. Participants from all regions will be recruited through electronic announcements. Inclusion criteria comprise age ≥ 18, Brazilian residency, and confirmed symptomatic SARS-CoV-2 infection. A nested case-control study will compare cognitive domains in post-COVID-19 cognitive dysfunction cases and controls. Data collection will utilize standardized instruments, including the EQ-5D-3L, Lawton & Brody Scale, Barthel Index, and others. Statistical analyses will employ adjusted models for primary and secondary outcomes. The study follows ethical guidelines, with an informed consent process tailored to participant preferences. Confidentiality measures include restricted access and secure electronic data storage. Results will be disseminated in academic forums, peer-reviewed journals, and directly communicated to participants. Authorship criteria align with international standards, and data sharing requests will be evaluated by the steering committee. The study is currently recruiting participants.

## Introduction

The infection caused by the novel coronavirus (SARS-CoV-2), known as COVID-19, has affected over 37 million individuals in Brazil, resulting in 690,000 deaths [1]. Survivors of acute COVID-19 often experience prolonged symptoms, including fatigue, dyspnea, cough, anosmia, concentration difficulties, memory deficits, and mental health issues (anxiety, depression, and post-traumatic stress), potentially impacting quality of life and healthcare expenditures [2,3].

The precise occurrence of prolonged symptoms post-COVID-19 remains unknown. According to the World Health Organization (WHO) estimates, 10–20% of SARS-CoV-2 infected patients may exhibit persistent symptoms, termed post-COVID-19 condition (or long COVID) [4]. Published observational studies indicate varying prevalence rates (6–45%) influenced by COVID-19 severity, predominant variants, vaccination coverage, effective treatments during the study period, follow-up duration, and confounding factors like pre-existing frailty and comorbidities [5–7]. A Dutch prospective cohort study with over 76,000 participants found a 21% prevalence of prolonged symptoms attributable to COVID-19, 90–150 days post-infection—a result consistent with the WHO estimate [8]. However, robust data on post-COVID-19 in Brazil, particularly amid the Omicron variant dominance, high vaccination coverage, and effective acute COVID-19 treatments, are scarce, hindering the implementation of effective health policies for prevention and early rehabilitation.

Data on the impact of post-COVID-19 on relevant outcomes are limited and heterogeneous [9]. Although some observational studies associate COVID-19 consequences with reduced quality of life [10–12], these findings are susceptible to selection biases, confounding, and measurement issues due to subjective post-COVID-19 definitions. Similarly, while certain studies identify female gender, comorbidities, acute COVID-19 severity, and social deprivation as potential risk factors for post-COVID-19 [13], these findings vary and may be influenced by local factors. Local studies are necessary to identify populations at risk for post-COVID-19.

To facilitate diagnosis and research, the WHO defined post-COVID-19 in October 2021, requiring symptoms emerging within three months post-infection, lasting at least two months, and unexplained by an alternative diagnosis [4]. However, studies evaluating incidence, clinical outcomes, and potential risk factors according to the WHO definition are scarce.

To address the evidence gaps, this protocol study aims to describe the methods and design intended to assess the incidence, associated factors, and impact of post-COVID-19 condition in adults infected with SARS-CoV-2 during the Omicron phase in Brazil, using the WHO case definition.

## Methods

**Fig 1:**
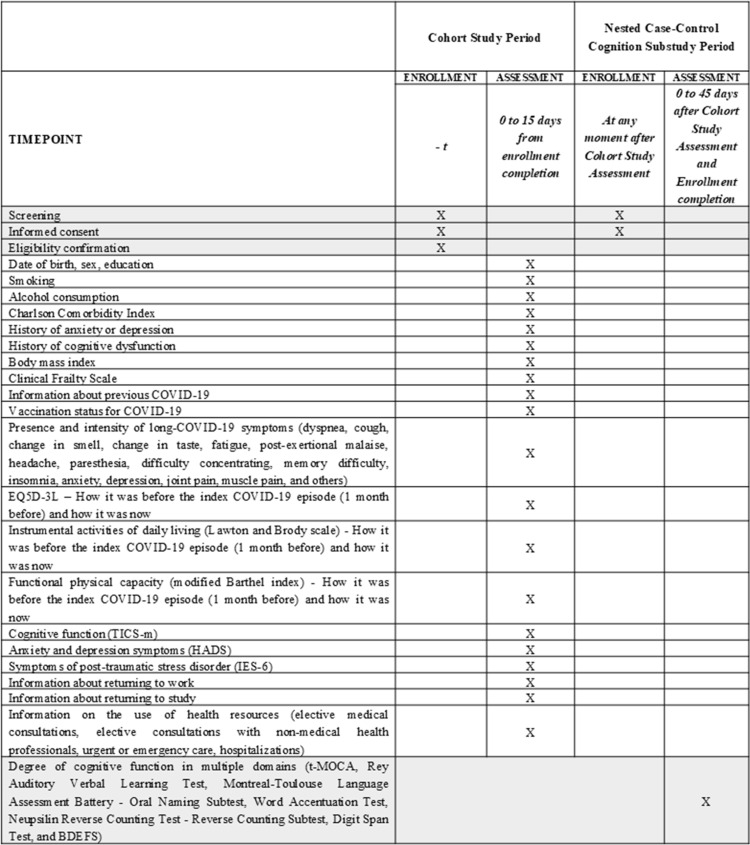
SPIRIT Study Schedule. BDEFS, Barkley Deficits in Executive Functioning Scale; EQ-5D-3L, 5-dimensional, 3-level EuroQol scale; HADS, Hospital Anxiety and Depression Symptom Scale; IES-R, Revised Event Impact Scale; N/A, not applicable; TICS-M, modified cognitive status assessment telephone interview; tMOCA, Montreal Telephone Cognitive Assessment.

We use the Strengthening the Reporting of Observational Studies in Epidemiology (STROBE) [14] and the Standard Protocol Item: Recommendations for Interventional Trials (SPIRIT) [15] as guidelines since both have items of relevance to protocol development. The study plan is available in ClinicalTrials.gov under the record NCT05822193. The detailed study protocol approved by the Ethics Committee is available in the **S1 File**. Version 4 is the last approved. Fig 1 presents the SPIRIT [15] study schedule and the SPIRIT Checklist is available in the **S2 File**.

### Study design

**Fig 2:**
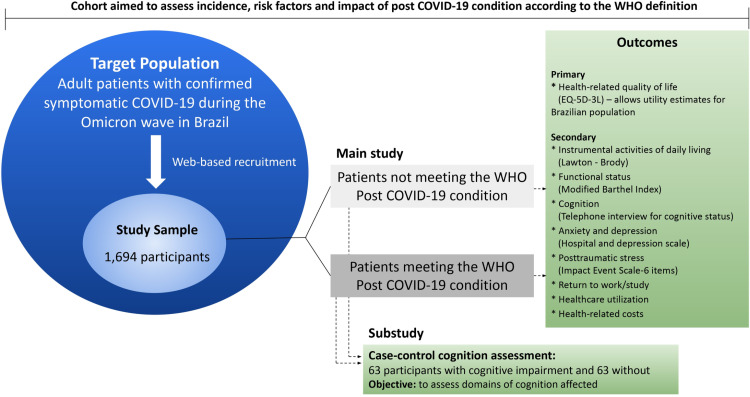
Study design.

This is a Brazilian cohort study with virtual recruitment and a nested case-control study design; the study design is presented in Fig 2. The cohort study will assess the incidence, impact, and factors associated with post-COVID-19 conditions. The nested case-control study will compare cognitive domains between post-COVID-19 cognitive dysfunction cases and controls without post-COVID-19 cognitive dysfunction, matched for age, gender, level of education, and time since COVID-19 diagnosis.

### Setting

The study will recruit participants from the 5 geopolitical regions of Brazil through public announcements on electronic websites and social media. Participant sampling will be stratified by region of residence (North, Northeast, Southwest, Midwest, and South) and age group (≥ 18 to < 40 years; ≥ 40 to < 60 years; ≥ 60 years), as described in Table 1. Stratification by age group and Brazilian geopolitical regions was implemented to balance demographic variations and reduce potential biases in participant selection.

**Table 1:** Recruitment extracts according to region of residence and age group.

Age group	Brazilian Region				
	North	Northeast	Southwest	Central-West	South
≥ 18 to < 40 years	4.4%	12.7%	18.4%	4.1%	6.9%
≥ 40 to < 60 years	2.6%	8.7%	14.7%	2.7%	4.9%
≥ 60 years	1.1%	4.7%	9.4%	1.3%	3.3%

Age stratification aims to balance health status, risk exposure, and healthcare utilization, enhancing study validity. This approach was previously used in a Brazilian public health research for prioritizing COVID-19 vaccination [16]. The age group ≥ 60 years aligns with the Brazilian definition of elderly individuals, a threshold commonly used in epidemiological research due to its association with increased health risks and healthcare needs. Adult age strata were further divided to capture the diversity of health and social experiences within adulthood.

### Participants

The study’s disclosure and recruitment processes will take place through a dedicated website providing information about the study’s objectives, eligibility criteria, risks and benefits of participation, and research procedures. Individuals expressing interest in study participation will register with basic contact information and will be remotely contacted by researchers for guidance on the study and the administration of the informed consent form (ICF).

For the nested case-control study, a sample of post-COVID-19 cognitive dysfunction cases and controls without post-COVID-19 cognitive dysfunction will be selected from the cohort. Cases and controls will be matched by age, gender, level of education, and time since COVID-19 diagnosis (± 30 days). Potentially eligible individuals for the nested case-control study, who consented to be contacted for this sub-study if selected, will be remotely contacted for guidance on the nested study and the application of a specific ICF.

The inclusion and exclusion criteria are described below

### Inclusion criteria

- Age 18 years or older;
- Residency in Brazil;
- History of symptomatic SARS-CoV-2 infection confirmed by reverse transcription-polymerase chain reaction (RT-PCR) or SARS-CoV-2 antigen test after January 1, 2022. Symptomatic SARS-CoV-2 infection is defined as an infection occurring in the presence of at least one of the following symptoms: fever, nasal congestion, rhinorrhea, anosmia, sore throat, hoarseness, cough, dyspnea, wheezing, and myalgia. The diagnosis must be confirmed by a report issued by the test site or by registration in “ConecteSUS/MeuSUSDigital”, either of these options provided or reported by the participant.

### Exclusion criteria

- Time between onset of COVID-19 symptoms and recruitment < 90 days;
- Lack of availability or inability to participate in remote interviews;
- Communication difficulties (aphasia, severe hearing impairment, severe dementia, non-native Portuguese speakers);
- Lack of consent to participate in the study;
- Participants already included in the study.

### Variables and outcomes

To assess the impact of post-COVID-19 condition on clinical outcomes we define post-COVID-19 condition according to the WHO definition: Symptoms that arise within three months of infection, lasting at least two months, and cannot be explained by an alternative diagnosis [4]. Reported post-COVID-19 condition symptoms will be collected and described by specific symptom type (dyspnea, cough, altered sense of smell, altered taste, fatigue, post-exertional malaise, headache, paresthesia, difficulty concentrating, memory impairment, insomnia, anxiety, depression, joint pain, muscle pain, and others) and affected system (neurological, mental health, respiratory, constitutional, or others). If the participant reports dyspnea, it will be quantified using the Brazilian version of the Medical Research Council Dyspnea Scale [17]. If the participant reports fatigue, it will be quantified using the Brazilian version of the Fatigue Severity Scale [18].

The exposure variables for assessing factors associated with post-COVID-19 condition are:

- Sociodemographic variables: age, gender, education, income, smoking, alcohol consumption;
- Pre-COVID-19 health conditions: comorbidities according to the Charlson Comorbidity Index (CCI) [19], history of anxiety or depression, history of cognitive dysfunction, body mass index, and frailty according to the Clinical Frailty Scale (CFS) [20]. The CCI is a scoring system that assesses the impact of comorbidities on the patient’s health. Scores range from 0 to 33, with higher scores indicating greater comorbidity. In this study, for categorization purposes, we will classify patients with scores greater than or equal to 1 as having comorbidities. We will adopt the classification of high comorbidity for scores greater than or equal to 2 [21].

The CFS is a widely used instrument to assess the level of frailty in individuals. The scale ranges from 1 to 9, where: 1. Very fit; 2. Fit; 3. Managing well; 4. Living with very mild frailty; 5. Living with mild frailty; 6. Living with moderate frailty; 7. Living with severe frailty; 8. Living with very severe frail; 9. Terminally ill. For categorization purposes, patients with a score equal to or greater than 5 will be classified as frail [19].

- History of symptomatic and documented SARS-CoV-2 infection prior to the studied COVID-19 episode;
- COVID-19 vaccination status up to the date of the studied SARS-CoV-2 infection episode;
- Severity of the studied COVID-19 episode according to the worst level of the WHO Clinical Severity Scale [22];
- Treatments used during the acute phase of COVID-19, including antivirals (molnupiravir, nirmatrelvir/ritonavir, remdesivir), corticosteroids (dexamethasone, prednisone, prednisolone, methylprednisolone, and hydrocortisone), immunomodulators (tocilizumab, baricitinib), and monoclonal antibodies against SARS-CoV-2 (casirivimab/imdevimab, bamlanivimab/etesevimab, regdanvimab, and sotrovimab).

For the nested case-control study, the exposure variable for comparing cognitive domains between participants with and without post-COVID-19 cognitive dysfunction is the Brazilian version of the modified Telephone Interview for Cognitive Status (TICS-m). The cases will be defined by a TICS-m total score ≤ 21 points [23];a total score > 21 points will be defined as control. Case-control matching will be done by age, gender, education level, and time since COVID-19 diagnosis.

### Primary outcome

The primary outcome is the health-related quality of life utility score assessed through the Brazilian version of the EuroQol 5-Dimensions and 3-Levels scale (EQ-5D-3L) [24]. The EQ-5D-3L comprises a descriptive system with five dimensions of quality of life (mobility, self-care, usual activities, pain/discomfort, and anxiety/depression). The utility score in the Brazilian population ranges from -0.17 to 1.0, with higher scores indicating better quality of life [25]. The clinically significant minimum difference varies from 0.03 to 0.52 [26].

### Secondary outcomes

The secondary outcome measures include the variables described below:

- Degree of instrumental activities of daily living (use of the telephone, mobility, shopping, responsibility for one’s medications, finances, and ability to handle finances) measured by the Brazilian version of the Lawton & Brody Instrumental Activities of Daily Living Scale [27];
- Degree of functional physical capacity measured by the Brazilian version of the Barthel Index [28];
- Degree of cognitive function measured by the Brazilian version of the Telephone Interview for Cognitive Status (TICS-m) [23];
- Symptoms of anxiety and depression measured by the Brazilian version of the Hospital Anxiety and Depression Scale (HADS) [29];
- Symptoms of post-traumatic stress measured by the Brazilian version of the Impact of Event Scale - 6 items (IES-6) [30,31];
- Return to work;
- Return to studies;
- Utilization of health resources (elective medical appointments, elective appointments with non-medical health professionals, urgent or emergency care, hospitalizations reported by the participant).

### Secondary outcomes assessed in participants selected for the nested case-control study

- Degree of cognitive function measured by the Brazilian version of the Montreal Cognitive Assessment by Telephone (tMoCA) [32];
- Degree of memory function measured by the Brazilian version of the Rey Auditory Verbal Learning Test [33];
- Degree of language function measured by the Brazilian version of the Montreal-Toulouse Language Assessment Battery - Oral Naming Subtest [34];
- Degree of intelligence function measured by the Brazilian version of the Word Accentuation Test [35];
- Degree of attention function measured by the Brazilian version of the Neupsilin Reverse Counting Test - Reverse Counting Subtest [36];
- Degree of working memory function measured by the Digit Span [37];
- Degree of executive disfunction measured by the Barkley Deficits in Executive Functioning Scale (BDEFS) [38].

### Data measurements, safety, and monitoring

Participants included in the cohort study will be assessed in a single interview by a trained researcher. The interview will take place within a window of 0–15 days after the participant signs the informed consent form (ICF). Participants selected for the nested case-control study will undergo an additional interview to assess cognitive domains up to 45 days after cohort study assessment completion, provided they initially consent to be contacted and after signing a specific ICF for this sub-study. All interviews will be conducted remotely (via telephone or video call), structured, and centralized by INOVA Research researchers trained for data collection in this modality and qualified to administer the specific tests for the nested case-control study. The cohort study interview is estimated to last 50 minutes, and the nested case-control study interview is estimated to last 45 minutes. Fig 1 also summarizes the variables that will be collected in the cohort study and substudy.

Data collection will be conducted using electronic clinical forms filled out by the researcher conducting the telephone interview based on the participant’s reported data. The tool to be used for data collection and management is the Otus platform (https://site.otus-solutions.com.br/). Otus platform allows conducting the phone calls and the interview audio recording through secure direct connection with Twilio Voice system (www://https://www.twilio.com/en-us/voice). Access to the data systems will be granted to each team member through personal and non-transferable usernames and passwords, following proper delegation within the study by the responsible coordinator researcher. Platform users (research team) will have specific permissions related to their role and delegation in the study. Several procedures will be employed to ensure data security and quality. All researchers will undergo training before the study begins on good clinical practices and study procedures, including data collection. Processes related to data management will comply with the Brazilian General Data Protection Law (LGPD; Law No. 13,709, of August 14, 2018) [39].

Automated daily backups of the database will be performed. Audio files will be stored in an anonymized manner on a server with the same security system as the database described above. Data extraction for statistical software will be automated, with data anonymization for data consistency checks, monitoring actions, derivation of variables, and statistical analyses. Responsible coordinator researcher will monitor study progress and quality by detailed monthly reports on screening, inclusion, follow-up, data consistencies, and completeness, taking immediate actions to address any issues.

### Data quality control

To ensure data accuracy, consistency, and reliability, a comprehensive quality control strategy will be implemented throughout the study. Data processing and analysis will be performed using R [40], employing established packages for data cleaning, validation, and analysis.

All the case report forms will be reviewed daily for completeness. Missing data will be evaluated directly in the data entry software (Otus), which will allow for the addition of comments where necessary and ensure that the reasons for missing values are properly documented. Validation rules will be applied to numeric variables to prevent out-of-range or illogical values from being entered.

Every two weeks, a subset of 20% of the data related to the period will be selected at random and reviewed by an independent quality control team to detect any inconsistencies or biases that may have been overlooked during the data collection process. The quality control team will audit source data by listening to the recorded interviews. Furthermore, the duration of interviews will be monitored to detect any potential discrepancies or inconsistencies in the data collection process. Significant variations in interview time across different interviewers will be flagged and reviewed to ensure consistency. To assess the internal consistency of the data, a correlation analysis of the scores assigned by each interviewer will be performed without comparing the specific scores to avoid external influences. If more than 50% of inconsistency was detected in any strategy or in the sum of them, another 20% subset of the data will be selected at random for the same process.

Furthermore, to enhance the reproducibility and transparency of the data analysis process, the R code used for data cleaning, processing, and statistical analysis will be thoroughly evaluated. This code will be reviewed for accuracy, efficiency, and consistency to ensure it aligns with the predefined research protocols. By conducting regular reviews of the analysis code, we will minimize coding errors and maintain the integrity of the data processing workflow.

### Sample size

Considering an incidence of post-COVID-19 condition of 20% [4], a sample size of 1,540 participants with a history of COVID-19 is estimated to allow the detection of a difference greater than or equal to 0.05 in EuroQol 5-Dimensions and 3-Levels (EQ-5D-3L) quality of life utility scores (within the range of minimally clinically significant difference) [26] between patients with post-COVID-19 condition and participants without post-COVID-19 condition, with a power of 80%, a two-tailed alpha of 0.05, and a standard deviation of the utility score of 0.28. To account for potential uncertainties related to post-COVID-19 quality of life parameters and to address the loss of power due to the need for covariate adjustment, the sample was inflated by 10%. Thus, the present cohort study intended to recruit 1,694 participants.

For the nested case-control study, a sample size of 126 participants (63 cases of post-COVID-19 cognitive dysfunction and 63 controls without post-COVID-19 cognitive dysfunction) will allow the detection of a mean difference with an effect size of 0.05 (considered moderate according to Cohen) in cognitive function scores.

### Statistical methods

Continuous variables will be expressed as mean and standard deviation or as median and interquartile range, while categorical variables will be expressed as absolute and relative frequencies. Data normality will be assessed by histogram inspection.

The comparison of EQ-5D-3L utility scores (primary outcome) between patients with and without post-COVID-19 condition will be performed using adjusted generalized linear models for age, gender, comorbidities, frailty, and region of residence (North, Northeast, Southwest, Midwest, and South). Adjusted results for EQ-5D-3L utility scores and questionnaire domains (mobility, self-care, usual activities, pain/discomfort, and anxiety/depression) will be summarized for each comparison group using central tendency and dispersion measures, along with the mean or median difference as a measure of effect size. The comparison of secondary outcomes between patients with and without post-COVID-19 condition will be performed using the same model as the primary outcome. The effect measure used will be absolute difference (for categorical outcomes) and mean or median difference (for continuous outcomes).

The assessment of factors associated with post-COVID-19 condition will be conducted using generalized linear models considering a Poisson distribution with robust variance. Variables with a p-value < 0.20 will be included in the multivariable model using a forward procedure. The association result of variables with post-COVID-19 condition will be described through relative risk.

The comparison between cognition domain test scores among cases of post-COVID-19 cognitive dysfunction and controls without post-COVID-19 cognitive dysfunction will be performed using generalized linear models. The effect measure used will be mean or median difference.

A statistical significance level of 0.05 will be considered for all comparisons. 95% confidence intervals will be described for all effect measure analyses, without adjustment for multiplicity. Analyses will be conducted using R software, with the version to be detailed at the time of analysis [40].

### Sensitivity analysis

To explore potential regional variations, we will conduct a sensitivity analysis stratified by geopolitical region. This analysis will evaluate whether the primary associations remain consistent across different subgroups.

### Ethics, safety and dissemination

The research has been planned and will be conducted in accordance with Resolution 466 of December 12, 2012, from the National Health Council [41], the Brazilian Law that regulates research with human beings (Law 14874/2024) [42], the International Council for Harmonisation’s Good Clinical Practice Guidelines, Amendment 6 – Revision 2 (ICH) [43], in addition to the standards recommended by the Brazilian General Data Protection Law (LGPD) [39]. This research also follows the guidelines for procedures in research with any stage in a virtual environment, as described in Circular Letter No. 1/2021 from CONEP dated March 3, 2021 [44].

The study was approved on April 18, 2023, by the Moinhos de Vento Hospital Ethics Committee, under the registry number CAAE 68343923.7.0000.5330, in the approval opinion number 6.008.948.

Informed Consent Form (ICF) will be presented to research participants at the time of the invitation to participate in the study. Considering the diversity of generations and their preferences, ease, or limitations regarding the use of technologies, the consent process may occur independently or assisted. In independent consent, the digital Informed Consent Form (e-ICF) will be provided to individuals interested in participating in the study at the initial contact moment. The e-ICF will be provided through an individual link, by email, or text message, according to the potential participant’s preference. In the assisted process, this will occur if the participant expresses a desire for assistance or additional clarification. In these cases, the individual will have the option to communicate with one of the researchers via text message or phone call (whichever the individual prefers) to clarify doubts or the electronic consent registration process. Participants who consent to be contacted for participation in the nested case-control study, if selected, will only participate in this sub-study if they voluntarily consent after reading a specific e-ICF for this study, following the same consent registration procedures.

All participant personal information will remain confidential, accessible only to the study team to prevent breaches of confidentiality. At no point will the participant’s name or any health-related information be disclosed to individuals outside the study team engaged in activities requiring access to such information. Appropriate measures to safeguard participant identity and confidentiality will be implemented, including restricted access to study documents for research personnel only and the storage of electronic data in a secure database.

Study results will be disseminated in aggregated form for academic and scientific purposes, ensuring no disclosure of any data that could reveal participants’ identities. Our dissemination plan includes both scientific and lay audiences. The scientific dissemination involves presenting findings at scientific conferences and publishing in peer-reviewed journals. The decision on data publication and the target journal lies within the purview of the study’s steering committee. For lay audiences dissemination, a summary of results in Portuguese and accessible language will be directly communicated to participants via email and made available on the recruitment website (www.covidlonga.com.br). In order to improve the general public access to study results, we will collaborate with local health institutions and health-related government entities. The release of these results will occur only after the study’s conclusion.

To be credited as an author of resulting articles, researchers must meet all four criteria recommended by the International Committee of Medical Journal Editors. Regarding data sharing policy, requests for data sharing will undergo evaluation by the study’s steering committee.

## Study status and expected results

The study design and protocol were finalized in January 2023. The first participant was included on July 21, 2023. Currently the study is recruiting participants in all the Brazilian geopolitical regions. Recruitment will be completed until July 2025. From July 21, 2023, until February 15, 2025, 1,221 participants were enrolled from 1,694 needed. In the nested case-control, 77 participants were enrolled from 126 needed.

We anticipate a substantial incidence of post-COVID-19 condition (Long COVID) among adults infected during the Omicron wave in Brazil, potentially exceeding rates reported in some European and North American countries, given Brazil’s high COVID-19 case numbers. We hypothesize that several factors will be associated with post-COVID-19 condition, including the severity of acute infection, even within the Omicron context, pre-existing comorbidities, and vaccination status. The limited availability of comprehensive post-COVID rehabilitation programs in Brazil raises concerns about the potential negative impact of the condition on affected individuals’ health-related quality of life, as well as the other physical and psychological components assessed in this study. In the planned case-control study, we expect a higher incidence of post-COVID-19 condition in the case group.

## Discussion

We anticipate that our study will contribute significantly to elucidating the prevalence of post-COVID syndrome within the Brazilian population. The comprehensive core outcome setting employed, designed to be both broad and sensitive in capturing impacts on quality of life, functionality, and cognitive aspects, aims to provide a nuanced understanding of the true burden of this pathology in the Brazilian community and, to some extent, in the international community.

Our focus on adjusting results for key confounding factors, particularly addressing the social disparities among different Brazilian states, is critical. These disparities have established connections with infectious diseases [45], and our study seeks to account for them not only through inclusion in the multivariate model but also through a meticulous process of stratified sampling that considers the geographic diversity of participants.

Furthermore, our commitment to employing a modernized and digitized data collection design, along with a streamlined digital consent process, represents a pioneering phase in population-based studies. The implementation of automated mechanisms from the initiation to the conclusion of the study is poised to enhance efficiency, accuracy, and participant engagement, setting a new standard for the methodology of large-scale epidemiological investigations.

As we await the outcome of our analyses, we anticipate that the results will not only deepen our understanding of the post-COVID syndrome’s impact on the Brazilian population but also shed light on the broader implications for public health, especially concerning infectious diseases and social disparities. This study aims to provide a robust foundation for informed decision-making and the development of targeted interventions to mitigate the long-term effects of COVID-19 on individuals and communities.

## Limitations

Common limitations associated with retrospective observational and case-control studies may influence the interpretability of our findings. The inherent nature of these study designs poses challenges in establishing causation, and the reliance on historical data introduces the possibility of recall bias. Additionally, confounding variables may not be entirely accounted for, potentially affecting the internal validity of our results. Despite meticulous efforts to address these limitations, there remains a degree of uncertainty inherent in retrospective designs, emphasizing the need for cautious interpretation.

Virtual studies, while offering certain advantages, may introduce limitations and biases, such as restricting participation to digitally literate individuals and potentially affecting comprehension of survey questions. To mitigate these challenges, we implemented some strategies. These included broad dissemination efforts, selection of easily understood and widely used instruments adapted for telephone interviews, recruitment of experienced health sector interviewers familiar with diverse population interaction, and rigorous standardization of the interview process through systematic training and monitoring. Another standardization aspect is that the main interview is being conducted only by telephone interview, while the substudy cognition battery is being applied only through video calls.

Recognizing the inherent challenges of retrospective observational and case-control studies, our research team brings experience, particularly in navigating the complexities associated with these study designs. Proactively addressing the limitations mentioned, we have implemented robust and automated procedures. The process of collecting and managing data digitally offers several benefits over non-automated processes, including standardization, reliability, and the security of collected data. This approach is designed not only to enhance the reliability of our results but also to align with the principles of the Brazilian LGPD and international ethical guidelines. By implementing stringent measures throughout the study, we aim to minimize biases, strengthen the validity of our findings, and uphold the highest standards of research integrity.

The study sample was designed to ensure adequate representation across different geopolitical regions and age groups, aiming to reflect the diversity of the population. Stratification by age and region was implemented to balance demographic variations and reduce potential biases in participant selection. While these stratifications enhance the study’s internal validity, we acknowledge that representativeness does not guarantee full generalizability. The distribution of participants across regions and age groups will be considered in the interpretation of findings, and potential limitations related to population representativeness will be carefully discussed.

## Conclusion

In conclusion, our study, conducted in the unique context of the Omicron phase in Brazil, aims to address critical gaps in the understanding of post-COVID-19 conditions. By employing the World Health Organization’s case definition and adhering to STROBE and SPIRIT guidelines, we have meticulously designed a cohort study with nested case-control elements to investigate the incidence, associated factors, and impact of post-COVID-19. Our emphasis on recruiting a diverse sample from all geopolitical regions of Brazil, stratified by age groups, enhances the generalizability of our findings and provides a comprehensive picture of the post-COVID-19 landscape.

Our findings will not only contribute to the global discourse on post-COVID-19 syndrome but will also offer specific insights into the Brazilian context. By utilizing a multifaceted approach that includes assessing cognitive domains and employing a robust outcome setting, we aim to capture the nuanced impact of post-COVID-19 on individuals’ lives. The meticulous adjustment for social disparities among Brazilian states and the integration of modernized, digitized data collection methods further underscore our commitment to producing rigorous and applicable results.

## Supporting information

S1 File Study protocol version 4. (PDF)

S2 File SPIRIT Checklist. (PDF)
