# Supplementary material for: Incidence, associated factors and impact of the post-COVID-19 condition in Brazil: Study protocol of an observational cohort during the Omicron phase
Source: PLoS One. 2025 Apr 24;20(4):e0322466. doi: 10.1371/journal.pone.0322466 (PMC12021252; doi:10.1371/journal.pone.0322466)
Supplement: S1 File — (PDF) [file pone.0322466.s001.pdf]

STUDY PROTOCOL

Incidence, associated factors and impact of the post-COVID-19 condition in Brazil during the Omicron phase: an observational study

Long COVID Brazil Study

Version 4, April 20, 2024.

Submitted by: INOVA Research

This document is confidential, and its use, reproduction, dissemination, and publication are restricted to the responsible researchers at INOVA Research – INOVA MEDICAL, the sponsor and the financial supporter.

VERSIONS HISTORY

Version, date	Main changes
**1, March 2, 2023**	Initial version
**2, July 31, 2023**	• Clarification regarding the mandatory nature of laboratory confirmation of COVID-19 by RT-PCR or antigen test, but the non-mandatory requirement for participants to submit a supporting document.<br>• Replacement of the 22-item Impact of Event Scale Revised (IES-R) for the assessment of post-traumatic stress symptoms with the shorter, validated 6-item IES-6. The use of the reduced version was preferred to improve interview efficiency, i.e., to obtain comparable results with less time required from the participant. The IES-6 has been used since the beginning of recruitment, after notification to the Ethics Committee. Addition of the reference for the validation of the reduced version and the respective cut-off point: Thoresen S, Tambs K, Hussain A, Heir T, Johansen VA, Bisson JI. Brief measure of posttraumatic stress reactions: impact of event scale-6. Soc Psychiatry Psychiatr Epidemiol. 2010;45:405–12.<br>Appendix 2: Test results report template, adjusted for _v2_31_jul_2023, reflecting the instrument substitution.<br>• Items inclusion in the registry of interest in participating in the study (full name, pronoun, city). Appendix_1_Recruitment_and_dissemination_plan adjusted for _v2_31_jul_2023, and updating of dissemination materials.<br>• Modification to record consent in the electronic Informed Consent Form (e-ICF), instead of the digital signature process.<br>• Revision of the data registry form with the removal of items not applicable to the intended analyses.<br>• Reduction in the average interview duration time from 1 hour (60 minutes) to 50 minutes, resulting from the revision of the data collection form and the use of the reduced instrument for the assessment of post-traumatic stress symptoms (IES-6).
**3, September 15, 2023**	• Correction of the Digits subtest version and inclusion of the Barkley Deficits in Executive Functioning Scale (BDEFS) in the battery of assessments that constitutes the interview for the cognition sub-study.<br>• Revision of the data collection form in the session related to the cognition sub-study to include the corrected item and the added item, as well as the order of the cognitive tests.
**4, April 20, 2024**	• Correction of the cutoff point used to define cases and controls in the cognition assessment sub-study, through the score of the modified Telephone Interview for Cognitive Status (TICS-m), from 31 to 21 (a typographical error).<br>• Inclusion of an in-person recruitment modality (initial contact, invitation, and consent process) conducted by local recruiters trained by the study team. Appendix_1_Recruitment_and_dissemination_plan adjusted for v3_20_abr_2024, reflecting this update.<br>• Inclusion of year 2024 as a possible date for COVID-19 cases and vaccination date in the data collection form.

ADMINISTRATIVE INFORMATIONS

Title

Incidence, associated factors and impact of the post-COVID-19 condition in Brazil during the Omicron phase: an observational study

Acronym

Long COVID Brazil Study

Principal Investigators

Maicon Falavigna, MD, MSc, PhD

Link to *Lattes* resume: http://lattes.cnpq.br/6242018987560550

Regis Goulart Rosa, MD, MSc, PhD

Link to *Lattes* resume: http://lattes.cnpq.br/5047175130077041

Collaborators

Caroline Cabral Robinson, MSc, PhD

Link to *Lattes* resume: http://lattes.cnpq.br/4097704323332933

Daniel Sganzerla, BSc

Link to *Lattes* resume*:* http://lattes.cnpq.br/3781964610442096

Josélia Larger Manfio, PhD

Link to *Lattes* resume*:* http://lattes.cnpq.br/5736470882499170

Proposing Institution

INOVA Research, a department of INOVA MEDICAL – HEALTH SERVICES AND MANAGEMENT LTD

Sponsor

INOVA MEDICAL – HEALTH SERVICES AND MANAGEMENT LTD, Brazil

Founding

Merck Investigator Studies Program Research Grant

Protocol version

Version 4, dated April 20, 2024.

GLOSSARY OF TERMS

BDEFS	Barkley Deficits in Executive Functioning Scale
COVID-19	Coronavirus Disease caused by a SARS-CoV-2 infection
EQ-5D-3L	EuroQol 5-dimensions 3-level scale
HADS	Hospital Anxiety and Depression Scale
ICF	Informed Consent Form
ICH	International council of harmonization
ICMJE	International committee of medical journal editors
IES-6	Impact of Event Scale - 6 items
LGPD	Brazilian General Data Protection Law
MISP	Merck investigator studies program
MRC	Medical Research Council
RT-PCR	Reverse transcriptase-polymerase chain reaction
SARS-CoV-2	Severe Acute Respiratory Syndrome Coronavirus 2.
TICS-m	Telephone Interview for Cognitive Status
tMoCA	Telephone Montreal Cognitive Assessment
WHO	World Health Organization

TABLE OF CONTENTS

ADMINISTRATIVE INFORMATIONS 3
GLOSSARY OF TERMS 4
TABLE OF CONTENTS 5
ABSTRACT 5
INTRODUCTION 10
RATIONALE 13
OBJECTIVES 14
Primary objective 14
Secondary objectives 14
METHODS 15
Study design 15
Study participants recruitment 15
Eligibility criteria 16
Exposures 17
Outcomes 19
Study procedures 20
Data management 22
Data integrity 23
Sample size 23
Statistical analysis 24
ETHICS AND GOOD CLINICAL PRACTICES 25
Procedures for participant consent 25
Potential risks and benefits 26
STUDY ORGANIZATION 28
Study coordination 28
Steering Committee 28
Sponsor 28
Funding 29
DISSEMINATION POLICY 30
Dissemination plan 30
Authorship Policy 30
Data sharing policy 30
REFERENCES 31

ABSTRACT

**Title**	Incidence, associated factors and impact of the post-COVID-19 condition in Brazil during the Omicron phase: an observational study
**Objectives**	**Primary objective**<br>● To assess the association between post-COVID-19 condition, according to the World Health Organization (WHO) definition, and health-related quality of life in adults with a history of symptomatic SARS-CoV-2 infection during the Omicron phase in Brazil<br><br>**Secondary objectives**<br>● To evaluate the incidence of post-COVID-19 condition according to the WHO case definition in adults during the Omicron phase in Brazil.<br>● To describe the reported symptoms of post-COVID-19 condition.<br>● To assess potential risk factors for post-COVID-19 condition in adults with a history of symptomatic COVID-19 during the Omicron phase in Brazil.<br>● To evaluate the association between post-COVID-19 condition, according to the WHO definition, and the following outcomes in adults:<br>1) Instrumental activities of daily living,<br>2) Physical functional capacity,<br>3) Cognition,<br>4) Anxiety symptoms,<br>5) Depression symptoms,<br>6) Post-traumatic stress symptoms,<br>7) Return to work activities,<br>8) Return to study activities,<br>9) Healthcare resource utilization and costs.<br>● To compare cognitive domains between cases of post-COVID-19 cognitive dysfunction and controls without post-COVID-19 cognitive dysfunction, paired by age group, sex, education level, and time since COVID-19 diagnosis (nested case-control study).
**Study design**	A national cohort study with virtual-based recruitment to assess the incidence, impact, and associated factors of post-COVID-19 condition with a nested case-control study to compare cognitive domains between cases of post-COVID-19 cognitive dysfunction and controls without post-COVID-19 cognitive dysfunction.

**Recruitment**	The cohort study will recruit participants through online advertisements, social media and active search for prospective participants. For the nested case-control study, a sample of cases and controls paired by age group, sex, education level, and time since COVID-19 diagnosis will be selected from the cohort.
**Participants**	**Inclusion Criteria**<br>● Age 18 years or older<br>● Residency in Brazil<br>● History of symptomatic* SARS-CoV-2 infection confirmed by a reverse transcriptase-polymerase chain reaction (RT-PCR) or SARS-CoV-2 antigen test after January 1, 2022<br>*At least one of the following symptoms: fever, nasal congestion, rhinorrhea, anosmia, sore throat, hoarseness, cough, dyspnea, wheezing, and myalgia.<br><br>**Exclusion Criteria**<br>● Time between onset of COVID-19 symptoms and recruitment < 90 days<br>● Lack of availability or inability to participate in remote interviews<br>● Communication difficulties (aphasia, severe hearing impairment, severe dementia, non-native Portuguese speakers)<br>● Lack of consent to participate in the study<br>● Participants already included in the study
**Exposures**	**Exposure variable for assessing the impact of post-COVID-19 on clinical outcomes:**<br>Presence of post-COVID-19 condition according to the WHO definition*<br>*Symptoms that start within three months of the SARS-Cov-2 infection and last for at least two months and cannot be explained by another health condition.<br><br>**Exposure variables for assessing factors associated with post-COVID-19:**<br>● Sociodemographic variables,<br>● Pre-COVID-19 health conditions,<br>● History of symptomatic and documented infection by SARS-CoV-2 prior to the COVID-19 episode under study,<br>● Vaccination status for COVID-19,<br>● Severity of the COVID-19 episode according to the worst level of the WHO clinical severity scale,<br>● Treatments used during the acute phase of COVID-19 (antivirals, corticosteroids, immunomodulators, and monoclonal antibodies against SARS-CoV-2).

	**Exposure variable for comparison of cognition domains between participants with and without post-COVID-19 cognitive dysfunction (nested case-control study)**<br>● Cases: Cognitive dysfunction according to the Brazilian version of the modified Telephone Interview for Cognitive Status (TICS-m; total score ≤ 21),<br>● Controls: absence of cognitive dysfunction according to the Brazilian version of the TICS-m (total score > 21) paired with cases by age group, sex, education level and time since COVID-19 diagnosis.
**Outcomes**	**Primary Outcome**<br>Health-related quality of life assessed by the utility score of the EuroQol 5-dimension 3-level scale (EQ-5D-3L).<br><br>**Secondary Outcomes**<br>● Degree of instrumental activities of daily living (use of the telephone, mobility, shopping, responsibility for one's medications, finances, and ability to handle finances) measured by the Brazilian version of the Lawton & Brody Instrumental Activities of Daily Living Scale<br>● Degree of functional physical capacity measured by the Brazilian version of the Barthel Index<br>● Degree of cognitive function measured by the Brazilian version of the Telephone Interview for Cognitive Status (TICS-m)<br>● Symptoms of anxiety and depression measured by the Brazilian version of the Hospital Anxiety and Depression Scale (HADS)<br>● Symptoms of post-traumatic stress measured by the Brazilian version of the Impact of Event Scale - 6 items (IES-6)<br>● Return to work<br>● Return to studies<br>● Utilization of health resources (elective medical appointments, elective appointments with non-medical health professionals, urgent or emergency care, hospitalizations reported by the participant)

	**Secondary outcomes assessed in participants selected for the nested case-control study**<br>● Secondary Outcomes Assessed in Participants Selected for the Nested Case-Control Study<br>● Degree of cognitive function measured by the Brazilian version of the Montreal Cognitive Assessment by Telephone (tMoCA)<br>● Degree of memory function measured by the Brazilian version of the Rey Auditory Verbal Learning Test<br>● Degree of language function measured by the Brazilian version of the Montreal-Toulouse Language Assessment Battery - Oral Naming Subtest<br>● Degree of intelligence function measured by the Brazilian version of the Word Accentuation Test<br>● Degree of attention function measured by the Brazilian version of the Neupsilin Reverse Counting Test - Reverse Counting Subtest<br>● Degree of working memory function measured by the Digit Span<br>● Degree of executive disfunction measured by the Barkley Deficits in Executive Functioning Scale (BDEFS)
**Assessment of the study variables**	For the cohort, the assessment of the study variables will be carried out through a centralized, structured remote interview (telephone or video call) conducted by trained research professionals with expertise in good clinical practices. Participants selected for the case-control study will participate in an additional remote interview for the assessment of cognitive domains, conducted by professionals qualified to conduct the tests.
**Sample size**	Cohort study: 1.694 participants.<br>Nested case-control study: 126 participants selected from the cohort study participants (63 with cognitive disfunction e 63 without cognitive disfunction).

INTRODUCTION

The infection caused by the novel coronavirus (SARS-CoV-2), named COVID-19, has already surpassed the mark of 37 million infected people in Brazil with more than 690 thousand deaths.[1] Among the survivors of acute forms of COVID-19, a significant portion presents prolonged symptoms such as fatigue, dyspnea, cough, anosmia, difficulty concentrating, memory deficit and symptoms of mental health impairment (anxiety, depression and post-traumatic stress), which have the potential to negatively impact relevant outcomes such as quality of life and healthcare costs.[2,3]

The precise occurrence of prolonged symptoms after a COVID-19 episode is unknown. According to estimates from the World Health Organization (WHO), 10 to 20% of patients infected with SARS-CoV-2 may experience persistent symptoms in a condition known as post-COVID-19 (or long COVID).[4] Observational studies published to date have shown a prevalence of prolonged symptoms ranging from 6 to 45%, depending on the patient series evaluated, with variations that can be explained by: 1) severity of COVID-19; 2) predominant variant, vaccination coverage, and availability of effective treatments for acute COVID-19 during the study period; 3) follow-up time; as well as 4) potential confounders such as pre-existing frailty and associated comorbidities.[5-7] In an attempt to estimate the occurrence of prolonged symptoms attributable to COVID-19 (after excluding cases whose symptoms could be explained by other health conditions), a prospective cohort study conducted in the Netherlands with over 76,000 participants found a prevalence of prolonged symptoms attributable to COVID-19 of 21%, 90 to 150 days after the index episode of SARS-CoV-2 infection[8] - a result close to that estimated by the WHO. Unfortunately, robust and accurate data on the occurrence of post-COVID-19 in Brazil, especially in the current scenario of Omicron variant predominance (the main cause of COVID-19 in Brazil since January 2022, Figure 1), high vaccination coverage, and availability of effective treatments for acute COVID-19, are scarce. This knowledge gap hinders the implementation of effective health policies for the prevention and early rehabilitation of post-COVID-19.

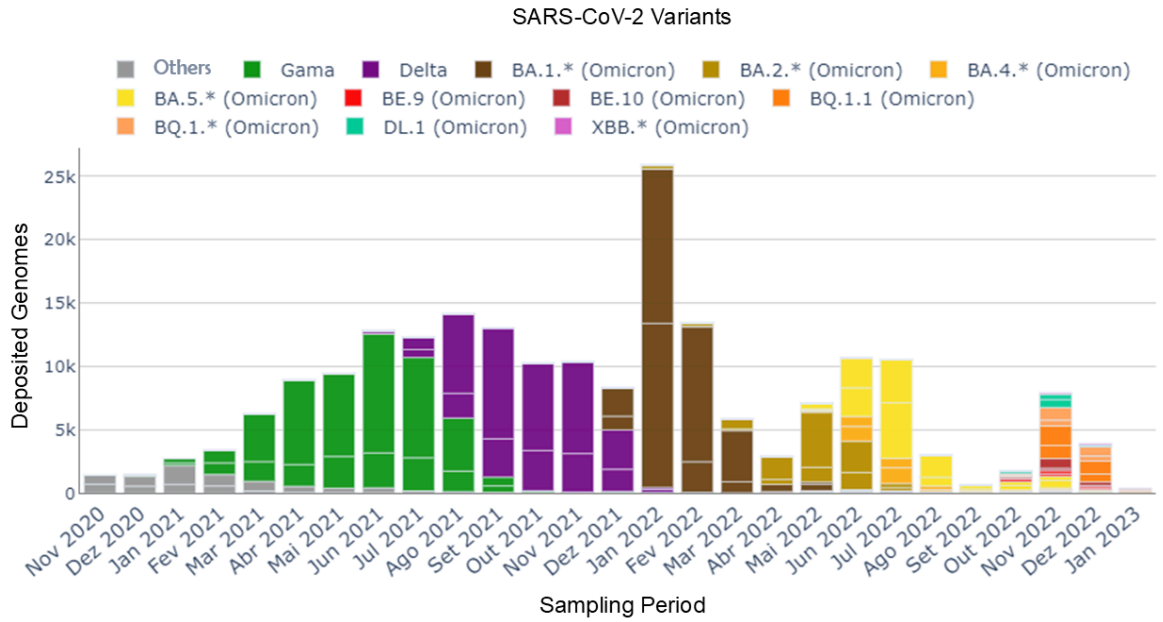

Figure 1. SARS-CoV-2 variants related with COVID-19 in Brasil. Source: Rede Genômica Fiocruz.

Data regarding the impact of post-COVID-19 on relevant outcomes is also scarce and heterogeneous.[9] Although observational studies have found an association between the consequences of COVID-19 and a lower quality of life,[10-12] this finding is susceptible to potential selection, confounding, and measurement biases, exacerbated by the use of subjective definitions of post-COVID-19. Similarly, although some observational studies have identified female sex, comorbidities, severity of acute COVID-19 episodes, and social deprivation as potential risk factors for post-COVID-19,[13] these findings are inconsistent across studies and may also be influenced by local factors such as the predominant variant, community transmission rate of SARS-CoV-2, vaccination status, and access to COVID-19 prevention and rehabilitation measures. Therefore, local studies are needed to generate knowledge about populations at risk for developing post-COVID-19.

To facilitate diagnosis and research on the topic, in October 2021, the WHO published a case definition for post-COVID-19, which was developed after a Delphi consensus process involving experts and representatives of patients and families.[4] According to the definition, post-COVID-19 refers to "symptoms that occur within three months of infection, last for at least two months, and cannot be explained by an alternative diagnosis". However, studies evaluating the incidence, impact on clinical outcomes, and potential risk factors for post-COVID-19 according to the WHO case definition are still scarce.

Therefore, to contribute to filling the evidence gaps described above, this study aims to evaluate the incidence, associated factors, and impact of post-COVID-19 according to the WHO case definition in adult patients who presented with documented SARS-CoV-2 infection during the Omicron phase in Brazil.

RATIONALE

The current context of a growing number of people presenting physical, cognitive, and mental health consequences after a COVID-19 episode, coupled with a scarcity of long-term data, has made it a priority to study the incidence, risk factors, and impact of the case definition of post-COVID-19 on clinical outcomes, especially in the current scenario of Omicron variant predominance, high vaccination coverage - albeit with limited duration of protection - and availability of effective treatments for the acute phase of COVID-19. The data from this study will contribute to the generation of evidence on this important topic, contributing to the knowledge about post-COVID-19 in Brazil and to evidence-based debate regarding potential strategies for prevention, identification, and rehabilitation of patients with post-COVID-19 sequelae.

OBJECTIVES

Primary objective

To assess the association between post-COVID-19 condition, according to the World Health Organization (WHO) definition, and health-related quality of life in adults with a history of symptomatic SARS-CoV-2 infection during the Omicron phase in Brazil

Secondary objectives

- To evaluate the incidence of post-COVID-19 condition according to the WHO case definition in adults during the Omicron phase in Brazil.
- To describe the reported symptoms of post-COVID-19 condition.
- To assess potential risk factors for post-COVID-19 condition in adults with a history of symptomatic COVID-19 during the Omicron phase in Brazil.
- To evaluate the association between post-COVID-19 condition, according to the WHO definition, and the following outcomes in adults:
  1) Instrumental activities of daily living,
  2) Physical functional capacity,
  3) Cognition,
  4) Anxiety symptoms,
  5) Depression symptoms,
  6) Post-traumatic stress symptoms,
  7) Return to work activities,
  8) Return to study activities,
  9) Healthcare resource utilization and costs.
- To compare cognitive domains between cases of post-COVID-19 cognitive dysfunction and controls without post-COVID-19 cognitive dysfunction, paired by age group, sex, education level, and time since COVID-19 diagnosis (nested case-control study).

METHODS

Study design

This is a national cohort study with virtual and in-person recruitment, with a nested case-control study (Figure 2). The cohort study will evaluate the incidence, impact, and factors associated with post-COVID-19 condition. The case-control study will compare cognitive domains between cases of post-COVID-19 cognitive dysfunction and controls without post-COVID-19 cognitive dysfunction, matched by age group, sex, education level, and time since COVID-19 diagnosis.

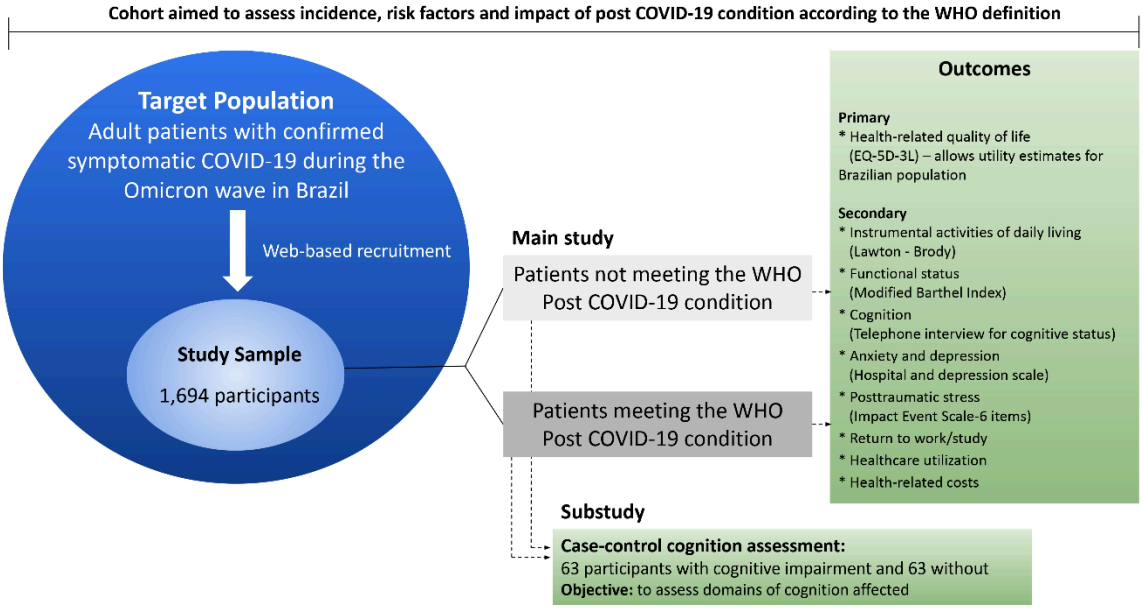

Figure 2. Study design.
EQ-5D-3L: EuroQol 5-Dimensions-3-Levels questionnaire, HADS: Hospital Anxiety and Depression Scale, IES-6: Impact of Event Scale-6 items, TICS-M: Modified Telephone Interview for Cognitive Status, tMOCA: Telephone Montreal Cognitive Assessment.

Study participants recruitment

The study will recruit participants from all the five geopolitical regions of Brazil through public announcements on websites, social media, and active in-person recruitment of prospective participants. In-person recruitment will be conducted by trained recruiters delegated by the principal investigator. The sample of participants will be stratified by region

of residence (North, Northeast, Southeast, Midwest, and South) and age group (≥ 18 to < 40 years; ≥ 40 to < 60 years; ≥ 60 years) as described in Table 1.

Table 1. Recruitment quotas according to region of residence and age group.

Age group	Brazilian Region				
	**North**	**Northeast**	**Southwest**	**Midwest**	**South**
**≥18 a <40 Years**	4.4%	12.7%	18.4%	4.1%	6.9%
**≥40 a <60 Years**	2.6%	8.7%	14.7%	2.7%	4.9%
**≥60 Years**	1.1%	4.7%	9.4%	1.3%	3.3%

The study's dissemination and recruitment processes are detailed in Appendix 1 (Recruitment and Dissemination Plan). The study will be disseminated through a webpage that will provide information about the study's objectives, eligibility criteria, risks and benefits of participation, and research procedures. In-person dissemination and active participant search will also direct interested individuals to the study webpage. Individuals who express interest in participating in the study will register with basic contact information and will be contacted remotely by researchers for study orientation and conduction of the informed consent form (ICF). Individuals who express interest through in-person contact will already receive study orientation and access to the ICF and may complete the consent process with the researcher who made the in-person contact, if they wish.

For the nested case-control study, a sample of cases with post-COVID-19 cognitive dysfunction and controls without post-COVID-19 cognitive dysfunction will be selected from the cohort. Cases and controls will be matched by age group, sex, education level, and time since COVID-19 diagnosis (± 30 days). Potentially eligible individuals for the case-control study, who consented to be contacted for this sub-study if selected, will be contacted remotely for orientation on the nested study and conduction of a dedicated ICF.

Eligibility criteria

Inclusion criteria

- Age 18 years or older
- Residency in Brazil

- History of symptomatic SARS-CoV-2 infection confirmed by reverse transcription-polymerase chain reaction (RT-PCR) or SARS-CoV-2 antigen test after January 1, 2022. Symptomatic SARS-CoV-2 infection is defined as an infection occurring in the presence of at least one of the following symptoms: fever, nasal congestion, rhinorrhea, anosmia, sore throat, hoarseness, cough, dyspnea, wheezing, and myalgia. The diagnosis must be confirmed by a report issued by the test site or by registration in "*ConecteSUS/MeuSUSDigital*", either of these options provided or reported by the participant.

Exclusion criteria

- Time between onset of COVID-19 symptoms and recruitment < 90 days
- Lack of availability or inability to participate in remote interviews
- Communication difficulties (aphasia, severe hearing impairment, severe dementia, non-native Portuguese speakers)
- Lack of consent to participate in the study
- Participants already included in the study

Exposures

Exposure variable for assessing the impact of post-COVID-19 on clinical outcomes:

- Presence of post-COVID-19 condition according to the WHO definition: Symptoms that start within three months of the SARS-Cov-2 infection and last for at least two months and cannot be explained by another health condition.[4]

  Reported post-COVID-19 condition symptoms will be collected and described by specific symptom type (dyspnea, cough, altered sense of smell, altered taste, fatigue, post-exertional malaise, headache, paresthesia, difficulty concentrating, memory impairment, insomnia, anxiety, depression, joint pain, muscle pain, and others) and affected system (neurological, mental health, respiratory, constitutional, or others). If the participant reports dyspnea, it will be quantified using the Brazilian version of the Medical Research Council Dyspnea Scale[14]. If the participant reports fatigue, it will be quantified using the Brazilian version of the Fatigue Severity Scale[15].

- History of symptomatic and documented infection by SARS-CoV-2 prior to the COVID-19 episode under study,

- Vaccination status for COVID-19,
- Severity of the COVID-19 episode according to the worst level of the WHO clinical severity scale,
- Treatments used during the acute phase of COVID-19 (antivirals, corticosteroids, immunomodulators, and monoclonal antibodies against SARS-CoV-2).

Exposure variables for assessing factors associated with post-COVID-19

- Sociodemographic variables: age, gender, education, income, smoking, alcohol consumption
- Pre-COVID-19 health conditions: comorbidities according to the Charlson Comorbidity Index[16], history of anxiety or depression, history of cognitive dysfunction, body mass index, and frailty according to the Clinical Frailty Scale[17]
- History of symptomatic and documented SARS-CoV-2 infection prior to the studied COVID-19 episode
- COVID-19 vaccination status up to the date of the studied SARS-CoV-2 infection episode
- Severity of the studied COVID-19 episode according to the worst level of the WHO Clinical Severity Scale (1- Home treatment with no limitations on daily living activities; 2- Home treatment with limitations on daily living activities; 3- Hospitalized treatment without the need for oxygen therapy; 4- Hospitalized treatment with the need for oxygen therapy via a mask or low-flow nasal cannula; 5 - Hospitalized treatment with the need for oxygen therapy via a high-flow nasal cannula or non-invasive ventilation; 6- Hospitalized treatment requiring invasive mechanical ventilation)[18]
- Treatments used during the acute phase of COVID-19, including antivirals (molnupiravir, nirmatrelvir/ritonavir, remdesivir), corticosteroids (dexamethasone, prednisone, prednisolone, methylprednisolone, and hydrocortisone), immunomodulators (tocilizumab, baricitinib), and monoclonal antibodies against SARS-CoV-2 (casirivimab/imdevimab, bamlanivimab/etesevimab, regdanvimab, and sotrovimab).

Exposure variable for comparison of cognition domains between participants with and without post-COVID-19 cognitive dysfunction (nested case-control study)

- Cases: Cognitive dysfunction according to the Brazilian version of the modified Telephone Interview for Cognitive Status (TICS-m; total score ≤ 21), [19]
- Controls: absence of cognitive dysfunction according to the Brazilian version of the TICS-m (total score > 21) paired with cases by age group, sex, education level and time since COVID-19 diagnosis.

Outcomes

Primary outcome

The primary outcome is the health-related quality of life utility score assessed through the Brazilian version of the EuroQol 5-Dimensions and 3-Levels scale (EQ-5D-3L).[20] The EQ-5D-3L comprises a descriptive system with five dimensions of quality of life (mobility, self-care, usual activities, pain/discomfort, and anxiety/depression). The utility score in the Brazilian population ranges from -0.17 to 1.0, with higher scores indicating better quality of life.[21] The clinically significant minimum difference varies from 0.03 to 0.52.[22]

Secondary outcomes

The secondary outcome measures include the variables described below:

- Degree of instrumental activities of daily living (use of the telephone, mobility, shopping, responsibility for one's medications, finances, and ability to handle finances) measured by the Brazilian version of the Lawton & Brody Instrumental Activities of Daily Living Scale[23]
- Degree of functional physical capacity measured by the Brazilian version of the Barthel Index[24]
- Degree of cognitive function measured by the Brazilian version of the Telephone Interview for Cognitive Status (TICS-m)[19]
- Symptoms of anxiety and depression measured by the Brazilian version of the Hospital Anxiety and Depression Scale (HADS)[25]
- Symptoms of post-traumatic stress measured by the Brazilian version of the Impact of Event Scale - 6 items (IES-6)[26,27]
- Return to work
- Return to studies

- Utilization of health resources (elective medical appointments, elective appointments with non-medical health professionals, urgent or emergency care, hospitalizations reported by the participant)

Secondary outcomes assessed in participants selected for the nested case-control study

- Degree of cognitive function measured by the Brazilian version of the Montreal Cognitive Assessment by Telephone (tMoCA)[28]
- Degree of memory function measured by the Brazilian version of the Rey Auditory Verbal Learning Test[29]
- Degree of language function measured by the Brazilian version of the Montreal-Toulouse Language Assessment Battery - Oral Naming Subtest[30]
- Degree of intelligence function measured by the Brazilian version of the Word Accentuation Test [31]
- Degree of attention function measured by the Brazilian version of the Neupsilin Reverse Counting Test - Reverse Counting Subtest[32]
- Degree of working memory function measured by the Digit Span[33]
- Degree of executive disfunction measured by the Barkley Deficits in Executive Functioning Scale (BDFES)[34]

Study procedures

Participant screening and informed consent process

Individuals expressing interest through the study's webpage will initially be contacted via phone or text message (according to their preference indicated in the registration), considering the stratification of the sample according to region of residence and age group. Potentially eligible individuals contacted by phone will be invited to voluntarily participate in the study through the conduction of the consent process and consent registration in an electronic informed consent form (e-ICF). Individuals contacted in person, will also register on the study's webpage if expressing interest in participating. If they wish, they can proceed to the consent process, which will be conducted in person by the researcher who provided access to the e-ICF. Interviews will always be conducted by phone, and the interview can be scheduled at the time of the in-person contact. The consent process is detailed in the ethics and good clinical practices section of this protocol. A participant who consents to participate

will proceed to the confirmation of eligibility criteria. If the participant has not taken a PCR or antigen test to confirm the diagnosis of COVID-19, participation will be terminated, and no additional information will be requested. Contact with prospective research participants will be conducted by researchers who are properly trained in good clinical practices, maintaining anonymity and confidentiality of data, and promoting the best conditions of comfort and safety for the prospective participant and, in the case of express consent, to the study participant. The details of this process are available in Appendix 1 (Recruitment and Dissemination Plan).

Data collection

Participants included in the cohort study will be assessed in a single interview conducted by a trained researcher with expertise in good clinical practices. The interview will take place within a 0–15-day window after the participant signs the ICF. Participants selected for the case-control study will participate in an additional interview to assess cognitive domains, provided they initially consent to be contacted and after signing a specific ICF for this sub-study. All interviews will be conducted remotely (phone call or video call), structured, and centralized by researchers from INOVA Research who are trained in data collection in this modality and qualified to administer the specific tests of the case-control study. The estimated duration of the cohort study interview is 50 minutes, and the case-control study interview is estimated to last 45 minutes. Table 2 summarizes the variables that will be collected in the study.

Table 2. Data collection plan

**Data**	**Cohort Study**	**Case-control Study**
Date of birth	x	x
Sex	x	x
Education	x	x
Smoking	x	x
Alcohol consumption	x	x
Charlson Comorbidity Index	x	x
History of anxiety or depression	x	x

History of cognitive dysfunction	x	x
Body mass index	x	x
Clinical Frailty Scale	x	x
Information about previous COVID-19	x	x
Vaccination status for COVID-19	x	x
Presence and intensity of long-COVID-19 symptoms (dyspnea, cough, change in smell, change in taste, fatigue, post-exertional malaise, headache, paresthesia, difficulty concentrating, memory difficulty, insomnia, anxiety, depression, joint pain, muscle pain, and others)	x	x
EQ5D-3L – How it was before the index COVID-19 episode (1 month before) and how it was now	x	x
Instrumental activities of daily living (Lawton and Brody scale) - How it was before the index COVID-19 episode (1 month before) and how it was now	x	x
Functional physical capacity (modified Barthel index) - How it was before the index COVID-19 episode (1 month before) and how it was now	x	x
Cognitive function (TICS-m)	x	x
Anxiety and depression symptoms (HADS)	x	x
Symptoms of post-traumatic stress disorder (IES-6)	x	x
Information about returning to work	x	x
Information about returning to study	x	x
Information on the use of health resources (elective medical consultations, elective consultations with non-medical health professionals, urgent or emergency care, hospitalizations)	x	x
Degree of cognitive function in multiple domains (t-MOCA, Rey Auditory Verbal Learning Test, Montreal-Toulouse Language Assessment Battery - Oral Naming Subtest, Word Accentuation Test, Neupsilin Reverse Counting Test - Reverse Counting Subtest, Digit Span Test, and BDEFS)	N/ A	x

BDEFS, Barkley Deficits in Executive Functioning Scale; EQ-5D-3L, 5-dimensional, 3-level EuroQol scale; HADS, Hospital Anxiety and Depression Symptom Scale; IES-R, Revised Event Impact Scale; N/A, not applicable; TICS-M, modified cognitive status assessment telephone interview; tMOCA, Montreal Telephone Cognitive Assessment.

Data management

Data collection will be conducted using electronic clinical forms filled out by the researcher conducting the telephone interview based on the participant's reported data. The

tool to be used for data collection and management is the Otus platform (https://site.otus-solutions.com.br/). Access to the data platform will be granted to each team member through personal and non-transferable usernames and passwords, following proper delegation within the study by the responsible researcher. Platform users (research team) will have specific permissions related to their role and delegation in the study.

Data integrity

Several procedures will be employed to ensure data security and quality:

- All researchers will undergo training before the study begins on good clinical practices and study procedures, including data collection.
- Processes related to data management will comply with the Brazilian General Data Protection Law (LGPD; Law No. 13,709, of August 14, 2018).
- Automated daily backups of the database will be performed.
- Data extraction for statistical software will be automated, with data anonymization for data consistency checks, monitoring actions, derivation of variables, and statistical analyses.
- Data cleaning for identifying inconsistencies will be conducted periodically, and researchers will be notified of inconsistencies for correction.
- Remote interviews will be recorded and audited for consistency checking and data adjudication. Audio files will be stored in an anonymized manner on a server with the same security system as the database described above
- Principal researchers will review detailed monthly reports on screening, inclusion, follow-up, data consistencies, and completeness, taking immediate actions to address any issues.
- Statistical techniques to identify fraud will be employed throughout the study.

Sample size

Considering an incidence of post-COVID-19 condition of 20%[4], a sample size of 1,540 participants with a history of COVID-19 is estimated to allow the detection of a difference greater than or equal to 0.05 in EuroQol 5-Dimensions and 3-Levels (EQ-5D-3L) quality of life utility scores (within the range of minimally clinically significant difference)[22]

between patients with post-COVID-19 condition and participants without post-COVID-19 condition, with a power of 80%, a two-tailed alpha of 0.05, and a standard deviation of the utility score of 0.28. To account for potential uncertainties related to post-COVID-19 quality of life parameters and to address the loss of power due to the need for covariate adjustment, the sample was inflated by 10%. Thus, the present cohort study intended to recruit 1,694 participants.

For the nested case-control study, a sample size of 123 participants (63 cases of post-COVID-19 cognitive dysfunction and 63 controls without post-COVID-19 cognitive dysfunction) will allow the detection of a mean difference with an effect size of 0.05 (considered moderate according to Cohen) in cognitive function scores.

Statistical analysis

Continuous variables will be expressed as mean and standard deviation or as median and interquartile range, while categorical variables will be expressed as absolute and relative frequencies. Data normality will be assessed by histogram inspection.

The comparison of EQ-5D-3L utility scores (primary outcome) between patients with and without post-COVID-19 condition will be performed using adjusted generalized linear models for age, gender, comorbidities, frailty, and region of residence (North, Northeast, Southwest, Midwest, and South). Adjusted results for EQ-5D-3L utility scores and questionnaire domains (mobility, self-care, usual activities, pain/discomfort, and anxiety/depression) will be summarized for each comparison group using central tendency and dispersion measures, along with the mean or median difference as a measure of effect size. The comparison of secondary outcomes between patients with and without post-COVID-19 condition will be performed using the same model as the primary outcome. The effect measure used will be absolute difference (for categorical outcomes) and mean or median difference (for continuous outcomes).

The assessment of factors associated with post-COVID-19 condition will be conducted using generalized linear models considering a Poisson distribution with robust variance. Variables with a p-value < 0.20 will be included in the multivariable model using a forward procedure. The association result of variables with post-COVID-19 condition will be described through relative risk.

The comparison between cognition domain test scores among cases of post-COVID-19 cognitive dysfunction and controls without post-COVID-19 cognitive dysfunction will be performed using generalized linear models. The effect measure used will be mean or median difference.

A statistical significance level of 0.05 will be considered for all comparisons. 95% confidence intervals will be described for all effect measure analyses, without adjustment for multiplicity. Analyses will be conducted using R software, with the version to be detailed at the time of analysis.[35]

ETHICS AND GOOD CLINICAL PRACTICES

The research has been planned and will be conducted in accordance with Resolution 466 of December 12, 2012, from the National Health Council [36] and the International Council for Harmonisation's Good Clinical Practice Guidelines, Amendment 6 – Revision 2 (ICH),[37]], in addition to the standards recommended by the Brazilian General Data Protection Law (LGPD). The research will only begin after full ethical committee approval of the protocol and related documents. This research also follows the guidelines for procedures in research with any stage in a virtual environment, as described in Circular Letter No. 1/2021 from the Brazilian National Research Ethics Commission (CONEP) dated March 3, 2021.

Procedures for participant consent

Following the Guidelines and Regulatory Standards for Research Involving Human Beings, established by Resolution 466/12 of the National Health Council, an ICF will be presented to prospective research participants at the time of invitation to participate in the study. The ICF will provide, in accessible and clear language, information about the objectives, methods, process of data collection and recording of the study, and also: explanation of the possible discomforts and risks arising from participation and their mitigating measures; explanation of the expected benefits of this participation; guarantee of full freedom for the participant to refuse to participate or withdraw their consent at any stage of the research, without any penalty; guarantee of maintaining the confidentiality and privacy of research participants during all phases of the research; guarantee that the research participant will receive a copy of the ICF; explanation of the guarantee of reimbursement and

how the expenses incurred by the research participants and arising from it will be covered and explanation of the guarantee of compensation in the event of any damages arising from the research.

Considering the diversity of generations and their preferences, facilities, or limitations regarding the use of technologies, the consent process may occur independently or assisted. In independent consent, the digital ICF (e-ICF) will be made available to the individual interested in participating in the study, at the time of the initial contact, after confirmation of the data provided in the interest registration. The e-ICF will be made available via an individual link, by email or text message, according to the prospective participant's preference. The prospective participant will read the term and record their consent electronically. In the assisted process, it will occur if the participant states that they want assistance or additional clarifications. In these cases, the individual will have the option of talking to one of the researchers by text message or phone (whatever the individual prefers), to clarify any doubts or the electronic registration process of consent. If the consent process is carried out in person, the participant will receive the link to the e-ICF in their email and can clarify any doubts in person with the researcher.

The prospective study participant will have sufficient time to read the e-ICF and the opportunity to ask questions for the researcher. After the explanations and before any study procedures, consent will be recorded through a registration of acceptance in the e-ICF. After registration of consent, a copy of the e-ICF will be made immediately available to the participant, via their email address or text message, according to the participant's preference, and a printed copy may be sent by mail later, if the participant wishes. The copy of the e-ICF will include the name of the researcher who made the e-ICF available and conducted the consent process, as well as the date and time of the participant's consent registration.

Participants who consent to be contacted for participation in the nested case-control study, if selected, will only participate in this sub-study if they voluntarily consent after reading a specific e-ICF for this study, following the same consent registration procedures.

Potential risks and benefits

The risks to the study participant are minimal, as the study is observational and involves only interview activities for data collection. Regarding data collection, even though

the team is trained in the standards set forth by the Brazilian General Data Protection Law, there is a risk of breach of confidentiality and data privacy, which are mitigated as described in the Data Management and Confidentiality items in this protocol. Additionally, there are risks related to discomfort when answering questions related to health. In these cases, the participant may choose not to answer. The time required to conduct the interview can also be considered a discomfort.

Although this study does not have an immediate benefit for the participants, it may bring long-term contributions, since the knowledge obtained through its execution has the potential to direct national policies and efforts for the prevention, treatment, and recovery of patients affected by COVID-19.

At the end of the interview, all participants will receive a report with the individual results of the tests answered (Appendix 2 – Template of report with individual results) - and, for those participants whose test scores are indicative of the presence of symptoms, they will be advised to present this document to their attending physician. It is important to reinforce that the applied tests do not define a diagnosis. The tests can signal symptoms and can be used in a complementary way to assist a clinical evaluation that may be carried out by a medical professional in an eventual consultation carried out by the participant. As knowing this information is a right of the participant, therefore, it is not treated as a benefit in the ICF.

Confidentiality

All personal information of the participant will be kept confidential, and only the study team will have access to prevent breaches of confidentiality. At no circumstances will the participant's name or any information about their health be provided to anyone outside the study team who performs an activity that requires access to this information. The information will be confidential and used solely for the purposes of this research. All appropriate measures to prevent the breach of the participant's identity and confidentiality will be executed. These include access to study documents only by research team members and storage of electronic data in a database with secure access via individual user and password for researchers and other members of the research team only. The results of the study will be disseminated in an aggregated form, for academic and scientific purposes, without the identification of any data that reveals the identity of the participants.

STUDY ORGANIZATION

Study coordination

The study will be coordinated by INOVA Research, a department of INOVA MEDICAL - HEALTH SERVICES AND MANAGEMENT LTD, responsible for the operationalization of clinical studies. The coordinating center is responsible for providing the support necessary for the conduct of the study, including ethical/regulatory procedures; training, guidance, and support for delegated researchers to ensure adherence to the research protocol and good clinical practices; centralized remote follow-up of research participants; monitoring and data management; data analysis; and dissemination of research results. The coordinating center team has training and experience in clinical research, statistics, and bioethics.

Steering Committee

The Steering Committee is responsible for overseeing the execution of the study, ensuring ethical integrity and high standards of methodological quality. The specific responsibilities of the steering committee include: 1) monitoring the progress of the study according to predefined objectives; 2) supervising the quality of the methods employed in the research; 3) reviewing, at regular intervals, information from the scientific literature that may require modifications to the study procedures; 4) ensuring that regulatory bodies related to clinical research are notified appropriately when necessary; 5) implementing the study dissemination policies. The members of the Steering Committee are trained researchers with experience in the design and conduct of clinical studies, as well as in the areas of epidemiology and clinical research.

Sponsor

INOVA MEDICAL - HEALTH SERVICES AND MANAGEMENT LTD has the responsibility of a sponsoring institution as described in Brazilian National Health Surveillance Agency (ANVISA) rule RDC 09/2015[38] and the Guidelines for Good Clinical Practice, E6 (R2) of the ICH.[37]

Funding

This study is funded by MERCK SHARP DOHME FARMACÊUTICA LTDA (MSD), registered under the National Registry of Legal Entities under the number 03.560.974/0001-18, through the Merck Investigator Studies Program (MISP#101021) research grant.

DISSEMINATION POLICY

Dissemination plan

The principal investigators expect to disseminate the results of this study through presentations at scientific conferences and publication in peer-reviewed scientific journals. The decision regarding the publication of the study data, as well as the target journal, will be the responsibility of the study steering committee.

The results will also be disseminated directly to the participants, with a summary of the results in Portuguese, and in easy-to-understand language, sent to the participants via email. In addition, this information will be available on the website used for recruitment (www.covidlonga.com.br). These results will be released only after the completion of the study.

Authorship Policy

To be credited as an author of any article(s) resulting from this study, the applicant must meet all 4 criteria recommended by the International Committee of Medical Journal Editors (ICMJE) [39] listed below:

Criterion 1: Make substantial contributions to at least one of the following: conception and design, acquisition of data, analysis and interpretation of data;

Criterion 2: Be involved in drafting the manuscript or revising it critically for intellectual content;

Criterion 3: Approve the final version of the manuscript to be published;

Criterion 4: Agree to be accountable for all aspects of the work in ensuring that questions related to the accuracy or integrity of any part of the work are appropriately investigated and resolved

Data sharing policy

Data sharing requests will be subject to evaluation by the study Steering Committee.

REFERENCES

1. Johns Hopkins University & Medicine (2022) Coronavirus Resource Center. https://coronavirus.jhu.edu/map.html.
2. Crook H, Raza S, Nowell J, et al. Long covid-mechanisms, risk factors, and management. BMJ 2021;374:n1648.
3. Michelen M, Manoharan L, Elkheir N, et al. Characterising long COVID: a living systematic review. BMJ Glob Health 2021;6(9):e005427.
4. World Health Organization. A clinical case definition of post COVID-19 condition by a Delphi consensus. 2021. WHO/2019-nCoV/Post_COVID-19_condition/Clinical_case_definition/2021.1
5. Global Burden of Disease Long COVID Collaborators. Estimated Global Proportions of Individuals With Persistent Fatigue, Cognitive, and Respiratory Symptom Clusters Following Symptomatic COVID-19 in 2020 and 2021. JAMA 2022;328(16):1604-1615.
6. O'Mahoney LL, Routen A, Gillies C, et al. The prevalence and long-term health effects of Long Covid among hospitalised and non-hospitalised populations: A systematic review and meta-analysis. EClinicalMedicine 2022;55:101762.
7. Chen C, Haupert SR, Zimmermann L, et al. Global Prevalence of Post-Coronavirus Disease 2019 (COVID-19) Condition or Long COVID: A Meta-Analysis and Systematic Review. J Infect Dis 2022;226(9):1593-1607.
8. Ballering AV, van Zon SKR, Olde Hartman TC, et al. Persistence of somatic symptoms after COVID-19 in the Netherlands: an observational cohort study. Lancet 2022;400(10350):452-461
9. Munblit D, TR Nicholson, DM Needham, et al. Studying the post-COVID-19 condition: research challenges, strategies, and importance of Core Outcome Set development. BMC Med 2022;20:50.
10. Tsuzuki S, Miyazato Y, Terada M, et al. Impact of long-COVID on health-related quality of life in Japanese COVID-19 patients. Health Qual Life Outcomes 2022;20(1):125.
11. Logue JK, Franko NM, McCulloch DJ, et al. Sequelae in Adults at 6 Months After COVID-19 Infection. JAMA Netw Open 2021;4(2):e210830.

12. Rosa RG, Cavalcanti AB, Azevedo LCP, et al. Association between acute disease severity and one-year quality of life among post-hospitalisation COVID-19 patients: Coalition VII prospective cohort study. Intensive Care Med 2023:1–12.
13. Brightling CE, Evans RA. Long COVID: which symptoms can be attributed to SARS-CoV-2 infection? Lancet 2022;400(10350):411-413.
14. Kovelis D, Segretti NO, Probst VS, Lareau SC, Brunetto AF, Pitta F. Validation of the Modified Pulmonary Functional Status and Dyspnea Questionnaire and the Medical Research Council scale for use in Brazilian patients with chronic obstructive pulmonary disease. J Bras Pneumol. 2008 Dec;34(12):1008-18.
15. Valderramas S, Camelier AA, Silva SA, et al. Reliability of the Brazilian Portuguese version of the fatigue severity scale and its correlation with pulmonary function, dyspnea, and functional capacity in patients with COPD. J Bras Pneumol. 2013;39(4):427-33.
16. Charlson ME, Pompei P, Ales KL, MacKenzie CR. A new method of classifying prognostic comorbidity in longitudinal studies: development and validation. J Chronic Dis 1987;40(5):373-383.
17. Church S, Rogers E, Rockwood K, Theou O. A scoping review of the Clinical Frailty Scale. BMC Geriatr 2020;20(1):393.
18. Working Group on the Clinical Characterisation and Management of COVID-19 infection. A minimal common outcome measure set for COVID-19 clinical research. Lancet Infect Dis 2020;20:e192-e7.
19. Cook SE, Marsiske M. The Use of the Modified Telephone Interview for Cognitive Status (TICS-M) in the Detection of Amnestic Mild Cognitive Impairment. J Geriatr Psychiatry Neurol 2009; 22(2): 103–109.
20. Santos M, MA Cintra, AL Monteiro, et al. Brazilian Valuation of EQ-5D-3L Health States: results from a saturation study. Med Decis Making 2016;36:253-63.
21. Santos M, AL Monteiro, B Santos. EQ-5D Brazilian population norms. Health Qual Life Outcomes 2021;19:162.
22. Coretti S, M Ruggeri, P McNamee. The minimum clinically important difference for EQ-5D index: a critical review. Expert Rev Pharmacoecon Outcomes Res 2014;14:221-33.
23. Santos RL, Virtuoso Jr JS. Reliability of the Brazilian version of the Scale of Instrumental Activities of Daily Living. Rev Bras Prom Saúde. 2008;21(4):290-6.

24. Minosso JS, Amendola F, Alvarenga MR, Oliveira MA. Validation of the Barthel Index in elderly patients attended in outpatient clinics, in Brazil. Acta Paul Enferm 2010;23(2):218–223.
25. Zigmond AS, Snaith RP. The hospital anxiety and depression scale. Acta Psychiatr Scand 1983;67(6):361-70.
26. Caiuby AV, SS Lacerda, MI Quintana, TS Torii, SB Andreoli. Cross-cultural adaptation of the Brazilian version of the Impact of Events Scale-Revised (IES-R). Cad Saude Publica 2012;28:597-603.
27. Thoresen S, Tambs K, Hussain A, Heir T, Johansen VA, Bisson JI. Brief measure of posttraumatic stress reactions: impact of event scale-6. Soc Psychiatry Psychiatr Epidemiol. 2010;45:405–12.
28. Pendlebury ST, Welch SJ, Cuthbertson FC, et al. Telephone assessment of cognition after transient ischemic attack and stroke: modified telephone interview of cognitive status and telephone Montreal Cognitive Assessment versus face-to-face Montreal Cognitive Assessment and neuropsychological battery. Stroke 2013;44(1):227-9.
29. Rosemberg SJ, Rayan JJ, Prifitera. Rey Auditory-Verbal Learning Test performance of patients with and without memory impairment. J Clin Psychol 1984;40(3):785-7.
30. Altmann RF, Ortiz KZ, Benfica TR, et al. Brief Montreal-Toulouse Language Assessment Battery: adaptation and content validity. Psicol Reflex Crit 2020;33(1):18.
31. Gil G, Magaldi RM, Busse AL, et al. Development of a word accentuation test for predicting cognitive performance in Portuguese-speaking populations. Arq Neuropsiquiatr 2019;77(8):560-567.
32. Fonseca R, Salles JF, Parente MAMP. Development and content validity of the brazilian brief neuropsychological assessment battery Neupsilin. Psychol Neurosci. 2008;1:55–62.
33. Zimmermann, N., Cardoso, C.O., Trentini, C.M., Grassi-Oliveira, R., Fonseca, R.P. (2015). Brazilian preliminary norms and investigation of age and education effects on the Modified Wisconsin Card Sorting Test, Stroop Color and Word test and Digit Span test in adults. Dementia & Neuropsychologia, 9(2), 120-127.
34. Godoy, V.P., Mattos, P., & Malloy-Diniz, L.F. (2011). Escala de Avaliação de Disfunções Executivas de Barkley – BDEFS. São Paulo, Brasil: Hogrefe.

35. R Development Core Team. R: A language and environment for statistical computing. Vienna: R Foundation for Statistical Computing; 2016. https://www.r-project.org
36. Conselho Nacional de Saúde (Brasil). Resolução n. 466, de 12 de dezembro de 2012. Brasília, DF, 2012.
37. International conference on harmonisation of technical requirements for registration of pharmaceuticals for human use. ICH Harmonised Tripartite Guideline. Guideline for good clinical practice. E6(R1). 2016.
38. Agência Nacional de Vigilância Sanitária (ANVISA). Resolução da diretoria colegiada - rdc nº 9, de 20 de fevereiro de 2015. Brasília, DF, 2015. http://antigo.anvisa.gov.br/documents/10181/3503972/%282%29RDC_09_2015_COMP.pdf/ee294d51-055f-4244-8e1a-b62f0c8e4f88
39. International Committee of Medical Journal Editors. Defining the role of authors and contributors. http://www.icmje.org/recommendations/browse/roles-and-responsibilities/defining-the-role-of-authors-and-contributors.html

Appendix_1_Recruitment_and_Dissemination_Plan_v3_20_apr_2024

Incidence, associated factors and impact of the post-COVID-19 condition in Brazil during the Omicron phase: an observational study

Recruitment and Dissemination Plan

Recruitment and informed consent procedures

This research follows the guidelines for procedures in research with any stage in a virtual environment, as described in Circular Letter No. 1/2021 from the Brazilian National Research Ethics Commission (CONEP) dated March 3, 2021, since all its stages may be developed in a virtual environment. Participant recruitment will be conducted electronically, through a website developed exclusively for the study's dissemination, where interested individuals will fill out a form (registration) with basic information so that the research team can initiate contact. Only people who have completed this registration will be contacted, as completing the registration indicates an expression of interest in the study and authorization for the person to be contacted by the team. The site is available for access at www.covidlonga.com.br, however, the function of receiving information through will be enabled only after approval to start recruitment by the Ethics Committee.

The website was built using WordPress and is secured with an SSL certificate, which verifies the website's identity and encrypts data transmitted between the website and users. This is the same type of security used for online credit card transactions. The website is hosted on a dedicated server, meaning it is the sole occupant of that server and all data related to the website is stored there exclusively. This is a significant security measure, as most websites share servers with other organizations, increasing the risk of data breaches. Furthermore, the server is located within the country, and the hosting company, www.hostoo.com.br, is a domestic company. As a result, all entities involved in this process are subject to Brazilian General Data Protection Law (LGPD, Law No. 13.709/2018).

The website provides an overview of the study and what the participation entails, along with the research team's contact details (email and phone number). It is also informed that this is a registration of interest in the research, from which the team will contact the prospective participant to explain participation in the research and that one will only be a participant if consented to participate voluntarily and by recording this decision in the Informed Consent Form (ICF). The data to be collected in the website registry form will be:

Full name (as per identification document);

Pronoun;

Age in years;

Sex assigned at birth (with the possibility of not informing it);

State where you live;

City where you live;

Email (it is recommended that it be accessed frequently, at least once a week);

Phone number with code area;

How do you prefer to be contacted by the research team (by text message app or by phone call/voice call);

Preferred shift for contact;

Additional comments (for example, time you prefer the contact by the research team), optional field;

Request for confirmation of previous COVID-19 infection after January 1, 2022;

Request for confirmation that you agree to be contacted by the research team.

How did you hear about the study (optional field)"

At the end of the website registration, the following information will be displayed:

It is expected that 1,694 participants will participate in this research. Contacts from the research team will occur in the order of registration, based on the information provided and also considering the proportional distribution by Brazilian States and age of the participants.

This registration also does not guarantee your inclusion in the study, which will only be effective from the contact by a researcher and your voluntary consent to participate.

Data Policy

All data will be treated in accordance with the Brazilian General Data Protection Law (LGPD - Law No. 13.709, of August 14, 2018). The data collected here will be used strictly by the research team duly registered with Inova Medical, sponsor of the research, and only for the purpose of contacting for participation in the research.

Only trained researchers involved in the study will have access to the data imputed in the website, and they are strictly bound by confidentiality agreements. Registration data will be periodically deleted (every 2 weeks), except for the following information, which will be retained for record-keeping and auditing purposes: participant age, state of residence, sex, registration date, whether or not the participant was included in the study, and the reason for inclusion or exclusion (e.g., unable to contact after 10 attempts, declined to participate, technical issues with the electronic ICF (e-ICF), hearing or speech impairments, quota filled, other [specify]). Additionally, the website will be used to collect statistics on page visits (daily) to monitor recruitment progress.

The initial contact, invitation to participate in the study, and the process of obtaining informed consent can also be conducted in person by trained recruiters delegated by the principal investigator. In such cases, the interested individual will also be required to register their interest on the study's electronic page.

Once an individual has registered on the study website, the research team will contact them. The team will introduce themselves, verify the provided information (sex, age, state and city of residence, date of the COVID-19 episode, and how the diagnosis was made). If this data aligns with the study's eligibility criteria, the prospective participant will be asked to confirm their email address and full name. Subsequently, the consent process will commence. The participant will be asked to read the e-ICF and will be offered assistance in reading the e-ICF if needed. The researcher will remain available to answer any questions, or if the prospective participant prefers, a follow-up call can be scheduled to address any queries. Alternatively, the researcher can be available to answer questions via text message. Study processes will only

begin after the participant records the decision in the e-ICF and confirms receipt of an email with the complete e-ICF with date and time of consent, as well as the name of the researcher who provided the e-ICF and conducted the process.. A printed copy of the ICF, with the date and time of consent, and the name of the researcher who provided the ICF and conducted the informed consent process, can also be sent by mail if the participant wishes.

In all telephone or video call contacts, it will be confirmed whether the potential participant is in a physically safe place to proceed with the call. Procedures will not be conducted if the potential participant is, for example, walking, driving, or in any other unsafe situation. If the call quality is deemed inadequate by the researcher, the contact will be terminated and rescheduled.

Any data collected from a prospective participant who were contacted but did not consent to participate in the study will be deleted.

For in-person recruitment, after the study is explained, the e-ICF will be provided through email. The participant will then read the and electronically record their consent or refusal to participate, following the same process as the remote recruitment. In this case, the individual may clarify any doubts with the recruiter or, if preferred, complete the e-ICF at a more suitable time, with or without assistance. In-person recruitment will be conducted through home visits, avoiding any approach in public places to ensure the privacy of invited prospective participants.

Dissemination of the study's website

The study will be advertised through email, social media (Facebook and Instagram) ads, WhatsApp, and Google AdSense. All promotional materials, in addition to those presented here, will be submitted to the Ethics Committee for approval.

Examples of possible dissemination materials:

1) E-mails to medical societies, hospitals, universities by using public contacts:

Sender: contato@covidlonga.com.br

Issue: Long COVID Brazil Study

Message:

Dear xxx,

With the aim of assessing the long-term impact of COVID-19 infection, we are recruiting adults who had the disease after January 1, 2022, confirmed by a test. A total of 1,694 adults from all Brazilian regions will be interviewed by phone or video call. The estimated participation time is approximately 1 hour. The study and this dissemination were approved by the Ethics Committee of Hospital Moinhos de Vento under opinion 6.088.948 of April 18, 2023.

The form for expressing interest in participating and additional information about the study are available on the website: www.covidlonga.com.br

If you know someone who may be interested in participating in the study, we would appreciate it if you could forward this message or share the website www.covidlonga.com.br. If you have any questions, please reply to this email or contact us via WhatsApp (51) 9819-2778.

Thank you for your cooperation.

Attached files: none.

2) Examples for social media dissemination:

Message #1:

Help us to build knowledge about the consequences of COVID-19.

Participate in the Covid Longa Brazil Study.

Learn more: www.covidlonga.com.br

Message #2:

Did you have COVID-19 in 2022 and are you 18 or older?

Join the Covid Longa Brazil Study.

Learn more: www.covidlonga.com.br

Message #3:

Did you or someone you know have COVID-19 in 2022?

Learn about the Covid Longa Brazil Study.

Learn more: www.covidlonga.com.br

Message #4:

Did you have COVID-19 in 2022 and are you 18 or older?

Donate 1 hour of your time and join the Covid Longa Brazil Study.

Learn more: www.covidlonga.com.br

Message #5:

Did you have COVID-19 in 2022? Join our research and help us understand the long-term effects of the disease. Your experience is essential!

Learn more: www.covidlonga.com.br

Message #6:

Experienced COVID-19 in 2022? Your contribution to our research can help improve the understanding and treatment of the disease's long-term effects. Join us!

Learn more: www.covidlonga.com.br

Message #7:

If you are an adult who contracted COVID-19 in 2022, we are seeking participants for a study on Long COVID. Participate and help us understand the long-term effects of this disease!

Learn more: www.covidlonga.com.br

Message #8:

If you had COVID-19 in 2022, we invite you to participate in a research study on the long-term effects of the disease. Help us advance science and public health!

Learn more: www.covidlonga.com.br

3) Exemple for WhatsApp messages:

The Covid Longa Brazil study will interview 1,694 adults who had COVID-19 starting January 1st, 2022.

If you or a family member had the disease, you can contribute to a better understanding of the long-term consequences of COVID-19.

Learn more: www.covidlonga.com.br

Messaging App

Given that WhatsApp is present on over 99% of smartphones in Brazil (source: Mobile Time/Opinion Box Messaging Panorama in Brazil, August 2022) and offers security mechanisms for sensitive data, all communication with participants that doesn't occur directly through phone calls or email will be conducted solely through WhatsApp. For this purpose, the study has a WhatsApp Business account and employs all security levels recommended by Meta, such as two-factor authentication and internal authorization controls. Regarding internal authorization, only researchers tasked with participant contact will be authorized to use the account, and they will be expressly committed to data confidentiality in a study document. Researchers not assigned to participant contact will not have access to the account. To enable

this control, the WhatsApp account used to promote the study will be different from the one used for direct communication with participants. Finally, all communication with participants is conducted directly by a trained researcher who will manually use standardized texts to ensure information reproducibility. The study will not use automated communication tools such as chatbots.

Participant's reimbursements

If the participant incurs any costs for internet data packages, calls, or text messages (SMS), they will be reimbursed. Reimbursements will be made via PIX (Brazil's instant payment system). The following values will be considered as the standard reimbursement if the potential participant reports that the costs are not covered by their current phone or internet plan:

SMS sent: R$ 0.40

Outgoing call minute (mobile network): R$ 1.00

Outgoing call minute (landline): R$ 0.30

Incoming or outgoing call minute via internet (no video): R$ 0.10 per 10 minutes (considering R$ 9.00 as the cost of 1GB of data, with 1GB being consumed every 15 hours of calls)

Incoming or outgoing video call minute via internet: R$ 0.30 (considering R$ 9.00 as the cost of 1GB of data, with 1GB being consumed every 30 minutes of video calls)

Additional reimbursement values will be applied upon confirmation of expenses; reimbursement values up to R$ 50.00 will be made based on the presumption of the truthfulness of the potential participant's report, while for additional values, proof will be requested (for example, damage to the cell phone during study participation). Reimbursements will be applicable from the initial contact by the research team, regardless of participation in the study.

Monetary values will not be disclosed in advance to the participant to avoid inducing participation due to financial reimbursement. After participating in the study or substudy (or during recruitment in case of non-inclusion), the individual will be asked: "Did you incur any costs during this study that require reimbursement, such as phone and internet costs?" If the answer is yes, they will be asked what type of resource was used and the quantity will be

quantified according to the contact records. To receive the reimbursement, the participant must provide the PIX key in writing, being responsible for providing this information. If the participant does not use this resource for financial transactions, other means of reimbursement will be sought, such as bank deposit or providing a prepaid debit card.

Long Covid Brazil Study

Appendix_2_Template_individual_results_report_v2_31_jul_2023

Incidence, associated factors and impact of the post-COVID-19 condition in Brazil during the Omicron phase: an observational study

Date of telephone assessment: <date>

COVID-19 index episode date: <incluir data>

Researcher responsible for the assessment: <incluir nome do entrevistador>

Principal Investigator: Dr. Maicon Falavigna. CRM-RS 31.743.

We acknowledge your participation in the Long Covid Brazil Study. This report shares the results of the tests conducted during your telephone assessment. You will also receive an email with access to the general results of the COVID Longa Brazil Study when they are available; our expectation is that the results will be released in the first half of 2025.

It is important to clarify that the telephone assessment conducted and the data presented in this report do not replace a proper clinical evaluation by a physician consultation. Although the tests are validated questionnaires accepted both in clinical practice and in health research, they generally serve as symptom screening, and are not in themselves sufficient to define diagnoses or suggest specific medical decisions. Furthermore, these tests are not specific to COVID, so it is not possible to determine whether or not there was a direct relationship with the COVID episode.

The tests performed that have known information in the literature for a possible interpretation, as well as your results and interpretation of these results, are presented below:

HADS (Hospital Anxiety and Depression Scale)

It consists of a questionnaire designed to assess potential symptoms of anxiety and depression. Although initially developed to evaluate hospitalized individuals, it is currently widely used to assess individuals in an outpatient setting and by telephone. The HADS scale is divided into two subscales: HADS-A (to assess anxiety symptoms) and HADS-D (to assess depression symptoms). A score of 7 or higher on each of the scales indicates the presence of symptoms compatible with anxiety and depression.

Result:

HADS-Anxiety symptoms): Score <score>

Meaning:

<In case of score < 7: The score is within the normal range, meaning no symptoms suggestive of anxiety were detected.>

<In case of score ≥ 7: Your score on the questionnaire is higher than the cutoff point. However, this test is only for screening purposes and does not necessarily mean you have an anxiety problem. Please discuss these results with your doctor.>

HADS-D (depression): Score <result>

Meaning:

<In case of score < 7: The score is within the normal range, meaning no symptoms suggestive of depression were detected.>

<In case of score ≥ 7: Your score on the questionnaire is higher than the cutoff point. However, this test is only for screening purposes and does not necessarily mean you have a depression problem. Please discuss these results with your doctor.>

IES-6 (Impact of Events Scale-6 items)

Post-traumatic stress disorder (PTSD) is defined as a set of symptoms associated with a traumatic event. These events are quite diverse, ranging from accidents to natural disasters. Health-related events such as hospitalization, cancer diagnosis, and even COVID-19 can be triggers for PTSD. The Impact of Event Scale-6 (IES-6) has been referenced as the screening instrument for PTSD symptomatology. Scores above 10 suggest symptoms of PTSD.

Result:

IES-6: Score <score>

Meaning:

<In case of score < 10: The score is within the normal range, meaning no symptoms suggestive of PTSD were detected.>

<In case of score ≥ 10: Your score on the questionnaire is higher than the cutoff point. However, this test is only for screening purposes and does not necessarily mean you have a PTSD problem. Please discuss these results with your doctor.>

TICS-m (modified Telephone Interview for Cognitive Status)

The TICS-m is a clinical tool used to screen for cognitive impairment. It assesses cognitive functions like memory, attention, and reasoning. A score higher then 21 falls within the normal range.

Result:

TICS-m: Score <score>

Meaning:

<In case of score > 21: The score is within the normal range, meaning no cognitive dysfunction were detected. >

< In case of score ≤ 21: The result suggests a possible cognitive impairment. However, the test can be influenced by external factors such as fatigue, multitasking, or even poor phone connection quality. Therefore, it does not necessarily indicate a problem. We recommend discussing these results with your healthcare provider, who can conduct a more thorough evaluation to determine if there are any memory, attention, or reasoning issues affecting your quality of life.>

Barthel Index

It is a scale that assesses activities of daily living, aiming to evaluate the level of assistance needed for everyday tasks. The score ranges from 0 to 100. A score of 100 indicates no functional dependence, 91 to 99 indicates mild dependence, 61 to 90 indicates moderate dependence, 21 to 60 indicates severe dependence, and 0 to 20 indicates total functional dependence.

Result:

Score before COVID-19 index episode: <score>

Current score: <score>

Meaning:

<In case of current score = 100: Your score indicates no functional dependence.>

<In case of current score < 100: Your score indicates some functional dependence in the following daily activity domain: xxx.

<If current score is lesser than pre-Covid-19 score: Your functional dependence is higher than before the Covid-19 index episode. *We recommend discussing these results with your healthcare provider, who can conduct a more thorough evaluation to determine if there are potential alternatives that could enhance your quality of life.*>

Lawton & Brody Index

Similar to the Barthel Index, the Lawton & Brody scale assesses instrumental activities of daily living. However, while the Barthel primarily evaluates physical domains, the Activities of Daily Living (ADL) scale assesses instrumental activities of daily living, which include tasks such as using the telephone and managing finances. The score ranges from 0 (low functionality, dependent) to 8 (high functionality, independent).

Result:

Escore antes do episódio de COVID-19: <resultado do escore>

Escore atual: <resultado do escore>

Meaning:

<In case of current score = 8: Your score indicates no instrumental functional dependence.>

<In case of current score < 8: Your score indicates some instrumental functional dependence in the following activity domain: xxx.

<If current score is lesser than pre-Covid-19 score: Your instrumental functional dependence is higher than before the Covid-19 index episode. We recommend discussing these results with your healthcare provider, who can conduct a more thorough evaluation to determine if there are potential alternatives that could enhance your quality of life.>

Smoking (tobacco consumption)

According to the World Health Organization, tobacco is responsible for more than 8 million deaths annually, especially from lung complications, cardiovascular diseases (heart attacks and strokes), and cancer.

Result: According to your answers, you are currently a < smoker/non-smoker.>

<If non-smoker> Staying smoke-free is crucial for maintaining a healthy lifestyle. Keep up the good work!

<If smoker> It is important to discuss with your doctor alternative ways to reduce and preferably quit smoking. We understand that quitting smoking is not easy, but there are currently various treatments that can help you succeed. A doctor can help you develop the best strategy for you.

Alcohol consumption

According to the World Health Organization, alcohol consumption is responsible for approximately 3 million deaths annually. Alcohol is strongly associated with accidents, violence, psychiatric disorders, liver and digestive problems, and cardiovascular events.

Result: Based on your responses, your estimated weekly alcohol intake is xx grams (g).

According to the World Health Organization, there is no safe level of alcohol consumption, with the safest option being to avoid it altogether. US guidelines classify moderate drinking as up to 2 drinks (20 g of alcohol) per day for men and 1 drink (10 g of alcohol) per day for women; it's important to remember that certain individuals, such as pregnant women, breastfeeding mothers, and those with liver problems, should not drink. For comparison, a 350 ml can of beer, a 125 ml glass of red wine, and a 45 ml shot of spirits each contain approximately 16g of alcohol. This difference between the sexes is due to women having a lower amount of the enzyme responsible for metabolizing alcohol.

<If no-alcohol consumption: Your decision to avoid alcohol intake is the best choice for maintaining a healthy lifestyle.. Keep up the good work!>

<If alcohol consumption < 140 g/week for man or < 70 g/week for women>: Your alcohol intake can be classified as moderate. The decision of how much to drink is a personal one, but it's important to remember that alcohol consumption carries risks. The less you drink, the lower your risk of alcohol-related harm.

<If alcohol consumption > 140 g/week for man or > 70 g/week for women>: Your alcohol intake can be classified as high.

<Se consome >140 g/semana homem ou >70 g/semana mulher>: seu consumo de álcool é considerado elevado. It is important to reduce your consumption. We strongly recommend you to discuss this issue with your doctor.

Important note: The results presented in this report are from the COVID Longa Brasil study. They should be interpreted by your healthcare provider. The tests have limitations and cannot provide a definitive diagnosis. A comprehensive medical evaluation is necessary to understand the full picture of your health.

If you have any questions, don't hesitate to reach out to our study team. You can call us at (51) 9819-2778 or email us at contato@covidlonga.com.br.

Dr. Maicon Falavigna. CRM-RS 31.743 is responsible for this report.

Principal Investigator Signature

Report date: <data>

Cognitive bundle of tests conducted in the substudy for cognitive assessment will lead to a specific report developed by the neuropsychologist professional who conducted the tests and reviewed by the principal investigator. For each cognitive report the following note will be described: Your test results can be affected by things like tiredness, doing other tasks while taking the test, or even a poor phone connection. So, these results don't always mean there's a problem. We recommend talking to your doctor to get a better understanding of your results and to see if there are any concerns about your memory, attention, or other thinking skills.

INFORMED CONSENT FORM

Version 2, July 31, 2023

Title: Incidence, associated factors and impact of the post-COVID-19 condition in Brazil during the Omicron phase: an observational study – ***Long COVID Brazil Study***

Principal Investigator: Maicon Falavigna – CRM-RS 31.743

Sponsor: INOVA MEDICAL – HEALTH SERVICES AND MANAGEMENT

Site: Brazil

You are invited to voluntarily participate in the study entitled "Incidence, associated factors and impact of the post-COVID-19 condition in Brazil during the Omicron phase: an observational study", the ***Long COVID Brazil Study***. We noticed your interest in the study on our website (www.covidlonga.com.br). This study is being done across Brazil by Inova Medical and funded by Merck Sharp Dohme (MSD) LTD.

Before deciding to participate in this study, it is important that you understand why this study is being conducted, all the procedures involved, the potential benefits, risks, and discomforts that will be described and explained below. Participating or not participating in this study will not influence any type of healthcare you are currently receiving or will receive in the future.

At any time, before, during, or after the study, you may request information and clarification, refuse to participate, or withdraw from the study. In any of these cases, you will not suffer any negative consequences.

After reading this informed consent form and having your questions answered about the study, you will need to record at the end of this digital document whether or not you consent to participate in the study. Your voluntary consent to participate in the study will only occur if, at the end of reading this document, you check the box marked "YES, I consent to participate in the study voluntarily." If you do not wish to participate in the study, you should check the box marked "NO, I do not want to participate in the study." Regardless of your decision, this form will be sent to your email address, with a record of your decision, for your future reference. If you wish, we will send you by mail a printed copy of the document with your recorded decision.

This study aims to understand the post-COVID-19 effects on health-related quality of life in adults who have had COVID-19 during the dissemination of the Omicron variant in Brazil. This study will be conducted with 1,694 adults residing in any region of Brazil who have had COVID-19 with symptoms and confirmed by RT-PCR, rapid test, or antigen self-test (tests performed by collecting a nasal and/or throat swab) after January 1, 2022, but whose symptoms and test confirmation began at least 3 months prior to their participation in the study (i.e., 3 months prior to the current date, if you agree to participate).

If you agree to participate, the study procedures will involve verifying that: you are 18 years of age or older, reside in Brazil, tested positive for COVID-19 after January 1, 2022, and at least 3 months prior to the current date, and experienced symptoms when you had COVID-19

(e.g., fever, runny nose, cough, sore throat, among others). Confirmation of the RT-PCR test may be obtained by reading or sending the test result (report), by self-reporting the result of the test and corresponding date, or by accessing the *ConecteSUS* platform, performed by you. This procedure will take approximately 5 minutes. If you do not meet these criteria, you will not be able to continue in the study and no additional information will be requested. If you continue in the study, we will ask questions about your general living and health conditions before and after you had COVID-19, and questions related to specific tests that assess physical and mental health conditions. This procedure will be conducted by telephone or video call, will take approximately 50 minutes, and will be scheduled at a time that is most convenient for you, considering the recommendation that you be in a comfortable and safe place to answer the study questions. At the end of the interview, you will receive the results of the tests administered during the interview, along with their meaning and advice, via email.

Additionally, among the 1,694 participants, 126 (7.5% of the total) will be invited to participate in a sub-study to understand the impact of COVID-19 on cognition (memory and attention). If you are selected for this evaluation and consent to be contacted to participate, you will be invited to read a specific informed consent form and answer additional questions via video call, in which a qualified health professional will administer tests to assess memory and attention.

All these procedures will be conducted by trained researchers committed to confidentiality who will keep your information private. Telephone and/or video calls will be recorded for the sole purpose of auditing and reviewing the data, with access limited to the researchers involved.

The risks of participating in this study are minimal and related to the discomfort of answering some of the questions and the time dedicated to the interview. This study does not have direct benefits for you. The benefits of the study are indirect, that is, the results of the study will result in knowledge that can clarify the impact of COVID-19 on the Brazilian population.

Any information related to the study may be accessed solely by authorized individuals such as regulatory or health authorities. Due to the fact that the study procedures are conducted by telephone, it will be necessary to maintain a registry with your full name and contact information to ensure proper identification during the study procedures. This information will be exclusively accessible to the researchers who make the phone calls and will be recorded on forms separated from the other data you provide. Your personal data will not be shared and will not be accessible to Merck do Brasil, the funder of this study. All of your information will be treated confidentially and securely by the study researchers.

There will be no cost to you for participating in this study, and you will not receive any monetary compensation. If any expenses are incurred due to your participation in the study (such as telephone or internet expenses, just as exemples), the responsible researcher will guarantee reimbursement of such expenses. In case of harms resulting from your participation in the research, you will be entitled to compensation.

The results obtained from this study are for scientific purposes only. You are guaranteed that when the results are published, your name will not appear and your

identification will not be possible in any form. When the results are published, you will be notified in the most convenient way for you (phone, email, or letter) and you can access the information on the study website (www.covidlonga.com.br).

If you have any questions about the study, you may contact the principal investigator, Dr. Maicon Falavigna, by phone at (51) 9819-2778, at the address Avenida General Flores da Cunha, 1050, room 704, Cachoeirinha, Rio Grande do Sul, from Monday to Friday, 8:00 AM to 6:00 PM, or at any time by email at contato@covidlonga.com.br. This study has been analyzed and approved by the Ethics Committee (EC) of Hospital Moinhos de Vento. The EC is responsible for the evaluation and monitoring of the ethical aspects of all research involving human beings, aiming to guarantee the dignity, rights, safety, and well-being of research participants. If you have any doubts or questions about your rights as a participant in this study or if you are dissatisfied with the way the study is being conducted, please contact the EC of Hospital Moinhos de Vento, located at Rua Ramiro Barcelos, 910, 4th floor, phone 51-33143537, from Monday to Friday, 8:00 AM to 12:00 AM and 1:00 PM to 6:00 PM.

Participant decision

By checking the "YES, I consent to participate in the study voluntarily" option, you agree to voluntarily participate in this study. You confirm that you have been duly informed and clarified about the objective of this study, that you have read or someone have had read to you the procedures involved, as well as the possible risks and benefits arising from your participation, and that you have had all your doubts clarified until this moment. You confirm that you have been assured that you may refuse to participate and withdraw your consent at any time, without this causing any prejudice, penalty, or liability to you or your family. You authorize the disclosure of the data obtained in this study while maintaining your identity confidential. You confirm that the researcher who conducted the consent procedures to participate in this research has assured you that you will receive a copy of this document.

If you **do not want to participate in the study**, you should check the option "NO, I do not want to participate in the study." After that, you will no longer be contacted.

Do you consent to participate in the study?

( ) YES, I consent to participate in the study voluntarily.

( ) NO, I do not want to participate in the study.

*Explanation >> If the prospective participant have selected **"NO, I do not want to participate in the study"**, the following message will appear:*

> **You have decided NOT to participate in the study.**
>
> To confirm this decision, please click on FINISH.
>
> If you wish to change your decision, click on BACK.

*Explanation >> If the potential participant selects "**FINISH**," the process is concluded, and they will receive an email with the completed e-ICF reflecting this decision. The ICF sent by mail will*

include their name, the date and time they registered the decision, and the name of the researcher who provided and administered the e-ICF.

*Explanation >> If the prospective participant has selected **"YES, I consent to participate in the study voluntarily"**, the participant will be asked about the permission to be contacted in the future to be invited to the cognition substudy.*

Regarding the invitation to participate in the cognitive assessment substudy (memory and attention), in case you be selected:

( ) YES, I accept to be invited to participate in the substudy, if I were selected.

( ) NO, I do not want to be invited to the substudy, if I were selected.

*Explanation >> If the prospective participant has selected **"YES, I consent to participate in the study voluntarily",** and **"YES, I accept to be invited to participate in the substudy, if I were selected"**, the following message will appear:*

You have consented to participate in the study and to be invited to participate in the sub-study, if selected.

To confirm this decision, please click on FINISH.

If you wish to change your decision, click on BACK.

Explanation >> If the potential participant selects "FINISH," the consent process is completed and the participant will receive an email with the completed e-ICF reflecting the decision, including their name, date and time of registration, as well as the name of the person who provided and administered the e-ICF.

*Explanation >> If the prospective participant has selected **"YES, I consent to participate in the study voluntarily",** and **"NO, I do not want to be invited to the substudy, if I were selected"**, the following message will appear:*

You have consented to participate in the research, but have NOT consented to be invited to participate in the substudy, if selected.

>> By selecting "FINISH," the consent process is completed, and the participant will receive an email with the completed e-ICF reflecting the decision, including their name, date and time of registration, as well as the name of the person who provided and administered the e-ICF:

Informed Consent Form version 2, dated July 31, 2023.
Participant's name: automatically generated by the system.
Date and time of consent registration by the participant: automatically generated by the system.

Researcher's statement. I declare that I obtained in an appropriate and voluntary manner the informed consent of this participant for the participation in this study. I further declare that I commit to comply with what is described in this ICF.

Name of the researcher who provided and administered the e-ICF: automatically generated by the system.

>> NOTE: the descriptions in italics are scripts about the e-ICF registry process, they do not appear in the the form. The e-ICF does not have page numbers because the digital visualization of the document does not have page breaks.

INFORMED CONSENT FORM

Version 2, July 31, 2023

Title: Incidence, associated factors and impact of the post-COVID-19 condition in Brazil during the Omicron phase: an observational study – Long COVID Brazil Study / **Cognition Substudy**

Principal Investigator: Maicon Falavigna – CRM-RS 31.743

Sponsor: INOVA MEDICAL – HEALTH SERVICES AND MANAGEMENT

Site: Brazil

You are invited to voluntarily participate in a **substudy for cognition assessment**, that is as part of the study entitled "Incidence, associated factors and impact of the post-COVID-19 condition in Brazil during the Omicron phase: an observational study", the ***Long COVID Brazil Study***. We are inviting you because you consented to be invited to participate if you were selected. This study is also being done across Brazil by Inova Medical and funded by Merck Sharp Dohme (MSD) LTD.

Before deciding to participate in this study, it is important that you understand why this study is being conducted, all the procedures involved, the potential benefits, risks, and discomforts that will be described and explained below. Participating or not participating in this study will not influence any type of healthcare you are currently receiving or will receive in the future.

At any time, before, during, or after the study, you may request information and clarification, refuse to participate, or withdraw from the study. In any of these cases, you will not suffer any negative consequences.

After reading this informed consent form and having your questions answered about the study, you will need to record at the end of this digital document whether or not you consent to participate in the study. Your voluntary consent to participate in the study will only occur if, at the end of reading this document, you check the box marked "YES, I consent to participate in the study voluntarily." If you do not wish to participate in the study, you should check the box marked "NO, I do not want to participate in the study." Regardless of your decision, this form will be sent to your email address, with a record of your decision, for your future reference. If you wish, we will send you by mail a printed copy of the document with your recorded decision.

This study aims to understand the post-COVID-19 effects on the cognition function (memory and attention) in adults who have had COVID-19 during the dissemination of the Omicron variant in Brazil.

If you agree to participate, the study procedures will involve a detailed assessment of your memory and attention (cognitive function), by a health professional certified to a bundle of specific cognitive tests that will be conducted by a video call. This procedure will take approximately 45 minutes, and will be scheduled at a time that is most convenient for you, considering the recommendation that you be in a comfortable and safe place to answer the

study questions. The video calls will be recorded for the sole purpose of auditing and reviewing the data, with access limited to the researchers involved. You will receive via email the results of the cognitive tests administered during the video call, along with their meaning and advice.

The risks of participating in this study are minimal and related to the discomfort of answering some of the questions and the time dedicated to the interview. This study does not have direct benefits for you. The benefits of the study are indirect, that is, the results of the study will result in knowledge that can clarify the impact of COVID-19 on the Brazilian population.

Any information related to the study may be accessed solely by authorized individuals such as regulatory or health authorities. Due to the fact that the study procedures are conducted by video call, it will be necessary to maintain a registry with your full name and contact information to ensure proper identification during the study procedures. This information will be exclusively accessible to the researchers who make the phone calls and will be recorded on forms separated from the other data you provide. Your personal data will not be shared and will not be accessible to Merck do Brasil, the funder of this study. All of your information will be treated confidentially and securely by the study researchers.

There will be no cost to you for participating in this study, and you will not receive any monetary compensation. If any expenses are incurred due to your participation in the study (such as telephone or internet expenses, just as exemples), the responsible researcher will guarantee reimbursement of such expenses. In case of harms resulting from your participation in the research, you will be entitled to compensation.

The results obtained from this study are for scientific purposes only. You are guaranteed that when the results are published, your name will not appear and your identification will not be possible in any form. When the results are published, you will be notified in the most convenient way for you (phone, email, or letter) and you can access the information on the study website (www.covidlonga.com.br).

If you have any questions about the study, you may contact the principal investigator, Dr. Maicon Falavigna, by phone at (51) 9819-2778, at the address Avenida General Flores da Cunha, 1050, room 704, Cachoeirinha, Rio Grande do Sul, from Monday to Friday, 8:00 AM to 6:00 PM, or at any time by email at contato@covidlonga.com.br. This study has been analyzed and approved by the Ethics Committee (EC) of Hospital Moinhos de Vento. The EC is responsible for the evaluation and monitoring of the ethical aspects of all research involving human beings, aiming to guarantee the dignity, rights, safety, and well-being of research participants. If you have any doubts or questions about your rights as a participant in this study or if you are dissatisfied with the way the study is being conducted, please contact the EC of Hospital Moinhos de Vento, located at Rua Ramiro Barcelos, 910, 4th floor, phone 51-33143537, from Monday to Friday, 8:00 AM to 12:00 AM and 1:00 PM to 6:00 PM.

Participant decision

By checking the "YES, I consent to participate in the study voluntarily" option, you agree to voluntarily participate in this study. You confirm that you have been duly informed and clarified about the objective of this study, that you have read or someone have had read to you

the procedures involved, as well as the possible risks and benefits arising from your participation, and that you have had all your doubts clarified until this moment. You confirm that you have been assured that you may refuse to participate and withdraw your consent at any time, without this causing any prejudice, penalty, or liability to you or your family. You authorize the disclosure of the data obtained in this study while maintaining your identity confidential. You confirm that the researcher who conducted the consent procedures to participate in this research has assured you that you will receive a copy of this document.

If you **do not want to participate in the study**, you should check the option "NO, I do not want to participate in the study." After that, you will no longer be contacted.

Do you consent to participate in the study?

( ) YES, I consent to participate in the study voluntarily.

( ) NO, I do not want to participate in the study.

*Explanation >> If the prospective participant have selected **"NO, I do not want to participate in the study"**, the following message will appear:*

> **You have decided NOT to participate in the study.**
>
> To confirm this decision, please click on FINISH.
>
> If you wish to change your decision, click on BACK.

*Explanation >> If the potential participant selects "**FINISH**," the process is concluded, and they will receive an email with the completed e-ICF reflecting this decision. The ICF sent by mail will include their name, the date and time they registered the decision, and the name of the researcher who provided and administered the e-ICF.*

*Explanation >> If the prospective participant has selected **"YES, I consent to participate in the study voluntarily"**, the participant will be asked about the permission to be contacted in the future to be invited to the cognition substudy.*

> **You have consented to participate in the study voluntarily.**
> To confirm this decision, please click on FINISH.
> If you wish to change your decision, click on BACK.

>> By selecting "FINISH," the consent process is completed, and the participant will receive an email with the completed e-ICF reflecting the decision, including their name, date and time of registration, as well as the name of the person who provided and administered the e-ICF:

Informed Consent Form version 2, dated July 31, 2023.
Participant's name: automatically generated by the system.
Date and time of consent registration by the participant: automatically generated by the system.

Researcher's statement. I declare that I obtained in an appropriate and voluntary manner the informed consent of this participant for the participation in this study. I further declare that I

commit to comply with what is described in this ICF.

Name of the researcher who provided and administered the e-ICF: automatically generated by the system.

>> NOTE: the descriptions in italics are scripts about the e-ICF registry process, they do not appear in the the form. The e-ICF does not have page numbers because the digital visualization of the document does not have page breaks.

PROTOCOLO DE ESTUDO CLÍNICO

Incidência, fatores associados e impacto da condição pós-COVID-19 no Brasil durante a fase Ômicron: Estudo observacional

Estudo COVID Longa Brasil

Versão 4 de 20 de abril de 2024.

Proponente: *INOVA Research*

Este documento é confidencial e seu uso, reprodução, divulgação e publicação são restritos aos pesquisadores responsáveis da INOVA Research – INOVA MEDICAL, ao patrocinador e ao financiador.

HISTÓRICO DE VERSÕES

Versão	Principais alterações
**1 de 02 de março de 2023**	Versão inicial
**2 de 31 de julho de 2023**	• Esclarecimento sobre obrigatoriedade de confirmação laboratorial de COVID-19 por RT PCR ou teste do antígeno, mas não obrigatoriedade de envio de documento comprobatório pelo participante.<br><br>• Substituição da Escala de Impacto do Evento Revisada (Impact of Event Scale Revised - IES-R) constituída de 22 questões para a avaliação de sintomas de estresse pós-traumático, pela sua versão reduzida e validada, a IES-6, que contém 6 questões. O uso do instrumento em sua apresentação reduzida foi preferido para melhorar a eficiência da entrevista, ou seja, obter resultado análogo, com menor tempo disponibilizado pelo participante. O IES-6 está sendo empregado desde o início do recrutamento, por meio de notificação ao CEP.<br>Adição da referência de validação da versão reduzida e respectivo ponto de corte: Thoresen S, Tambs K, Hussain A, Heir T, Johansen VA, Bisson JI. Brief measure of posttraumatic stress reactions: impact of event scale-6. Soc Psychiatry Psychiatr Epidemiol. 2010;45:405–12.<br>Anexo_2_Modelo_relatorio_resultados_testes, ajustado para _v2_31_jul_2023, contemplando a substituição do instrumento.<br><br>• Inclusão de itens no cadastro de interesse em participar do estudo (nome completo, pronome, cidade). Anexo_1_Plano_de_recrutamento_e_divulgacao ajustado para _v2_31_jul_2023, contemplando inclusão dos itens e atualização dos materiais de divulgação.<br><br>• Modificação para registro do consentimento no e-TCLE, ao invés de processo de rubrica e assinatura digital.<br><br>• Revisão do formulário de coleta de dados com retirada de itens não aplicáveis às análises pretendidas.<br><br>• Redução do tempo médio de entrevista de 1 hora (60 min) para 50 min, decorrente da revisão da ficha de coleta de dados e do uso do instrumento reduzido para avaliação dos sintomas de estresse pós traumático (IES-6).
**3 de 15 de setembro de 2023**	• Correção da versão do Subteste de Dígitos e inclusão da Escala de Avaliação de Disfunções Executivas de Barkley (BDEFS) na bateria de avaliações que constitui a entrevista para o subestudo de cognição.<br><br>• Revisão do formulário de coleta de dados na sessão referente ao subestudo de cognição para contemplar o item corrigido e o item incluído, bem como a ordem de realização dos testes cognitivos.
**4 de 20 de abril de 2024**	• Correção do ponto de corte utilizado para definição de caso e controle no subestudo de avaliação da cognição, por meio do escore da Entrevista telefônica para avaliação do estado cognitivo modificada (TICS-m), de 31 para 21 (erro de grafia).<br><br>• Inclusão de modalidade de recrutamento presencial (contato inicial, convite e processo de consentimento) realizado por recrutadores locais treinados pela equipe do estudo. Anexo_1_Plano_de_recrutamento_e_divulgacao ajustado para _v3_20_abr_2024, contemplando essa atualização.<br><br>• Revisão do formulário de coleta de dados para inclusão do anos de 2024 nas possibilidades de data para casos de COVID-19 e data de vacinação.

INFORMAÇÕES ADMINISTRATIVAS

Título

Incidência, fatores associados e impacto da condição pós-COVID-19 no Brasil durante a fase Ômicron: Estudo observacional

Acrônimo

Estudo COVID Longa Brasil

Pesquisadores responsáveis

Maicon Falavigna, MD, MSc, PhD
Link currículo *Lattes*: http://lattes.cnpq.br/6242018987560550

Regis Goulart Rosa, MD, MSc, PhD
Link currículo *Lattes*: http://lattes.cnpq.br/5047175130077041

Colaboradores

Caroline Cabral Robinson, MSc, PhD
Link currículo *Lattes*: http://lattes.cnpq.br/4097704323332933

Daniel Sganzerla, BSc
Link currículo *Lattes:* http://lattes.cnpq.br/3781964610442096

Josélia Larger Manfio, PhD
Link currículo *Lattes:* http://lattes.cnpq.br/5736470882499170

Instituição proponente

INOVA Research, departamento da INOVA MEDICAL - SERVICOS DE SAÚDE E GESTÃO LTDA

Patrocinador

INOVA MEDICAL - SERVICOS DE SAÚDE E GESTÃO LTDA

Apoio financeiro

Grant de pesquisa - *Merck Investigator Studies Program*

Versão do protocolo

Versão 4, de 20 de abril de 2024.

LISTA DE ABREVIATURAS E SIGLAS

CEP	Comitê de ética em pesquisa
CONEP	Comissão nacional de ética em pesquisa
COVID-19	Doença causada pelo SARS-CoV-2
EQ-5D-3L	Escala EuroQol de 5 dimensões e 3 níveis
HADS	Escala hospitalar de sintomas de ansiedade e depressão
ICH	*International concil of harmonization*
ICMJE	*International committee of medical journal editors*
IES-6	Escala de impacto de evento versão reduzida em 6 itens
LGPD	Lei geral de proteção de dados
MISP	*Merck investigator studies program*
MRC	*Medical Research Council*
OMS	Organização mundial da saúde
RDC	Resolução de diretoria colegiada
SARS-CoV-2	Coronavirus 2 da síndrome respiratória aguda grave
TCLE	Termo de consentimento livre e esclarecido
TICS-m	Entrevista telefônica para avaliação do estado cognitivo modificada
tMoCA	Avaliação cognitiva de Montreal por telefone

SUMÁRIO

SINOPSE 6
INTRODUÇÃO 10
JUSTIFICATIVA 13
OBJETIVOS 14
Objetivo primário 14
Objetivos secundários 14
MÉTODOS 15
Desenho do estudo 15
Recrutamento de participantes da pesquisa 15
Critérios de elegibilidade 16
Exposições 17
Desfechos 19
Procedimentos do estudo 20
Tamanho amostral 23
Análise estatística 23
ÉTICA E BOAS PRÁTICAS CLÍNICAS 24
Consentimento para participação 25
Potenciais riscos e benefícios 26
Confidencialidade 27
ORGANIZAÇÃO DO ESTUDO 28
Centro coordenador 28
Comitê diretivo 28
Patrocinador 28
Apoio financeiro 29
POLÍTICA DE DISSEMINAÇÃO 29
Plano de disseminação dos resultados 30
Política de autoria 30
Política de compartilhamento de dados 30
REFERÊNCIAS 31

SINOPSE

**Título do estudo**	Incidência, fatores associados e impacto da condição pós-COVID-19 no Brasil durante a fase Ômicron: Estudo observacional
**Objetivos**	**Objetivo primário**<br>● Avaliar a associação entre condição pós-COVID-19 de acordo com a definição da Organização Mundial da Saúde (OMS) e qualidade de vida relacionada à saúde em adultos com histórico de infecção sintomática pelo SARS-CoV-2 durante a fase Ômicron no Brasil.<br><br>**Objetivos secundários**<br>● Avaliar a incidência de condição pós-COVID-19 em adultos no Brasil durante a fase Ômicron de acordo com a definição de caso da OMS;<br>● Descrever os sintomas relatados de condição pós-COVID-19;<br>● Avaliar potenciais fatores de risco para a condição pós-COVID-19 em adultos com histórico de COVID-19 sintomática durante a fase Ômicron no Brasil;<br>● Avaliar a associação entre condição pós-COVID-19 de acordo com a definição da OMS e os seguintes desfechos em adultos:<br>1) Atividades instrumentais de vida diária;<br>2) Capacidade física funcional;<br>3) Cognição;<br>4) Sintomas de ansiedade;<br>5) Sintomas de depressão;<br>6) Sintomas de estresse pós-traumático;<br>7) Retorno ao trabalho;<br>8) Retorno aos estudos;<br>9) Utilização de recursos de saúde e seus custos;<br>● Comparar domínios da cognição entre casos de disfunção cognitiva pós-COVID-19 e controles sem disfunção cognitiva pós-COVID-19 pareados por faixa etária, sexo, nível de escolaridade e tempo desde o diagnóstico de COVID-19 (estudo caso-controle aninhado).
**Desenho do estudo**	Estudo de coorte nacional com recrutamento em ambiente virtual e presencial para avaliação da incidência, impacto e fatores associados com a condição pós-COVID-19 com estudo caso-controle aninhado para comparação dos domínios de cognição entre casos de disfunção cognitiva pós-COVID-19 e controles sem disfunção cognitiva pós-COVID-19.
**Recrutamento**	O estudo de coorte recrutará participantes através de anúncios em sites eletrônicos, mídias sociais, divulgação e busca presencial por potenciais

	participantes. Para o estudo caso-controle aninhado, uma amostra de casos e controles pareada por faixa etária, sexo, nível de escolaridade e tempo desde o diagnóstico de COVID-19 será selecionada a partir da coorte.
**Participantes**	**Critérios de inclusão**<br>● Idade maior ou igual a 18 anos;<br>● Residência no Brasil;<br>● Histórico de infecção sintomática* pelo SARS-CoV-2 confirmada por exame da reação da transcriptase reversa seguida pela reação em cadeia da polimerase (RT-PCR) ou teste de antígeno para SARS-CoV-2 ocorrido após 01 de janeiro de 2022.<br>*Pelo menos um dos seguintes sintomas: febre, congestão nasal, rinorreia, anosmia, dor de garganta, rouquidão, tosse, dispneia, sibilância e mialgia.<br><br>**Critérios de exclusão**<br>● Tempo entre início dos sintomas do episódio de COVID-19 e recrutamento < 90 dias;<br>● Ausência de disponibilidade ou impossibilidade para participar de entrevista remota;<br>● Dificuldade de comunicação (afasia, deficiência auditiva grave, demência severa, não nativos na língua portuguesa);<br>● Ausência de consentimento para participação no estudo;<br>● Participantes já incluídos no estudo.
**Exposições**	**Variável de exposição para avaliação do impacto da condição pós-COVID-19 em desfechos clínicos**<br>● Presença de condição pós-COVID-19 de acordo com a definição da OMS*<br>*Sintomas que surgem em até três meses após a contaminação, que duram pelo menos dois meses e não podem ser explicados por um diagnóstico alternativo.<br><br>**Variáveis de exposição para avaliação de fatores associados com a condição pós-COVID-19**<br>● Variáveis sócio demográficas;<br>● Condições de saúde pré-COVID-19;<br>● Histórico de infecção sintomática e documentada pelo SARS-COV-2 anterior ao episódio de COVID-19 sob estudo;<br>● Status vacinal para COVID-19;<br>● Severidade do episódio de COVID-19 de acordo com o pior nível da escala de severidade clínica da OMS;<br>● Tratamentos utilizados durante a fase aguda da COVID-19 (antivirais, corticoesteróides, imunomoduladores e anticorpos monoclonais contra SARS-CoV-2).

	**Variável de exposição para comparação dos domínios de cognição entre participantes com e sem disfunção cognitiva pós-COVID-19 (estudo de caso-controle aninhado)**<br>• Casos: Disfunção cognitiva de acordo com a versão brasileira da Entrevista Telefônica para Avaliação do Estado Cognitivo modificada (TICS-m; escore total ≤ 21);<br>• Controles: ausência de disfunção cognitiva de acordo com a versão brasileira da TICS-m (escore total > 21) pareados com os casos por faixa etária, sexo, nível de escolaridade e tempo desde o diagnóstico de COVID-19.
**Desfechos**	**Desfecho primário**<br>• Qualidade de vida relacionada à saúde avaliada pelo escore de utilidade da escala EuroQol de 5 dimensões e 3 níveis (EQ-5D-3L).<br><br>**Desfechos secundários**<br>• Grau de atividades instrumentais de vida diária aferido pela versão brasileira da escala de atividades instrumentais de vida diária de *Lawton & Brody*;<br>• Grau de capacidade física funcional aferido pela versão brasileira do índice Barthel;<br>• Grau de função cognitiva aferida pela versão brasileira da escala TICS-m;<br>• Sintomas de ansiedade e depressão aferidos pela versão brasileira da escala hospitalar de sintomas de ansiedade e depressão (HADS);<br>• Sintomas de estresse pós-traumático aferidos pela versão brasileira da escala de impacto de evento versão reduzida em 6 itens (IES-6);<br>• Retorno ao trabalho;<br>• Retorno aos estudos;<br>• Utilização de recursos de saúde (consultas médicas eletivas, consultas eletivas com profissionais de saúde não-médicos, atendimentos de urgência ou emergência, hospitalizações).<br><br>**Desfechos secundários avaliados nos participantes selecionados para o estudo de caso-controle aninhado**

	• Grau de função cognitiva aferida pela versão brasileira da Avaliação cognitiva de Montreal por telefone (tMoCA);<br>• Grau de função cognitiva aferida pela versão brasileira do teste de aprendizagem auditivo-verbal de Rey;<br>• Grau de função cognitiva aferida pela versão brasileira da Bateria Montreal-Toulouse de avaliação da linguagem – subteste de nomeação oral;<br>• Grau de função cognitiva aferida pela versão brasileira do teste de acentuação de palavras;<br>• Grau de função cognitiva aferida pela versão brasileira do subteste de contagem reversa Neupsilin.<br>• Grau de função cognitiva aferida pela versão brasileira do subteste de dígitos<br>• Grau de função executiva aferida pela versão brasileira da Escala de Avaliação de Disfunções Executivas de Barkley (BDFES).
**Avaliação das variáveis do estudo**	Para a coorte, será realizada avaliação das variáveis do estudo através de uma entrevista remota (telefônica ou videochamada) centralizada, estruturada e conduzida por profissionais de pesquisa treinados e capacitados em boas práticas clínicas. Participantes selecionados para o estudo caso-controle, participarão de uma entrevista remota adicional para avaliação dos domínios de cognição conduzidas por profissionais habilitados para a condução dos testes.
**Tamanho de amostra**	Estudo de coorte: 1.694 participantes.<br>Estudo caso-controle: 126 participantes selecionados a partir da coorte (63 casos com disfunção cognitiva e 63 controles sem disfunção cognitiva).

INTRODUÇÃO

A infecção causada pelo novo coronavírus (SARS-CoV-2), denominada COVID-19, já ultrapassou a marca de 37 milhões de pessoas infectadas no Brasil com mais 690 mil mortes.[1] Entre os sobreviventes de formas agudas da COVID-19, uma parcela relevante apresenta sintomas prolongados como fadiga, dispneia, tosse, anosmia, dificuldade de concentração, déficit de memória e sintomas de acometimento de saúde mental (ansiedade, depressão e estresse pós-traumático), os quais possuem potencial para impactar negativamente desfechos relevantes como qualidade de vida e gastos em saúde.[2,3]

A ocorrência precisa de sintomas prolongados após episódio de COVID-19 é desconhecida. De acordo com estimativas da Organização Mundial da Saúde (OMS), 10 a 20% dos pacientes infectados pelo SARS-CoV-2 pode apresentar sintomas persistentes em um quadro denominado condição pós-COVID-19 (ou COVID-19 longa).[4] Estudos observacionais publicados até o momento, demonstram uma ocorrência de sintomas prolongados que varia de 6 a 45% a depender da série avaliada de pacientes, com variações que podem ser explicadas por: 1) severidade da COVID-19; 2) variante predominante, cobertura vacinal e disponibilidade de tratamentos efetivos para a COVID-19 aguda durante o período de realização do estudo; 3) tempo de seguimento; bem como 4) potenciais confundidores como fragilidade pré-morbida e comorbidades associadas.[5-7] Em uma tentativa de estimar a ocorrência de sintomas prolongados atribuíveis à COVID-19 (após exclusão de casos cujos sintomas poderiam ser explicados por outras condições de saúde), uma coorte prospectiva conduzida na Holanda com mais de 76.000 participantes encontrou uma prevalência de sintomas prolongados atribuível à COVID-19 de 21%, 90 a 150 dias após o episódio índice de infecção pelo SARS-CoV-2[8] – um resultado próximo ao estimado pela OMS. Infelizmente, dados robustos e precisos a respeito da ocorrência de condição pós-COVID-19 no Brasil, especialmente no atual cenário de predominância de variante Ômicron (causa predominante de COVID-19 no Brasil desde janeiro de 2022, figura 1), alta cobertura vacinal e disponibilidade de tratamentos efetivos para fase aguda da COVID-19, são escassos. Esta lacuna de conhecimento, dificulta a implementação de políticas de saúde eficientes para prevenção e reabilitação precoce da condição pós-COVID-19.

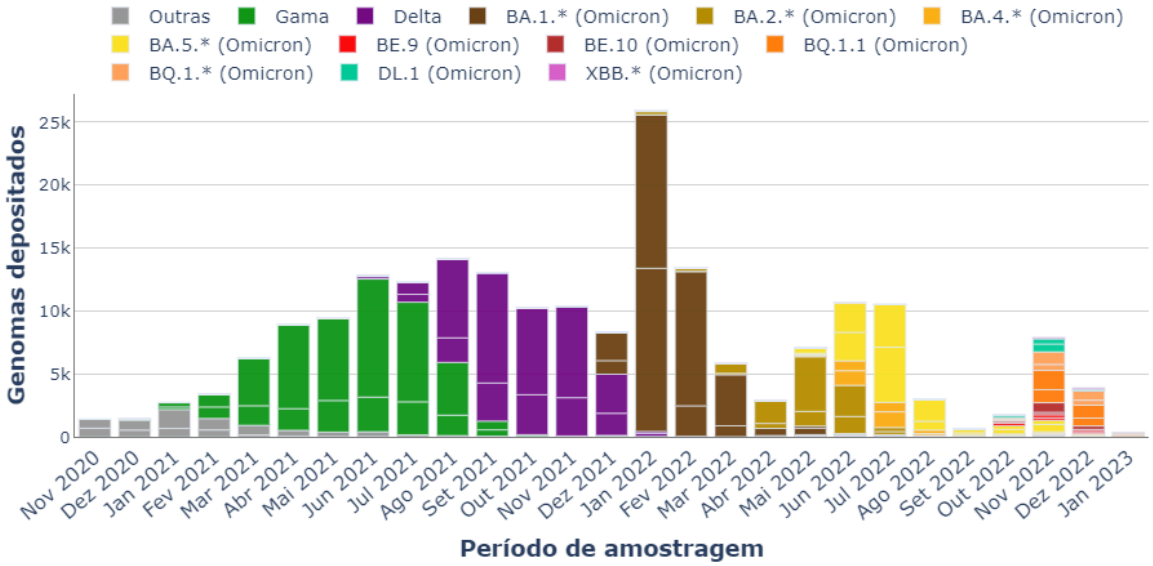

Figura 1. Variantes de SARS-CoV-2 responsáveis pela COVID-19 no Brasil.
Fonte: Rede genômica Fiocruz.

Dados a respeito do impacto da condição pós-COVID-19 em desfechos relevantes também são pouco frequentes e heterogêneos.[9] Apesar de estudos observacionais terem encontrado associação entre consequências da COVID-19 e pior qualidade de vida,[10-12] este achado é susceptível a potenciais vieses de seleção, confusão e aferição potencializados pelo uso de definições subjetivas da condição pós-COVID-19. Da mesma forma, apesar de alguns estudos observacionais apontarem sexo feminino, comorbidades, severidade do episódio de COVID-19 aguda e estados de privação social como potenciais fatores de risco para condição pós-COVID-19,[13] estes achados são inconsistentes entre os estudos disponíveis podendo ainda sofrer influência de fatores locais como variante predominante, grau de transmissão comunitária de SARS-CoV-2, status vacinal e acessibilidade à medidas de prevenção de COVID-19 e reabilitação. Portanto, estudos locais são necessários para geração de conhecimento acerca de populações de risco para o desenvolvimento da condição pós-COVID-19.

Para facilitação do diagnóstico e pesquisa no tópico, em outubro de 2021, a OMS publicou a definição de caso da condição pós-COVID-19, a qual foi elaborada após realização de consenso Delphi com a participação de *experts* e representantes de pacientes e familiares.[4] De acordo com a definição, a condição pós-COVID-19 se refere a "sintomas que surgem em até três meses após a contaminação, que duram pelo menos dois meses e não podem ser explicados por um diagnóstico alternativo". Entretanto, estudos avaliando

incidência, impacto em desfechos clínicos, bem como potenciais fatores de riscos para condição pós-COVID-19 de acordo com a definição de caso da OMS ainda são escassos.

Assim, de forma a contribuir para o fechamento das lacunas de evidência acima descritas, o presente estudo propõe-se a avaliar incidência, fatores associados e impacto da condição pós-COVID-19 de acordo com a definição de caso da OMS em pacientes adultos que apresentaram infecção documentada por SARS-CoV-2 durante a fase Ômicron no Brasil.

JUSTIFICATIVA

O atual contexto de um crescente número de pessoas apresentando consequências físicas, cognitivas e de saúde mental após um episódio de COVID-19 associada à escassez de dados de longo prazo tem tornado prioritário o estudo da incidência, fatores de risco e impacto da definição de caso da condição pós-COVID-19 em desfechos clínicos, especialmente no atual cenário de predominância de variante Ômicron, alta cobertura vacinal - porém com duração limitada da proteção - e disponibilidade de tratamentos efetivos para fase aguda da COVID-19. Os dados do presente estudo contribuirão para geração de evidência a respeito desse importante tópico, contribuindo para o conhecimento a respeito da condição pós-COVID-19 no Brasil e para o debate baseado em evidência a respeito de potenciais estratégias de prevenção, identificação e reabilitação de pacientes com sequelas pós-COVID-19.

OBJETIVOS

Objetivo primário

Avaliar a associação entre condição pós-COVID-19 de acordo com a definição da Organização Mundial da Saúde (OMS) e qualidade de vida relacionada à saúde em adultos com histórico de infecção sintomática pelo SARS-CoV-2 durante a fase Ômicron no Brasil.

Objetivos secundários

- Avaliar a incidência de condição pós-COVID-19 em adultos no Brasil de acordo com a definição de caso da OMS;
- Descrever os sintomas relatados de condição pós-COVID-19;
- Avaliar potenciais fatores de risco para a condição pós-COVID-19 em adultos;
- Avaliar a associação entre condição pós-COVID-19 de acordo com a definição da OMS e os seguintes desfechos em adultos:
  1) Atividades instrumentais de vida diária;
  2) Capacidade física funcional;
  3) Cognição;
  4) Sintomas de ansiedade;
  5) Sintomas de depressão;
  6) Sintomas de estresse pós-traumático;
  7) Retorno ao trabalho;
  8) Retorno aos estudos;
  9) Utilização de recursos de saúde e seus custos estimados.
- Comparar domínios da cognição entre casos de disfunção cognitiva pós-COVID-19 e controles sem disfunção cognitiva pós-COVID-19 pareados por idade, sexo, nível de escolaridade e tempo desde o diagnóstico de COVID-19 (estudo caso-controle aninhado).

MÉTODOS

Desenho do estudo

Trata-se de um estudo de coorte nacional com recrutamento em ambiente virtual e presencial com estudo caso-controle aninhado (figura 2). O estudo de coorte avaliará a incidência, o impacto e os fatores associados com a condição pós-COVID-19. O estudo caso-controle comparará os domínios de cognição entre casos de disfunção cognitiva pós-COVID-19 e controles sem disfunção cognitiva pós-COVID-19 pareados por faixa etária, sexo, nível de escolaridade e tempo desde o diagnóstico de COVID-19.

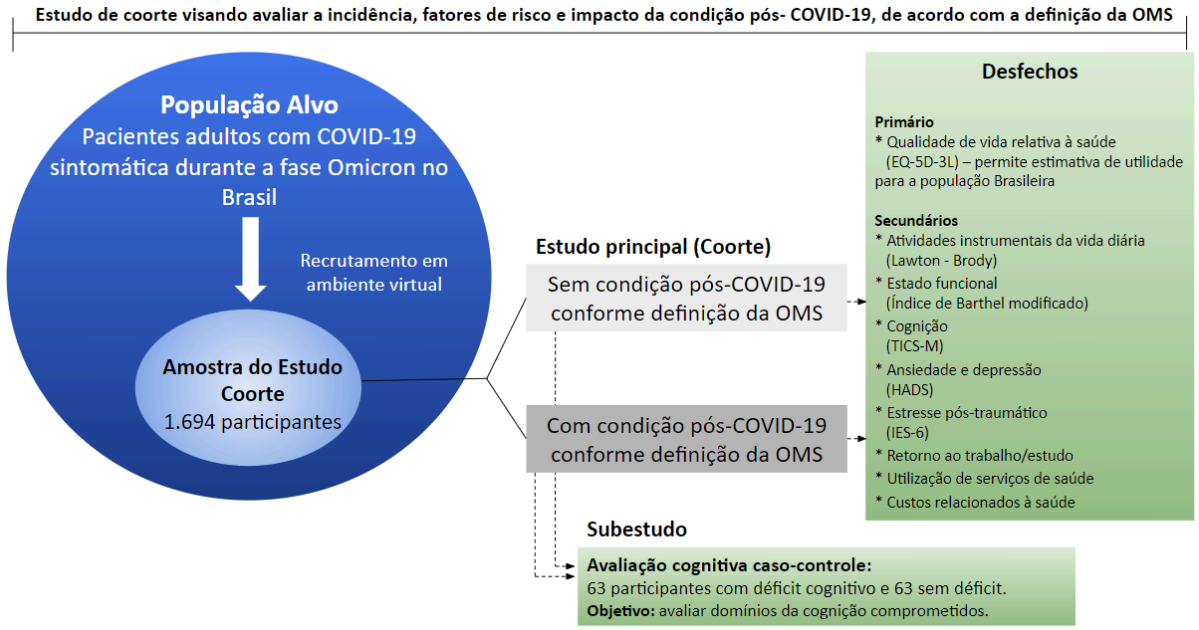

Figura 2. Desenho do estudo.
EQ-5D-3L, escala EuroQol de 5 dimensões e 3 níveis; HADS, escala hospitalar de sintomas de ansiedade e depressão; IES-6, Escala de impacto de evento de 6 itens; TICS-M, entrevista telefônica para avaliação do estado cognitivo modificada; tMOCA, avaliação cognitiva de Montreal por telefone.

Recrutamento de participantes da pesquisa

O estudo recrutará participantes das 5 regiões geopolíticas do Brasil através de anúncios públicos em sites eletrônicos, mídias sociais, divulgação e busca ativa presencial por potenciais participantes. O recrutamento presencial será realizado por recrutadores treinados e delegados pelo pesquisador responsável. A amostragem de participantes será estratificada por região de residência (Norte, Nordeste, Sudoeste, Centro-Oeste e Sul) e faixa etária (≥ 18 a < 40 anos; ≥ 40 a < 60 anos; ≥ 60 anos) conforme descrito na tabela 1.

Tabela 1. Extratos de recrutamento de acordo com região de residência e faixa etária.

	Região de Residência				
	**Norte**	**Nordeste**	**Sudoeste**	**Centro-Oeste**	**Sul**
**≥18 a <40 anos**	4,4%	12,7%	18,4%	4,1%	6,9%
**≥40 a <60 anos**	2,6%	8,7%	14,7%	2,7%	4,9%
**≥60 anos**	1,1%	4,7%	9,4%	1,3%	3,3%

Os processos de divulgação do estudo e recrutamento estão apresentados em detalhe no **Anexo 1** (Plano de recrutamento e divulgação). A divulgação do estudo ocorrerá por meio de uma página eletrônica que apresentará informações a respeito dos objetivos do estudo, critérios de elegibilidade, riscos e benefícios da participação no estudo e procedimentos da pesquisa. A divulgação presencial e busca ativa por participantes também direcionará os interessados para a página do estudo. Pessoas que demonstrarem interesse na participação do estudo realizarão um cadastro com informações básicas para contato, e serão contatadas por pesquisadores de forma remota para orientações sobre o estudo e aplicação do termo de consentimento livre e esclarecido (TCLE). Pessoas que demonstrarem interesse a partir do contato presencial já receberão orientações sobre o estudo e acesso ao TCLE, podendo realizar o processo de consentimento junto com o pesquisador que realizou o contato presencial, caso desejarem.

Para o estudo caso-controle aninhado, uma amostra de casos de disfunção cognitiva pós-COVID-19 e controles sem disfunção cognitiva pós-COVID-19 será selecionada a partir da coorte. Casos e controles serão pareados por faixa etária, sexo, nível de escolaridade e tempo desde o diagnóstico de COVID-19 (± 30 dias). Indivíduos potencialmente elegíveis para o estudo caso-controle, que consentiram serem contatados para esse subestudo caso selecionados, serão contatados remotamente para orientações sobre o estudo aninhado e aplicação de TCLE específico.

Critérios de elegibilidade

Critérios de inclusão

- Idade maior ou igual a 18 anos;
- Residência no Brasil;
- Histórico de infecção sintomática pelo SARS-CoV-2 confirmada por exame da reação da transcriptase reversa seguida pela reação em cadeia da polimerase (RT-PCR) ou teste de antígeno para SARS-CoV-2 ocorrido após 01 de janeiro de 2022. Será considerada infecção sintomática pelo SARS-CoV-2, aquela que tiver ocorrido na presença de pelo menos um dos seguintes sintomas: febre, congestão nasal, rinorreia, anosmia, dor de garganta, rouquidão, tosse, dispneia, sibilância e mialgia. O diagnóstico deverá ser comprovado por laudo emitido pelo local de realização do teste ou por registro no ConecteSUS, quaisquer dessas opções disponibilizadas ou relatadas pelo participante.

Critérios de exclusão

- Tempo entre início dos sintomas do episódio de COVID-19 e recrutamento < 90 dias;
- Ausência de disponibilidade ou impossibilidade para participar de entrevista remota;
- Dificuldade de comunicação (afasia, deficiência auditiva grave, demência severa, não nativos na língua portuguesa);
- Ausência de consentimento para participação no estudo;
- Participantes já incluídos no estudo.

Exposições

Variável de exposição para avaliação do impacto da condição pós-COVID-19 em desfechos clínicos

- Presença de condição pós-COVID-19 de acordo com a definição da OMS: Sintomas que surgem em até três meses após a contaminação, que duram pelo menos dois meses e não podem ser explicados por um diagnóstico alternativo.[4]

  Os sintomas relatados de condição pós-COVID-19 serão coletados e descritos por tipo específico de sintoma (dispneia, tosse, alteração de olfato, alteração de paladar, fadiga, mal-estar pós-esforço, cefaléia, parestesias, dificuldade de concentração, dificuldade de memória, insônia, ansiedade, depressão, dor articular, dor muscular e outros) e sistema acometido (neurológico, saúde mental, respiratório, constitucional ou outros). Caso o participante reporte dispneia, esta será quantificada através da versão brasileira da escala de dispneia do *Medical Research Council* (MRC).[14] Caso o

participante reporte fadiga, esta será quantificada através da versão brasileira da escala de severidade de fadiga.[15]

Variáveis de exposição para avaliação de fatores associados com a condição pós-COVID-19

- Variáveis sócio demográficas: idade, sexo, escolaridade, tabagismo, consumo de álcool;
- Condições de saúde pré-COVID-19: comorbidades de acordo com o índice de comorbidades de Charlson,[16] histórico de ansiedade ou depressão, histórico de disfunção cognitiva, índice de massa corpórea e fragilidade de acordo com a escala de fragilidade clínica;[17]
- Histórico de infecção sintomática e documentada pelo SARS-COV-2 anterior ao episódio de COVID-19 sob estudo;
- Status vacinal para COVID-19 até a data do episódio sob estudo da infecção pelo SARS-COV-2;
- Severidade do episódio de COVID-19 sob estudo de acordo com o pior nível da escala de severidade clínica da OMS (1- tratamento em domicílio sem limitações para atividades de vida diária; 2- tratamento em domicílio com limitações para atividades de vida diária; 3- tratamento hospitalizado sem necessidade de terapia com oxigênio; 4- tratamento hospitalizado com necessidade de terapia com oxigênio por máscara ou cateter nasal de baixo fluxo; 5 - tratamento hospitalizado com necessidade de terapia com oxigênio por cateter nasal de alto fluxo ou ventilação não-invasiva; 6- tratamento hospitalizado com necessidade de ventilação mecânica invasiva);[18]
- Tratamentos utilizados durante a fase aguda da COVID-19 os quais incluem antivirais (molnupiravir, nirmatrelvir/ritonavir, remdesivir), corticoesteróides (dexametasona, predinisona, predinisolona, metilpredinisolona e hidrocortisona), imunomoduladores (tocilizumabe, baricitinibe) e anticorpos monoclonais contra SARS-CoV-2 (casirivimabe/imdevimabe, banlanivimabe/etesevimabe, regdanvimabe e sotrovimabe);

Variável de exposição para comparação dos domínios de cognição entre participantes com e sem disfunção cognitiva pós-COVID-19

- Casos: Disfunção cognitiva de acordo com a versão brasileira da entrevista telefônica para avaliação do estado cognitivo modificada (TICS-m; escore total ≤ 21 pontos);[19]
- Controles: Ausência de disfunção cognitiva de acordo com a versão brasileira da TICS-m (escore total > 21 pontos) pareados com os casos por faixa etária, sexo, nível de escolaridade e tempo desde o diagnóstico de COVID-19.

Desfechos

Desfecho primário

O desfecho primário é o escore de utilidade de qualidade de vida relacionada à saúde avaliado por meio da versão brasileira da escala EuroQol de 5 dimensões e 3 níveis (EQ-5D-3L).[20] O EQ-5D-3L compreende um sistema descritivo com cinco dimensões de qualidade de vida (mobilidade, autocuidado, atividades usuais, dor/desconforto e ansiedade/depressão). O escore de utilidade na população brasileira varia de -0,17 a 1,0 com maiores escores representando melhor qualidade de vida.[21] A diferença mínima clinicamente significativa varia de 0,03 a 0,52.[22]

Desfechos secundários

As medidas secundárias de desfecho incluem as variáveis descritas abaixo:

- Grau de atividades instrumentais de vida diária (uso de telefone, locomoção, compras, responsabilidade pelas próprias medicações finanças e habilidade para lidar com finanças) aferido pela versão brasileira da escala de atividades instrumentais de vida diária de *Lawton & Brody*;[23]
- Grau de capacidade física funcional aferido pela versão brasileira do índice Barthel;[24]
- Grau de função cognitiva aferida pela versão brasileira da escala TICS-m;[19]
- Sintomas de ansiedade e depressão aferidos pela versão brasileira da escala hospitalar de sintomas de ansiedade e depressão (HADS);[25]
- Sintomas de estresse pós-traumático aferidos pela versão brasileira da escala de impacto de evento versão reduzida em 6 itens (IES-6);[26,27]
- Retorno ao trabalho;
- Retorno aos estudos;

- Utilização de recursos de saúde (consultas médicas eletivas, consultas eletivas com profissionais de saúde não-médicos, atendimentos de urgência ou emergência, hospitalizações referidas pelo participante).

Desfechos secundários avaliados nos participantes selecionados para o estudo de caso-controle aninhado

- Grau de função cognitiva aferida pela versão brasileira da Avaliação cognitiva de Montreal por telefone (tMoCA),[28]
- Grau de função cognitiva aferida pela versão brasileira do teste de aprendizagem auditivo-verbal de Rey,[29]
- Grau de função cognitiva aferida pela versão brasileira da Bateria Montreal-Toulouse de avaliação da linguagem – subteste nomeação oral,[30]
- Grau de função cognitiva aferida pela versão brasileira do teste de acentuação de palavras,[31]
- Grau de função cognitiva aferida pela versão brasileira do teste de contagem reversa Neupsilin – subteste contagem inversa,[32]
- Grau de função cognitiva aferida pela versão brasileira do subteste de dígitos,[33]
- Grau de função executiva aferida pela versão brasileira da Escala de Avaliação de Disfunções Executivas de Barkley (BDFES).[34]

Procedimentos do estudo

Triagem de participantes e aplicação de Termo de Consentimento Livre e Esclarecido (TCLE)

Indivíduos que demonstrarem interesse, por meio de castrado na página eletrônica do estudo, serão inicialmente contatados por telefone ou mensagem de texto (conforme preferência informada no cadastro), considerando a estratificação da amostra de acordo com região de residência e faixa etária. Indivíduos potencialmente elegíveis, contatados por telefone, serão convidados a participar voluntariamente do estudo mediante condução do processo de consentimento e registro do consentimento em TCLE, que será aplicado por via eletrônica (e-TCLE). Indivíduos contatados presencialmente por pesquisador do estudo e que manifestarem interesse em participar, também farão seu registro na página eletrônica do estudo. Caso desejem, já poderão seguir para o processo de consentimento que será

conduzido pelo pesquisador de forma presencial, que disponibilizará acesso ao e-TCLE. As entrevistas serão sempre conduzidas por telefone, podendo o agendamento da entrevista ocorrer no momento do contato presencial. O processo de consentimento é detalhado na seção de ética e boas práticas clínicas deste protocolo. O participante que consentir participar, seguirá para a confirmação dos critérios de elegibilidade. Caso o participante não tenha realizado teste PCR ou antígeno para confirmação do diagnóstico de COVID-19, a participação será encerrada e não serão solicitadas informações adicionais. O contato com o potencial participante da pesquisa será realizado por pesquisadores devidamente treinados quanto às boas práticas clínicas, mantendo o anonimato e sigilo dos dados, bem como promovendo melhores condições de conforto e segurança ao potencial participante e, no caso do consentimento expresso, ao participante. O detalhamento desse processo está disponível no **Anexo 1** (Plano de recrutamento e divulgação).

Coleta de dados

Os participantes incluídos no estudo de coorte serão avaliados em uma única entrevista por pesquisador treinado e capacitado em boas práticas clínicas. A entrevista ocorrerá dentro de uma janela de 0 a 15 dias após assinatura do TCLE pelo participante. Participantes selecionados para o estudo caso-controle participarão de uma entrevista adicional para avaliação dos domínios de cognição, caso consentirem inicialmente em serem contatados e após a assinatura de TCLE específico para esse subestudo. Todas as entrevistas serão realizadas de forma remota (contato telefônico ou videochamada), estruturada e centralizada por pesquisadores da *INOVA Research* treinados para a coleta de dados nesta modalidade e habilitados para aplicação dos testes específicos do estudo caso-controle. Estima-se que a entrevista do estudo de coorte tenha duração de 50 minutos e a entrevista do estudo caso-controle tenha duração de 45 minutos. A tabela 2 sumariza as variáveis que serão coletadas no estudo.

Tabela 2. Dados a serem coletados no presente estudo.

**Dados**	**Estudo de coorte**	**Estudo caso-controle**
Data de nascimento	X	X
Sexo	X	X
Escolaridade	X	X
Informações sobre tabagismo	X	X
Informações sobre consumo de álcool	X	X
Índice de comorbidade de Charlson	X	X
Histórico de ansiedade ou depressão	X	X
Histórico de disfunção cognitiva	X	X

Índice de massa corpórea	X	X
Informações sobre fragilidade (escala de fragilidade clínica)	X	X
Informações sobre infecções prévias pelo SARS-COV-2	X	X
Informações sobre status vacinal para COVID-19	X	X
Informações sobre presença e intensidade de sintomas de COVID-19 longa (dispneia, tosse, alteração de olfato, alteração de paladar, fadiga, mal-estar pós-esforço, cefaleia, parestesias, dificuldade de concentração, dificuldade de memória, insônia, ansiedade, depressão, dor articular, dor muscular e outros)	X	X
Informação sobre qualidade de vida (EQ5D-3L) – Como era antes do episódio índice de COVID (1 mês antes do episódio) e como é agora	X	X
Informações sobre atividades instrumentais de vida diária (escala de *Lawton & Brody*) – Como era antes do episódio índice de COVID (1 mês antes do episódio) e como é agora	X	X
Informações sobre capacidade física funcional (índice *Barthel* modificado) – Como era antes do episódio índice de COVID (1 mês antes do episódio) e como é agora	X	X
Informações sobre função cognitiva (TICS-m)	X	X
Informações sobre sintomas de ansiedade e depressão (HADS)	X	X
Informações sobre sintomas de transtorno de estresse pós-traumático (IES-6)	X	X
Informações sobre retorno ao trabalho	X	X
Informações sobre retorno ao estudo	X	X
Informações sobre utilização de recursos de saúde (consultas médicas eletivas, consultas eletivas com profissionais de saúde não-médicos, atendimentos de urgência ou emergência, hospitalizações)	X	X
Informações sobre grau de função cognitiva em múltiplos domínios (t-MOCA, teste de aprendizagem auditivo-verbal de Rey, bateria Montreal-Toulouse de avaliação da linguagem – subteste nomeação oral, Teste de acentuação de palavras, Teste de contagem reversa Neupsilin – subteste contagem inversa, Subteste de dígitos e BDEFS)	N/A	X

EQ-5D-3L, escala EuroQol de 5 dimensões e 3 níveis; HADS, escala hospitalar de sintomas de ansiedade e depressão; IES-R, Escala de impacto de evento revisada; N/A, não se aplica; TICS-M, entrevista telefônica para avaliação do estado cognitivo modificada; tMOCA, avaliação cognitiva de Montreal por telefone.

Gerenciamento de dados

A coleta de dados será realizada com o uso de fichas clínicas eletrônicas preenchidas pelo pesquisador que conduzirá a entrevista telefônica a partir dos dados relatados pelo participante. O processo de coleta e gestão de dados de forma digital apresenta diversos benefícios em relação ao processo não automatizado, dentre eles estão a padronização, a confiabilidade e a segurança dos dados coletados. A ferramenta a ser utilizada para a coleta e gestão de dados será a plataforma *Otus* (https://site.otus-solutions.com.br/). O acesso à plataforma de dados será realizado por usuário e senha pessoais e intransferíveis, por membro da equipe, após devida delegação no estudo pelo pesquisador responsável. Os usuários da plataforma (equipe da pesquisa) terão permissões específicas correlatas à sua função e delegação no estudo.

Qualidade e segurança de dados

Diversos procedimentos serão empregados para garantir a segurança e a qualidade dos dados:

- Todos os pesquisadores participarão de sessão de treinamento antes do início do estudo a respeito de boas práticas clínicas e procedimentos do estudo, incluindo a coleta dos dados;
- Todos os processos relacionados à gestão dos dados estarão em conformidade com a Lei Geral de Proteção de Dados (LGPD; Lei nº 13.709, de 14 de agosto de 2018);
- O acesso ao banco de dados será protegido por senha individual e intransferível restrita à equipe de pesquisa delegada para o estudo;
- O processo de *backup* do banco de dados será feito de forma automatizada com periodicidade de 24 horas. A extração de dados para o software estatístico se dará de forma automatizada, com anonimização dos dados para processos de checagem de consistência de dados, ações de monitoria, desenvolvimento de variáveis derivadas e análises estatísticas;
- Limpezas de dados para identificação de inconsistências serão conduzidas periodicamente. Os pesquisadores serão notificados sobre as inconsistências para que providenciem correção;
- As entrevistas remotas serão gravadas e auditadas para verificação de consistência e adjudicação dos dados. Os arquivos de áudio serão armazenados de forma anonimizada em servidor com o mesmo sistema de segurança do banco de dados acima descrito. O acesso a estes arquivos ocorrerá de forma protegida por usuário e senha pessoais e intransferíveis pela equipe de pesquisa;
- Os pesquisadores principais revisarão mensalmente relatórios detalhados sobre triagem, inclusão, seguimento, consistências e completude dos dados e tomarão imediatamente ações para resolver eventuais problemas;
- Técnicas estatísticas para identificação de fraude serão realizadas ao longo do estudo.

Tamanho amostral

Considerando-se uma incidência de condição pós-COVID-19 de 20%,[4] estima-se um tamanho amostral de 1.540 participantes com histórico de COVID-19 para permitir a detecção de uma diferença maior ou igual a 0,05 utilidades do escore de qualidade de vida EQ-5D-3L (dentro da faixa de diferença clínica minimamente significativa)[22] entre pacientes com a condição pós-COVID-19 e participantes sem a condição pós-COVID-19, considerando um poder de 80%, um alfa bi-caudal de 0,05 e um desvio padrão do escore de utilidade de 0,28. Para lidar com potenciais incertezas relacionadas a parâmetros de qualidade de vida

pós-COVID-19 e, também, perda de poder devido à necessidade de ajuste de covariáveis, a amostra foi inflada em 10%. Dessa forma, o presente estudo de coorte recrutará 1.694 participantes.

Para o estudo caso-controle aninhado, um tamanho amostral de 123 participantes (63 casos de disfunção cognitiva pós-COVID-19 e 63 controles sem disfunção cognitiva pós-COVID-19) permitirá detectar uma diferença de médias com tamanho de efeito de 0,05 (considerado moderado de acordo com Cohen) nos escores de função cognitiva.

Análise estatística

As variáveis contínuas serão expressas como média e desvio-padrão ou como mediana e intervalo interquartil, enquanto as variáveis categóricas serão expressas como frequências absolutas e relativas. A normalidade dos dados será avaliada por inspeção de histogramas.

A comparação dos escores de utilidade do EQ-5D-3L (desfecho primário) entre pacientes com e sem condição pós-COVID-19 será realizada utilizando-se modelos lineares generalizados ajustados por idade, gênero, comorbidades, fragilidade e região de residência (Norte, Nordeste, Sudoeste, Centro-Oeste e Sul). Os resultados ajustados do escore de utilidade do EQ-5D-3L e dos domínios do questionário (mobilidade, autocuidado, atividades usuais, dor/desconforto e ansiedade/depressão) serão resumidos para cada grupo de comparação utilizando tendência central e medidas de dispersão, juntamente da diferença média ou mediana como medida do tamanho do efeito. A comparação de desfechos secundários entre pacientes com e sem condição pós-COVID-19 será realizada utilizando o mesmo modelo utilizado para o desfecho primário. A medida de efeito utilizada será diferença absoluta (para desfechos categóricos) e diferença média ou mediana (para desfechos contínuos).

A avaliação dos fatores associados com a condição pós-COVID será realizada com uso de modelos lineares generalizados considerando a distribuição de Poisson com variância robusta. Variáveis que apresentarem um valor $p < 0,20$ serão incluídas no modelo multivariável o qual utilizará o procedimento *forward*. O resultado da associação de variáveis com a condição pós-COVID será descrito através de risco relativo.

A comparação entre os escores de testes de domínios de cognição entre casos de disfunção cognitiva pós-COVID-19 e controles sem disfunção cognitiva pós-COVID-19 será

realizada com o uso de modelos lineares generalizados. A medida de efeito utilizada será diferença média ou mediana.

Um nível de significância estatística de 0,05 será considerado para todas as comparações. Intervalos de confiança de 95% serão descritos para todas as análises de medida de efeito, sem ajuste para multiplicidade. As análises serão conduzidas com utilização do software R em versão a ser detalhada no momento das análises.[35]

ÉTICA E BOAS PRÁTICAS CLÍNICAS

A pesquisa foi planejada e será realizada em conformidade com a Resolução 466 de 12 de dezembro de 2012 do Conselho Nacional de Saúde[36] e das Diretrizes de Boas Práticas Clínicas, Emenda 6 – Revisão 2 do *International Council for Harmonisation* (ICH),[37] além das normas preconizadas pela LGPD. A pesquisa será iniciada apenas após aprovação completa do protocolo e documentos correlatos pelo sistema CEP/CONEP. Esta pesquisa também segue as orientações para procedimentos em pesquisas com qualquer etapa em ambiente virtual, descritas na Carta Circular nº 1/2021 da CONEP de 03 de março de 2021.

Consentimento para participação

Seguindo as Diretrizes e Normas Regulamentadoras de Pesquisa Envolvendo Seres Humanos, estabelecidos pela Resolução 466/12 do Conselho Nacional de Saúde, um TCLE será apresentado aos participantes da pesquisa no momento do convite para participar no estudo. O TCLE fornecerá, em linguagem acessível e clara, as informações acerca dos objetivos, da metodologia, do processo de coleta e registro das informações do estudo e, ainda: explicitação dos possíveis desconfortos e riscos decorrentes da participação e suas medidas mitigatórias; explicação dos benefícios esperados dessa participação; garantia de plena liberdade ao participante de recusar-se a participar ou retirar seu consentimento em qualquer fase da pesquisa, sem penalização alguma; garantia de manutenção do sigilo e da privacidade dos participantes da pesquisa durante todas as fases da pesquisa; garantia de que o participante da pesquisa receberá uma via do TCLE; explicitação da garantia de ressarcimento e como serão cobertas as despesas tidas pelos participantes da pesquisa e dela decorrentes e explicitação da garantia de indenização diante de eventuais danos decorrentes da pesquisa.

Considerando a diversidade de gerações e suas preferências, facilidades ou limitações em relação ao uso de tecnologias, o processo de consentimento poderá ocorrer de forma independente, ou de forma assistida. No consentimento independente, o termo de consentimento livre e esclarecido digital (e-TCLE) será disponibilizado ao indivíduo interessado em participar do estudo, no momento do contato inicial, após a confirmação dos dados disponibilizados no cadastro de interesse. O e-TCLE será disponibilizado por link individual, por e-mail ou em mensagem de texto, conforme preferência do potencial participante. O potencial participante realizará a leitura do termo e registrará o seu consentimento de forma eletrônica. No processo assistido, ocorrerá no caso do participante manifestar que desejar auxílio ou esclarecimentos adicionais. Nesses casos, haverá a opção do indivíduo conversar com um dos pesquisadores por mensagem de texto ou telefone (o que o indivíduo preferir), para esclarecer as dúvidas ou o processo de registro eletrônico do consentimento. Caso o processo de consentimento seja realizado de forma presencial, o participante receberá o link do e-TCLE em seu e-mail e poderá sanar eventuais dúvidas presencialmente com o pesquisador.

O potencial participante da pesquisa terá tempo suficiente para ler o e-TCLE e a oportunidade de tirar dúvidas com o pesquisador. Após as explicações e antes de qualquer procedimento do estudo, o consentimento será registrado por meio de registro de aceite no e-TCLE. Após registro do consentimento, uma via do e-TCLE será disponibilizada imediatamente ao participante, por meio do seu endereço eletrônico (e-mail), ou mensagem de texto, conforme preferência do participante, podendo ser encaminhada uma via impressa pelos correios, posteriormente, caso o participante desejar. Na via do e-TCLE constará o nome do pesquisador que disponibilizou o e-TCLE e conduziu o processo de consentimento, bem como a data e hora do registro do consentimento do participante.

Os participantes que consentirem ser contatados para participação no estudo caso-controle, caso selecionados, só participarão desse subestudo caso consentirem de forma voluntária após leitura de e-TCLE específico para esse estudo, seguindo os mesmos procedimentos para registro do consentimento, descritos anteriormente.

Potenciais riscos e benefícios

Os riscos para o participante do estudo são mínimos, visto que o estudo é observacional e envolve apenas atividades de entrevista para coleta de dados. Em relação à coleta de dados, mesmo que a equipe seja treinada quanto às normas preconizadas pela Lei Geral de Proteção de Dados, há o risco de quebra da confidencialidade e privacidade dos dados, os quais são mitigados conforme descrito nos itens Gerenciamento de Dados e Confidencialidade neste protocolo. Ainda, existem os riscos relacionados ao desconforto ao responder a alguma questão relacionada à saúde. Nestes casos, o participante poderá optar por não responder. O tempo para realização da entrevista também pode ser considerado um desconforto.

Embora este estudo não tenha um benefício imediato aos participantes, ele poderá trazer contribuições no longo prazo, uma vez que os conhecimentos obtidos através de sua execução têm potencial para direcionar as políticas e esforços nacionais para prevenção, tratamento e recuperação dos pacientes afetados pela COVID-19.

Ao final da entrevista, todos os participantes receberão um relatório com os resultados individuais dos testes respondidos (**Anexo 2** – Modelo de relatório com resultados de testes respondidos) - e, para aqueles participantes em que os escores dos testes forem indicativos de presença de sintomas, serão orientados a apresentarem esse documento ao seu médico assistente. Importante reforçar que os testes aplicados não definem um diagnóstico. Os testes podem sinalizar sintomas e podem ser utilizados de forma complementar para auxiliar uma avaliação clínica que venha a ser realizada por profissional médico em eventual consulta realizada pelo participante. Saber essas informações é um direito do participante, dessa forma, não está tratado como benefício no TCLE.

Confidencialidade

Toda a informação pessoal do participante será sigilosa e somente a equipe do estudo terá acesso para evitar quebra de confidencialidade. Em nenhum momento, o nome do participante ou qualquer informação sobre a sua saúde será fornecida para qualquer pessoa que não seja da equipe do estudo e que realize atividade na qual seja necessário o acesso a essas informações. As informações serão confidenciais e utilizadas somente para fins desta

pesquisa. Todas as medidas cabíveis para evitar a quebra de sigilo da identidade do participante e confidencialidade serão executadas. Estas incluem acesso a documentos do estudo somente às pessoas da equipe de pesquisa e armazenamento de dados eletrônicos em uma base de dados com acesso seguro por meio de usuário e senha individuais somente para os pesquisadores e outros membros da equipe de pesquisa. Os resultados do estudo serão divulgados de forma agregada, para fins acadêmicos e científicos, sem a identificação de nenhum dado que revele a identidade dos participantes.

ORGANIZAÇÃO DO ESTUDO

Centro coordenador

O estudo será coordenado pela *INOVA Research,* departamento da *INOVA MEDICAL - SERVICOS DE SAÚDE E GESTÃO LTDA*, responsável pela operacionalização de estudos. O centro coordenador é responsável por dar suporte às operações necessárias para condução do estudo incluindo procedimentos éticos/regulatórios; treinamento, orientação e apoio aos pesquisadores delegados para garantir a adesão ao protocolo de pesquisa e às boas práticas clínicas; seguimento remoto centralizado dos participantes incluídos na pesquisa; monitoria e gerenciamento de dados; análise de resultados; disseminação dos resultados da pesquisa. A equipe do centro coordenador tem formação e experiência em pesquisa clínica, estatística e bioética.

Comitê diretivo

O Comitê Diretivo é responsável pela supervisão da execução do estudo, garantindo a integridade ética e altos padrões de qualidade metodológica. As atribuições específicas do comitê diretivo incluem: 1) monitorar o progresso do estudo de acordo com objetivos pré-estabelecidos; 2) supervisionar a qualidade dos métodos empregados na pesquisa; 3) revisar, em intervalos regulares, informações da literatura científica que possam demandar modificações nos procedimentos do estudo; 4) garantir que órgãos regulatórios relacionados à pesquisa clínica sejam notificados apropriadamente quando necessário; 5) aplicar as políticas de disseminação do estudo. Os membros do Comitê Diretivo são pesquisadores treinados e com experiência na elaboração e condução de estudos clínicos, bem como nas áreas de epidemiologia e pesquisa clínica.

Patrocinador

A *INOVA MEDICAL - SERVICOS DE SAÚDE E GESTÃO LTDA* possui a responsabilidade de instituição patrocinadora conforme descrito na RDC número 09/2015[38] e Diretrizes de Boas Práticas Clínicas, Emenda 6 – Revisão 2 do ICH.[37]

Apoio financeiro

Este estudo é financiado pela MERCK SHARP DOHME FARMACÊUTICA LTDA (MSD), inscrita no Cadastro Nacional de Pessoas Jurídicas sob o nº 03.560.974/0001-18, através do *Grant* de pesquisa *Merck Investigator Studies Program* (*MISP*#101021).

POLÍTICA DE DISSEMINAÇÃO

Plano de disseminação dos resultados

Os pesquisadores responsáveis esperam poder disseminar os resultados do presente estudo através de apresentações em congressos científicos e publicação em periódicos científicos com revisão por pares. A decisão a respeito de publicação dos dados do estudo, bem como do periódico alvo será de responsabilidade do comitê diretivo do estudo.

Os resultados também serão divulgados diretamente para os participantes, com sumário dos resultados em português, e em linguagem de fácil entendimento, sendo enviados aos participantes através de e-mail. Além disso, essas informações estarão disponíveis na página eletrônica utilizada para recrutamento (www.covidlonga.com.br). Esses resultados serão divulgados somente após a finalização do estudo.

Política de autoria

Para ser creditado como autor de artigo (s) resultantes do presente estudo, o pesquisador postulante precisa preencher todos os 4 critérios recomendados pelo *International Committee of Medical Journal Editors* (ICMJE)[39] listados abaixo:

- **Critério 1**: Contribuir substancialmente para pelo menos uma das seguinte atividades: concepção do estudo, desenho do estudo, coleta de dados, análise de dados ou interpretação dos dados;
- **Critério 2:** Participar da redação do manuscrito do estudo ou da revisão crítica do conteúdo intelectual;
- **Critério 3:** Aprovar a versão do manuscrito a ser submetido para publicação;
- **Critério 4:** Concordar em assumir (em conjunto com os outros autores) os créditos e a responsabilidade por todos os aspectos relacionados ao estudo, incluindo acurácia e integridade dos dados.

Política de compartilhamento de dados

Pedidos de compartilhamento de dados serão avaliados pelo comitê diretivo do estudo.

REFERÊNCIAS

1. Johns Hopkins University & Medicine (2022) Coronavirus Resource Center. https://coronavirus.jhu.edu/map.html.
2. Crook H, Raza S, Nowell J, et al. Long covid-mechanisms, risk factors, and management. BMJ 2021;374:n1648.
3. Michelen M, Manoharan L, Elkheir N, et al. Characterising long COVID: a living systematic review. BMJ Glob Health 2021;6(9):e005427.
4. World Health Organization. A clinical case definition of post COVID-19 condition by a Delphi consensus. 2021. WHO/2019-nCoV/Post_COVID-19_condition/Clinical_case_definition/2021.1
5. Global Burden of Disease Long COVID Collaborators. Estimated Global Proportions of Individuals With Persistent Fatigue, Cognitive, and Respiratory Symptom Clusters Following Symptomatic COVID-19 in 2020 and 2021. JAMA 2022;328(16):1604-1615.
6. O'Mahoney LL, Routen A, Gillies C, et al. The prevalence and long-term health effects of Long Covid among hospitalised and non-hospitalised populations: A systematic review and meta-analysis. EClinicalMedicine 2022;55:101762.
7. Chen C, Haupert SR, Zimmermann L, et al. Global Prevalence of Post-Coronavirus Disease 2019 (COVID-19) Condition or Long COVID: A Meta-Analysis and Systematic Review. J Infect Dis 2022;226(9):1593-1607.
8. Ballering AV, van Zon SKR, Olde Hartman TC, et al. Persistence of somatic symptoms after COVID-19 in the Netherlands: an observational cohort study. Lancet 2022;400(10350):452-461
9. Munblit D, TR Nicholson, DM Needham, et al. Studying the post-COVID-19 condition: research challenges, strategies, and importance of Core Outcome Set development. BMC Med 2022;20:50.
10. Tsuzuki S, Miyazato Y, Terada M, et al. Impact of long-COVID on health-related quality of life in Japanese COVID-19 patients. Health Qual Life Outcomes 2022;20(1):125.
11. Logue JK, Franko NM, McCulloch DJ, et al. Sequelae in Adults at 6 Months After COVID-19 Infection. JAMA Netw Open 2021;4(2):e210830.

12. Rosa RG, Cavalcanti AB, Azevedo LCP, et al. Association between acute disease severity and one-year quality of life among post-hospitalisation COVID-19 patients: Coalition VII prospective cohort study. Intensive Care Med 2023:1–12.
13. Brightling CE, Evans RA. Long COVID: which symptoms can be attributed to SARS-CoV-2 infection? Lancet 2022;400(10350):411-413.
14. Kovelis D, Segretti NO, Probst VS, Lareau SC, Brunetto AF, Pitta F. Validation of the Modified Pulmonary Functional Status and Dyspnea Questionnaire and the Medical Research Council scale for use in Brazilian patients with chronic obstructive pulmonary disease. J Bras Pneumol. 2008 Dec;34(12):1008-18.
15. Valderramas S, Camelier AA, Silva SA, et al. Reliability of the Brazilian Portuguese version of the fatigue severity scale and its correlation with pulmonary function, dyspnea, and functional capacity in patients with COPD. J Bras Pneumol. 2013;39(4):427-33.
16. Charlson ME, Pompei P, Ales KL, MacKenzie CR. A new method of classifying prognostic comorbidity in longitudinal studies: development and validation. J Chronic Dis 1987;40(5):373-383.
17. Church S, Rogers E, Rockwood K, Theou O. A scoping review of the Clinical Frailty Scale. BMC Geriatr 2020;20(1):393.
18. Working Group on the Clinical Characterisation and Management of COVID-19 infection. A minimal common outcome measure set for COVID-19 clinical research. Lancet Infect Dis 2020;20:e192-e7.
19. Cook SE, Marsiske M. The Use of the Modified Telephone Interview for Cognitive Status (TICS-M) in the Detection of Amnestic Mild Cognitive Impairment. J Geriatr Psychiatry Neurol 2009; 22(2): 103–109.
20. Santos M, MA Cintra, AL Monteiro, et al. Brazilian Valuation of EQ-5D-3L Health States: results from a saturation study. Med Decis Making 2016;36:253-63.
21. Santos M, AL Monteiro, B Santos. EQ-5D Brazilian population norms. Health Qual Life Outcomes 2021;19:162.
22. Coretti S, M Ruggeri, P McNamee. The minimum clinically important difference for EQ-5D index: a critical review. Expert Rev Pharmacoecon Outcomes Res 2014;14:221-33.
23. Santos RL, Virtuoso Jr JS. Reliability of the Brazilian version of the Scale of Instrumental Activities of Daily Living. Rev Bras Prom Saúde. 2008;21(4):290-6.

24. Minosso JS, Amendola F, Alvarenga MR, Oliveira MA. Validation of the Barthel Index in elderly patients attended in outpatient clinics, in Brazil. Acta Paul Enferm 2010;23(2):218–223.
25. Zigmond AS, Snaith RP. The hospital anxiety and depression scale. Acta Psychiatr Scand 1983;67(6):361-70.
26. Caiuby AV, SS Lacerda, MI Quintana, TS Torii, SB Andreoli. Cross-cultural adaptation of the Brazilian version of the Impact of Events Scale-Revised (IES-R). Cad Saude Publica 2012;28:597-603.
27. Thoresen S, Tambs K, Hussain A, Heir T, Johansen VA, Bisson JI. Brief measure of posttraumatic stress reactions: impact of event scale-6. Soc Psychiatry Psychiatr Epidemiol. 2010;45:405–12.
28. Pendlebury ST, Welch SJ, Cuthbertson FC, et al. Telephone assessment of cognition after transient ischemic attack and stroke: modified telephone interview of cognitive status and telephone Montreal Cognitive Assessment versus face-to-face Montreal Cognitive Assessment and neuropsychological battery. Stroke 2013;44(1):227-9.
29. Rosemberg SJ, Rayan JJ, Prifitera. Rey Auditory-Verbal Learning Test performance of patients with and without memory impairment. J Clin Psychol 1984;40(3):785-7.
30. Altmann RF, Ortiz KZ, Benfica TR, et al. Brief Montreal-Toulouse Language Assessment Battery: adaptation and content validity. Psicol Reflex Crit 2020;33(1):18.
31. Gil G, Magaldi RM, Busse AL, et al. Development of a word accentuation test for predicting cognitive performance in Portuguese-speaking populations. Arq Neuropsiquiatr 2019;77(8):560-567.
32. Fonseca R, Salles JF, Parente MAMP. Development and content validity of the brazilian brief neuropsychological assessment battery Neupsilin. Psychol Neurosci. 2008;1:55–62.
33. Zimmermann, N., Cardoso, C.O., Trentini, C.M., Grassi-Oliveira, R., Fonseca, R.P. (2015). Brazilian preliminary norms and investigation of age and education effects on the Modified Wisconsin Card Sorting Test, Stroop Color and Word test and Digit Span test in adults. Dementia & Neuropsychologia, 9(2), 120-127.
34. Godoy, V.P., Mattos, P., & Malloy-Diniz, L.F. (2011). Escala de Avaliação de Disfunções Executivas de Barkley – BDEFS. São Paulo, Brasil: Hogrefe.

35. R Development Core Team. R: A language and environment for statistical computing. Vienna: R Foundation for Statistical Computing; 2016. https://www.r-project.org
36. Conselho Nacional de Saúde (Brasil). Resolução n. 466, de 12 de dezembro de 2012. Brasília, DF, 2012.
37. International conference on harmonisation of technical requirements for registration of pharmaceuticals for human use. ICH Harmonised Tripartite Guideline. Guideline for good clinical practice. E6(R1). 2016.
38. Agência Nacional de Vigilância Sanitária (ANVISA). Resolução da diretoria colegiada - rdc nº 9, de 20 de fevereiro de 2015. Brasília, DF, 2015. http://antigo.anvisa.gov.br/documents/10181/3503972/%282%29RDC_09_2015_COMP.pdf/ee294d51-055f-4244-8e1a-b62f0c8e4f88
39. International Committee of Medical Journal Editors. Defining the role of authors and contributors. http://www.icmje.org/recommendations/browse/roles-and-responsibilities/defining-the-role-of-authors-and-contributors.html

Estudo COVID Longa Brasil

Anexo_1_Plano_de_recrutamento_e_divulgacao_v3_20_abr_2024

Incidência, fatores associados e impacto da condição pós-COVID-19 no Brasil durante a fase Ômicron: Estudo observacional

Plano de recrutamento e divulgação

Processo de Recrutamento e de consentimento no estudo

Esta pesquisa segue as orientações para procedimentos em pesquisas com qualquer etapa em ambiente virtual, descritas na Carta Circular nº 1/2021 da CONEP de 03 de março de 2021, uma vez que terá todas as suas etapas poderão ser desenvolvidas em ambiente virtual. O recrutamento de participantes será realizado de forma eletrônica, através de uma página na internet desenvolvida exclusivamente para a divulgação do estudo, na qual a pessoa interessada preencherá um formulário (cadastro) com informações básicas para que possa ser iniciado o contato pela equipe da pesquisa. Somente serão contatadas as pessoas que realizaram esse cadastro, uma vez que a realização do cadastro indica manifestação de interesse no estudo e autorização para que a pessoa seja contatada pela equipe. Adicionalmente, essa página já está disponível para acesso no endereço www.covidlonga.com.br, contudo a função de recebimento de informações através da mesma está ficou desabilitada (ou seja, dados fornecidos nos formulários não serão efetivamente enviados), com a funcionalidade passando a ser habilitada apenas após a aprovação para início do recrutamento pelo Comitê de Ética em Pesquisa.

A página foi desenvolvida em *Wordpress* e possui certificado SSL (*Secure Sockets Layer*), que consiste em um certificado digital que autentica a identidade de um site e possibilita uma conexão criptografada. É o mesmo tipo de certificado utilizado, por exemplo, em páginas com transações através de cartão de crédito. A página está hospedada em um servidor dedicado de hospedagem (o servidor contratado é dedicado apenas ao site em questão e contém apenas os dados referentes ao site e às respostas por ele recebidas); esse é um aspecto importante, uma vez que a maioria das páginas eletrônicas ficam hospedados em um ambiente compartilhado com outras organizações, o que conferiria maior vulnerabilidade às informações. Além disso, a hospedagem dessa página está em um servidor em território nacional, e a empresa responsável

(www.hostoo.com.br) é uma empresa nacional; dessa forma todos os entes envolvidos nesse processo estão sujeitos à Lei Geral de Proteção de Dados Pessoais (LGPD, lei nº 13.709/2018).

Fonte: www.covidlonga.com.br

Nessa página são apresentadas informações gerais sobre o estudo e do que consiste a sua participação, além de informações de contato (e-mail e telefônico) da equipe da pesquisa. Também é informado que se trata de um cadastro de interesse na pesquisa, a partir do qual a equipe entrará em contato para explicar no que consiste participar da pesquisa e que a pessoa só será participante se consentir participar de forma voluntária e por meio da assinatura do Termo de Consentimento Livre e Esclarecido (TCLE). Além disso, quando aprovado, será incluído na página um link para o acesso ao TCLE, possibilitando a leitura prévia do documento.

Os dados a serem coletados serão os seguintes:

- Nome completo (conforme documento de identificação);
- Pronome
- Idade em anos;
- Sexo atribuído ao nascer (havendo a possibilidade de não o informar);
- Estado onde mora;
- Cidade onde mora;
- E-mail (recomendado que seja de acesso frequente, ao menos uma vez por semana);
- Telefone com DDD;
- Como prefere que seja o contato inicial pela equipe da pesquisa (por aplicativo de mensagem de texto ou por ligação telefônica/chamada de voz);
- Turno de preferência para contato;
- Comentários adicionais (por exemplo, horário que prefere o contato inicial pela equipe de pesquisa), sendo esse campo opcional;
- Solicitação de confirmação de infecção prévia por COVID-19 após 01 de janeiro de 2022;
- Solicitação de confirmação de que há o aceite em ser contatado pela equipe da pesquisa.
- Como ficou sabendo sobre o estudo (campo de preenchimento opcional)

Ao final do cadastro constará a seguinte informação:

Está prevista a participação de 1.694 participantes nesta pesquisa. Os contatos pela equipe de pesquisa vão ocorrer em ordem de cadastro, a partir das informações disponibilizadas e considerando também a distribuição proporcional por estados e por idade dos participantes. Este cadastro também não garante a sua inclusão no estudo, a qual será efetivada somente a partir do contato por um pesquisador da equipe e do seu consentimento voluntário e expresso em participar.

Política de Dados

Todos os dados serão tratados de acordo com a Lei Geral de Proteção de Dados Pessoais do Brasil (LGPD - Lei nº 13.709, de 14 de agosto de 2018). Os dados aqui coletados serão utilizados estritamente pela equipe de pesquisa devidamente cadastrada junto à Inova Medical, patrocinador da pesquisa e somente para os fins de contato para participação na pesquisa.

O acesso às respostas será exclusivamente aos pesquisadores do estudo, devidamente treinados e expressamente comprometidos com o sigilo e confidencialidade dos dados. O cadastro será periodicamente (a cada 2 semanas) apagado, sendo mantido apenas as seguintes informações para fins de registro e auditoria: idade do participante, estado onde mora, sexo, data do registro e se houve ou não inclusão no estudo e o motivo para tal (podendo ser: não foi possível realizar contato após 10 tentativas em dias e horários diferentes respeitando as preferencias de contato, não aceitou participar, problemas logísticos na concretização da assinatura eletrônica do TCLE impediram a participação, distúrbios auditivos ou de fala impossibilitaram a participação, número de participantes para o estrato referido já foi atingido, outro[informar qual]). Além disso, a partir do website serão obtidas estatísticas de acesso à página (número de acessos por dia), de forma a monitorar o processo de recrutamento.

As etapas de divulgação e recrutamento que se referem ao contato inicial, convite para participar do estudo e condução do consentimento também poderão ser realizadas presencialmente, por recrutadores treinados e delegados pelo pesquisador responsável. Neste caso, a pessoa interessada em participar também fará cadastro de interesse na página eletônica do estudo.

Uma vez a pessoa cadastrada na página do estudo, será realizado contato pela equipe de pesquisa, a qual irá se apresentar, conferir os dados informados: sexo, idade, estado e cidade de residência, data do episódio de COVID-19 e como foi realizado o diagnóstico. Caso esses dados

estiverem de acordo com os critérios de elegibilidade para participar do estudo, será solicitado para o potencial participante confirmar o endereço de e-mail e o nome completo, e se procederá com o processo de consentimento. Será, nesse momento, solicitado que o participante leia o TCLE, sendo oferecido auxílio na leitura do TCLE pelo participante (caso a conversa esteja sendo realizada por mensagem, também será oferecida a possibilidade do pesquisador ligar para o participante). Será oferecida a possibilidade do pesquisador permanecer na ligação caso haja alguma dúvida, ou, se o potencial participante da pesquisa preferir, poderá ser definido um horário posterior para entrar em contato para realizar o esclarecimento das dúvidas, assim como se manter disponível para responder os questionamentos por aplicativo de mensagem de texto. Após o registro do consentimento no TCLE de forma eletrônica e confirmação de que o participante recebeu em seu e-mail a sua via do documento com o registro da data e hora do consentimento, bem como o nome do pesquisador de disponibilizou o TCLE e conduziu o processo quando assistido, serão iniciados os procedimentos da pesquisa. Uma via impressa do TCLE com o registro da data e hora do consentimento, bem como o nome do pesquisador que disponibilizou o TCLE e conduziu o processo quando assistido, também poderá ser enviada pelos correios, se o participante desejar.

Em todos os contatos telefônicos ou por videochamada será confirmado se o potencial participante está em um lugar fisicamente seguro para proceder com a ligação. Os procedimentos não serão realizados se o potencialmente participante estiver, por exemplo, caminhando ou dirigindo, ou então em qualquer outra situação. Caso a qualidade da ligação não estiver adequada conforme julgamento do pesquisador, o contato será interrompido e procedido posteriormente.

Indivíduos que foram contactados e não foram incluídos no estudo (ou seja, não assinaram TCLE) terão todos os registros ou dados coletados pelo estudo deletados.

Nos casos do recrutamento presencial, após explicação sobre o estudo, o TCLE será disponibilizado por email e a leitura e registro do consentimento ou recusa em participar ocorrerão nos mesmos moldes do processo remoto. Neste caso, o indivíduo poderá sanar as dúvidas com o recrutador, ou, se preferir, poderá concluir o preenchimento do TCLE em momento que achar mais conveniente, de forma assistida ou não. O recrutamento presencial seguirá os moldes de busca em domicílios, evitando quaisquer tipos de abordagem em locais públicos, como forma de garantir privacidade aos indivíduos convidados.

Divulgação da página eletrônica (site) do estudo

O estudo será divulgado através de envio de e-mails e de anúncios em mídias sociais (Facebook e Instagram), WhatsApp e google Adsense. Todas as mensagens de divulgação, além das apresentadas aqui, serão previamente submetidas ao CEP por meio de notificação.

As seguintes mensagens inicialmente serão utilizadas:

1) E-mails a sociedades médicas, serviços de saúde (hospitais e clínicas) e universidades (cursos da saúde e programas de pós-graduação) com contatos públicos.

Remetente: contato@covidlonga.com.br

Assunto: Estudo Covid Longa Brasil.

Mensagem no corpo do email:

Prezados,

Com o objetivo de avaliar o impacto de longo prazo da infecção pela COVID-19 estamos recrutando adultos que tiveram a doença durante o ano de 2022 e que não necessitaram hospitalização. Serão entrevistados 1.694 adultos, de todas as regiões brasileiras, por telefone ou videoconferência. O tempo estimado de participação é de aproximadamente 1 hora. O estudo e essa divulgação foram aprovados pelo CEP do Hospital Moinhos de Vento sob parecer 6.088.948 de 18 de abril de 2023.

O formulário para manifestação de interesse na participação e informações adicionais do estudo, estão disponíveis no site: www.covidlonga.com.br

Caso saiba de alguém que pode se interessar em participar do estudo, agradecemos se puder encaminhar essa mensagem ou divulgar o site www.covidlonga.com.br . Em caso de dúvidas, responda a esse e-mail ou entre em contato pelo WhatsApp (51) 9819-2778.

Agradecemos sua colaboração

Anexo: não se aplica

2) Mensagem para mídias sociais e google Adsense:

Mensagem 1:

Ajude a construir o conhecimento sobre as consequências da Covid-19.

Participe do estudo Covid Longa Brasil.

Saiba mais: www.covidlonga.com.br

Mensagem 2:

Você tem mais de 18 anos e teve Covid-19 em 2022?

Participe da pesquisa Covid Longa Brasil.

Saiba mais: www.covidlonga.com.br

Mensagem 3:

Você ou algum conhecido teve Covid-19 em 2022?

Conheça o estudo Covid Longa Brasil.

Saiba mais: www.covidlonga.com.br

Mensagem 4:

Você tem mais de 18 anos e teve Covid-19 em 2022?

Ajude-nos com 1 hora do seu tempo e participe da pesquisa Covid Longa Brasil. Saiba mais: www.covidlonga.com.br

Mensagem 5:

"Teve Covid-19 em 2022? Participe de nossa pesquisa e ajude-nos a entender os efeitos a longo prazo da doença. Sua experiência é fundamental!"

Mensagem 6:

"Contraiu Covid-19 em 2022? Sua contribuição para nossa pesquisa pode ajudar a melhorar a compreensão e o tratamento dos efeitos de longo prazo da doença. Junte-se a nós!"

Mensagem 7:

"Se você é um adulto que contraiu Covid-19 em 2022, estamos à procura de participantes para um estudo sobre Covid Longa. Participe e ajude a esclarecer os efeitos duradouros desta doença!"

Mensagem 8:

"Se você teve Covid-19 em 2022, convidamos você a participar de uma pesquisa sobre os efeitos a longo prazo da doença. Ajude-nos a avançar a ciência e a saúde coletiva!"

3) Mensagens de WhatsApp a serem divulgadas para profissionais e pesquisadores envolvidos em iniciativas de Covid-19

O estudo Covid Longa Brasil irá entrevistar 1.694 adultos que tiveram Covid-19 a partir de 1º de janeiro de 2022. Se você ou algum familiar teve a doença, podem colaborar com o maior entendimento das consequências a longo prazo do Covid-19. Saiba mais: www.covidlonga.com.br

Novas apresentações que venham a ser empregadas além dessas, serão sempre previamente encaminhadas ao CEP por meio de notificação.

Uso de aplicativo de mensagem de texto

Considerando que o WhatsApp é o aplicativo de mensagem de texto presente em mais de 99% dos smartphones no Brasil (Fonte: Panorama de Mensageria no Brasil – Mobile Time/Opinion Box Agosto 2022) e, ainda, que possui mecanismos de segurança para o tráfego de informações sensíveis, toda a comunicação com o participante que não ocorra diretamente por ligação telefônica ou e-mail, ocorrerá apenas por meio do aplicativo WhatsApp. Para tanto o estudo dispõe de uma conta comercial na modalidade *WhatsApp Business* e utiliza todos os níveis de segurança recomendados pela desenvolvedora Meta, como por exemplo a verificação da conta em duas etapas e controles internos de autorização de uso da conta. Em relação ao controle interno de uso da conta, as pessoas autorizadas a usar a conta são os pesquisadores que possuem como função o contato com o participante, estarão delegadas em documento do estudo e expressamente comprometidas com o sigilo e confidencialidade dos dados. Pesquisadores que não foram delegados para o contato com o participante não terão acesso à conta. Para possibilitar esse controle, a conta de WhatsApp para divulgação do estudo não será a mesma utilizada para comunicação com os participantes por ligação telefônica. Por fim, toda a comunicação com o participante é realizada diretamente com um pesquisador treinado que utilizará manualmente textos padronizados para garantir a reprodutibilidade da informação. O estudo não utilizará ferramentas de automatização da comunicação individual tais como *chatbots*.

Reembolsos

Caso o potencial participante tenha qualquer custo com pacote de dados de internet, ligações ou mensagens (SMS), será ressarcido ao participante. Os ressarcimentos serão feitos através de PIX. Os seguintes valores serão considerados como padrão de ressarcimento caso o potencial participante refira que os custos não são cobertos pelo seu plano atual de telefonia ou de internet:

- SMS enviado: R$ 0,40
- Minuto de ligação realizada (por rede de telefonia celular): R$ 1,00
- Minuto de ligação realizada (por rede de telefonia fixa): R$ 0,30

- Minuto de ligação recebida ou realizada por meio de internet (sem vídeo): R$ 0,10 a cada 10 minutos (considera-se R$ 9,00 o custo de 1GB de dados, com 1GB sendo consumido a cada 15 horas de ligação)
- Minuto de videochamada recebida ou realizada por meio de internet: R$ 0,30 (considera-se R$ 9,00 o custo de 1GB de dados, com 1GB sendo consumido a cada 30 minutos de videochamada)

Valores adicionais de reembolso serão praticados mediante confirmação de gastos; valores de reembolso de até R$ 50,00 serão realizados baseados na presunção de veracidade do relato do potencial participante, enquanto para valores adicionais serão solicitadas comprovações (por exemplo, danos no celular durante a participação no estudo). Os reembolsos serão aplicáveis a partir do contato inicial pela equipe da pesquisa, independente da participação no estudo.

Os valores monetários não serão divulgados antecipadamente para o participante para não induzir participação devido a ressarcimento financeiro. Após a participação no estudo ou subestudo (ou no recrutamento no caso de não-inclusão) será questionado ao indivíduo: “O Sr(a) teve algum custo durante esse estudo que necessite reembolso como, por exemplo, custos com telefonia e internet?” No caso afirmativo será questionado que tipo de recurso foi utilizado e quantificado conforme os registros de contato. Para receber o reembolso o participante precisa fornecer por escrito a chave PIX, sendo de sua responsabilidade o fornecimento da informação. Caso o participante não use esse recurso para transações financeiras, serão buscados outros meios para o ressarcimento tais como depósito bancário, disponibilização de cartão para saque de valor pré-fixado.

Estudo COVID Longa Brasil

Anexo_2_Modelo_relatorio_resultados_testes_v2_31_jul_2023

Incidência, fatores associados e impacto da condição pós-COVID-19 no Brasil durante a fase Ômicron: Estudo observacional

Data da avaliação: <incluir data>

Data do episódio índice de COVID-19: <incluir data>

Entrevistador responsável pela avaliação: <incluir nome do entrevistador>

Pesquisador responsável: Dr. Maicon Falavigna. CRM-RS 31.743.

Agradecemos sua participação no Estudo COVID Longa Brasil. Nesse relatório compartilhamos os resultados dos testes realizados durante a sua entrevista. Você também receberá por email o acesso aos resultados gerais do Estudo COVID Longa Brasil, quando estiverem disponíveis; nossa expectativa é que os resultados sejam divulgados no primeiro semestre de 2024.

É importante esclarecer que a entrevista realizada e os dados aqui presentes no relatório não substituem a apropriada avaliação clínica por consulta médica. Apesar de serem questionários validados e aceitos tanto na prática clínica como em pesquisa em saúde, os testes realizados, em geral, servem como rastreamento de sintomas, não sendo por si só suficientes para definir diagnósticos ou sugerir condutas específicas. Além disso, esses testes não são específicos para COVID, assim, não é possível definir se houve ou não relação direta com o episódio de COVID.

Os testes realizados que possuem informações conhecidas na literatura para uma possível interpretação, assim como os seus resultados e interpretação desses resultados são apresentados a seguir:

HADS (Escala hospitalar de ansiedade e depressão - *Hospital Anxiety and Depression Scale*)

Consiste em um questionário para avaliar potenciais sintomas de ansiedade e depressão. Apesar de inicialmente ter sido desenvolvida para avaliar indivíduos hospitalizados, atualmente é bastante utilizada para avaliar indivíduos no contexto ambulatorial e por telefone. A escala HADS é dividida em duas subescalas: HADS-A (para avaliar sintomas de ansiedade) e HADS-D (para avaliar sintomas de depressão). Escore igual ou superior a 7, em cada uma das escalas, indica presença de sintomas compatíveis com ansiedade e com depressão.

Resultados:

HADS-A (ansiedade): Escore <resultado do escore>

Interpretação:

<Texto caso o valor seja < 7: O resultado está dentro da normalidade, ou seja, não foram detectados sintomas sugestivos de ansiedade.>

<Texto caso o valor seja ≥ 7: O resultado está acima do ponto de corte definido para o questionário. Contudo esse teste é apenas de rastreamento e não significa que tenha algum problema relacionado a ansiedade. Sugerimos apresentar essa informação ao seu médico-assistente em consulta de rotina.>

HADS-D (ansiedade): Escore < resultado do escore>

Interpretação:

<Texto caso o valor seja < 7: O resultado está dentro da normalidade, ou seja, não foram detectados sintomas sugestivos de depressão.>

<Texto caso o valor seja ≥ 7: O resultado está acima do ponto de corte definido para o questionário. Contudo esse teste é apenas de rastreamento e não significa que tenha algum problema relacionado a ansiedade. Sugerimos apresentar essa informação ao seu médico-assistente em consulta de rotina.>

II – IES-6 (Escala de Impacto do Evento-6 itens - *Impact of Events Scale-6*)

O transtorno do estresse pós-traumático é definido como um conjunto de sintomas associados a um evento traumático. Esses eventos são bastante diversos, podendo ser relacionados desde acidentes até catástrofes naturais. Eventos em saúde como hospitalização, diagnóstico de câncer, e mesmo COVID-19 podem ser eventos que desencadeiam o transtorno do estresse pós-traumático. A Escala do Impacto do Evento - **6 itens**(IES-**6**) tem sido referida como o instrumento de rastreamento da sintomatologia do transtorno do estresse pós-traumático. Resultados acima de 10 sugerem sintomas de estresse pós-traumático.

Resultados:

IES-6: Escore <resultado do escore>

Interpretação:

<Texto caso o valor seja < 10: O resultado está dentro da normalidade, ou seja, não foram detectados sintomas sugestivos de estresse pós traumático.

Texto caso o valor seja ≥ 10: O resultado está acima do ponto de corte definido para o questionário. Contudo esse teste é apenas de rastreamento e não significa que tenha algum problema relacionado a estresse pós-traumático. Sugerimos apresentar essa informação ao seu médico-assistente em consulta de rotina.>

TICS-M - Entrevista telefônica para avaliação do estado cognitivo - modificada

O TICS-M consiste em um instrumento utilizado no rastreamento de comprometimento cognitivo. Avalia fatores como memória, atenção e raciocínio. O escore vai de 0 a 39 pontos, com valores iguais ou maiores do que 31 pontos sendo considerados normais.

TICS-M: Escore <resultado do escore>

Interpretação:

<Texto caso o valor seja maior do que 31: O resultado está dentro da normalidade, ou seja, não parece haver nenhum comprometimento cognitivo.>

<Texto caso o valor esteja igual ou menor do que 31: O resultado é compatível com algum comprometimento cognitivo. O teste pode sofrer diversas influências externas como cansaço ou exercendo diferentes tarefas ao respondê-lo, e até mesmo qualidade da ligação telefônica, então não significa que haja algum problema necessariamente. Sugerimos avaliar os resultados com seu médico assistente o qual poderá avaliar melhor se há problemas relacionados com memória, atenção, raciocínio, entre outros, que podem impactar em sua qualidade de vida.>

Índice de Barthel

Consiste em uma escala que avalia atividades da vida diária, com objetivo de avaliar o grau de assistência necessária para as atividades do dia a dia. O escore vai de 0 a 100. 100 é considerado sem dependência funcional, 91 a 99 significa dependência leve, 61 a 90 dependência moderada, 21 a 60 dependência grave, e 0 a 20 indica total dependência funcional.

Resultado:

Escore antes do episódio de COVID-19: <resultado do escore>

Escore atual: <resultado do escore>

Interpretação:

<Se atual igual a 100: Seu resultado indica ausência de dependência funcional.>

<Se atual inferior a 100: Seu resultado indica certo grau de dependência funcional, tendo dependência no(s) seguinte(s) domínio(s): (adicionar domínios ao qual há algum grau de dependência).

<Se escore atual é menor do que o escore pré-Covid, adicionar: "Seu grau de dependência atual é maior do que o grau de dependência antes da Covid-19". Sugerimos que você converse com seu médico para ver se há alternativas que possam melhorar sua qualidade de vida.>

AIVD – Escala de atividades instrumentais de vida diária de Lawton e Brody

Semelhantemente ao Índice de Barthel, a AIVD consiste em uma escala que avalia atividades da vida diária, contudo enquanto o Barthel avalia predominantemente domínios físicos, a AIVD avalia atividades instrumentais do dia a dia que inclui desde utilizar o telefone a cuidar de suas finanças. O escore vai de 0 (baixa funcionalidade, dependente) a 8 (alta funcionalidade, independente).

Resultado:

Escore antes do episódio de COVID-19: <resultado do escore>

Escore atual: <resultado do escore>

Interpretação:

<Se atual igual a 8>: Seu resultado indica ausência de dependência instrumental.

<Se atual inferior a 8>: Seu resultado indica certo grau de dependência instrumental, tendo dependência no(s) seguinte(s) domínio(s): (adicionar domínios ao qual há algum grau de dependência).

<Se escore atual é menor do que o escore pré-Covid, adicionar: "Seu grau de dependência atual é maior do que o grau de dependência antes da Covid-19". Sugerimos que você converse com seu médico para ver se há alternativas que possam melhorar sua qualidade de vida.

Tabagismo

De acordo com a organização mundial de saúde, o tabaco é responsável por mais de 8 milhões de morte anualmente, em especial complicações pulmonares, cardiovasculares (infarto e isquemias cerebrais) e câncer.

Resultado: De acordo com suas respostas você (é/não é) fumante atualmente

<Se não tabagista> O manter-se longe do cigarro é muito importante para manter uma vida saudável, continue assim.

<Se tabagista> É importante avaliar com seu médico alternativas para reduzir e, de preferência, parar de fumar. Sabemos que parar de fumar não é uma ação simples, mas atualmente há diferentes tratamentos que podem auxiliar a ter maior sucesso; um médico poderá auxiliar a definir uma melhor estratégia para você.

Consumo de álcool

De acordo com a Organização Mundial de Saúde, o consumo de álcool é responsável por aproximadamente de 3 milhões de mortes anualmente. O álcool está bastante associado a acidentes, violência, distúrbios psiquiátricos, problemas hepáticos e digestivos e eventos cardiovasculares.

Resultado: Seu consumo semanal estimado é de xx gramas de álcool.

Segundo a OMS, não há um grau seguro de consumo de álcool, com o mais seguro sendo evitar o seu consumo. As diretrizes americanas classificam como um consumo moderado sendo equivalente a até 2 drinques (20g de álcool) por dia para homens e 1 drinque (10g de álcool) por dia para mulheres; lembrando que certas pessoas como gestantes, lactentes e quem possui problemas do fígado não devem beber. Para comparação, uma lata de 350ml de cerveja, um cálice de 125ml de vinho tinto e uma dose de 45ml de destilado possuem aproximadamente 16g de álcool cada. Essa diferença entre os sexos é devido às mulheres terem menor quantidade da enzima responsável por metabolizar o álcool.

<Se não consome álcool) Sua decisão de não consumir álcool é a mais acertada; mantenha-se assim>.

<Se consome <140g/semana homem ou <70g/semana mulher>: Seu consumo pode ser considerado moderado. O quanto beber é uma escolha pessoal, mas saiba que beber não é livre de risco. O quanto menos beber, menor é o risco de algum dano decorrente do consumo de álcool.

<Se consome >140g/semana homem ou >70g/semana mulher>: seu consumo de álcool é considerado elevado. É importante reduzir o seu consumo. Converse com seu médico sobre esse assunto.

Considerações adicionais

Esse relatório consiste em retorno dos testes realizados no estudo COVID Longa Brasil e não substitui avaliação médica. Os testes aplicados possuem limitações em sua sensibilidade e especificidade, não possuindo função diagnóstica. Todos os achados devem ser contextualizados com a avaliação clínica realizada por profissional de saúde.

<a ser colocados comentários adicionais conforme avaliação realizada>

No caso de dúvidas, entrar em contato com a equipe do estudo através do telefone (51) 9819-2778 ou pelo email contato@covidlonga.com.br.

Dr. Maicon Falavigna CRM-RS 31.743 é responsável por este relatório.

Assinatura do médico responsável pelo relatório: <xxxxxxxxxxxxxx>

Data: <data do relatório>

Os testes cognitivos conduzidos no subestudo de avaliação cognitiva serão realizados por neuropsicólogos e os resultados serão descritos por estes profissionais e revisados pelo pesquisador responsável. Em cada laudo, constará a seguinte observação: Os testes podem sofrer diversas influências externas como cansaço ou exercendo diferentes tarefas ao respondê-lo, e até mesmo qualidade da ligação telefônica, então não significa que haja algum problema necessariamente. Sugerimos avaliar os resultados com seu médico assistente o qual poderá avaliar melhor se há problemas relacionados com memória, atenção, raciocínio, entre outros, que podem impactar em sua qualidade de vida.

TERMO DE CONSENTIMENTO LIVRE E ESCLARECIDO

Versão 2 de 31 de julho de 2023

Título: Incidência, fatores associados e impacto da condição pós-COVID-19 no Brasil durante a fase Ômicron: Estudo observacional – ***COVID Longa Brasil***

Pesquisador Responsável: Maicon Falavigna – CRM-RS 31.743

Patrocinador: INOVA MEDICAL - SERVICOS DE SAÚDE E GESTÃO LTDA

Local de realização: Brasil

Você está sendo convidado(a) a participar de forma voluntária da pesquisa "Incidência, fatores associados e impacto da condição pós-COVID-19 no Brasil durante a fase Ômicron: Estudo observacional", a pesquisa *COVID Longa Brasil*, pois manifestou interesse em participar por meio da página eletrônica de divulgação da pesquisa (www.covidlonga.com.br). Esta pesquisa está sendo realizada em todo o Brasil pela Inova Medical, financiada pela Merck Sharp Dohme (MSD) Farmacêutica LTDA.

Antes de decidir pela participação na pesquisa, é importante que você entenda por que esta pesquisa está sendo realizada, todos os procedimentos envolvidos, os possíveis benefícios, riscos e desconfortos que serão descritos e explicados a seguir. Participar ou não participar dessa pesquisa não influenciará em quaisquer tipos de cuidados de saúde que você esteja realizando atualmente ou que realizar no futuro.

A qualquer momento, antes, durante e depois da pesquisa, você poderá solicitar maiores esclarecimentos, recusar ou desistir de participar. Em todos esses casos você não será prejudicado, penalizado ou responsabilizado de nenhuma forma.

Após ler este termo e ter suas dúvidas esclarecidas sobre a pesquisa, você precisará registar ao final deste documento digital se consente ou não participar da pesquisa. **Seu consentimento para participar da pesquisa de forma voluntária só ocorrerá se, ao final da leitura deste documento, você assinalar a opção "SIM, consinto participar da pesquisa". Se você não quiser participar da pesquisa, deve assinalar a opção "NÃO quero participar da pesquisa". Independente do que você decidir, uma via será enviada para seu endereço eletrônico (e-mail), com o registro de sua decisão, para que possa consultá-la sempre que necessário.** Caso queira, enviaremos uma cópia física do documento pelos correios.

Esta pesquisa está sendo realizada para conhecer os efeitos pós-COVID-19 na qualidade de vida relacionada à saúde em adultos que tiveram COVID-19 durante a disseminação da variante Ômicron no Brasil.

A pesquisa será realizada com 1.694 adultos residentes em qualquer região do Brasil, que tiveram COVID-19 com presença de sintomas e confirmada pelo teste RT-PCR, teste rápido ou auto-teste do antígeno (testes realizados por coleta de secreção do nariz e/ou da garganta com um cotonete), a partir de 01 de janeiro de 2022, mas que os sintomas e confirmação do teste tenham iniciado pelo menos 3 meses antes da sua participação na pesquisa (ou seja, 3 meses antes da data atual, caso você consentir participar).

Se você aceitar participar, os procedimentos da pesquisa envolvem a realização da confirmação de sua idade igual ou maior do que 18 anos, residência no Brasil, do teste positivo para COVID-19 após 01 de janeiro de 2022 e há pelo menos 3 meses anteriores à data atual, e

de ter apresentado sintomas quando estava com COVID-19 (por exemplo: febre, coriza, tosse, dor de garganta, entre outros). A confirmação do teste RT-PCR poderá ser pela leitura ou envio do resultado do teste (laudo), pelo auto-relato da realização e resultado do teste de antígeno e data correspondente, ou por acesso ao ConecteSUS, realizado por você. Esse procedimento levará cerca de 5 minutos. Se você não apresentar essas características, você não poderá seguir na pesquisa e nenhuma informação adicional será solicitada. Se você seguir na pesquisa, faremos perguntas sobre sua condição geral de vida e de saúde antes e depois de você ter tido COVID-19 e perguntas referentes a testes específicos que avaliam condições de saúde físicas e mentais. Esse procedimento será realizado por telefone ou videochamada, levará cerca de 50 minutos e será realizado em horário que for mais conveniente para você, considerando a recomendação de que esteja em um local que se sinta confortável e seguro para responder às perguntas da pesquisa. Ao final da entrevista, você receberá por e-mail o resultado dos testes aplicados durante a entrevista com o significado e orientações referentes aos resultados.

Além disso, entre os 1.694 participantes, 126 (7,5% do total) serão convidados a participar de um subestudo, para entender o impacto da COVID-19 na cognição (memória e atenção). Se você for selecionado para essa avaliação, e consentir ser contatado para participar, será convidado a ler um termo de consentimento específico e responder questões adicionais, por meio de videochamada, na qual um profissional habilitado aplicará testes para a avaliação da memória e atenção.

Todos esses procedimentos serão realizados por pesquisadores treinados e comprometidos com o sigilo das informações. As ligações telefônicas e/ou videochamadas serão gravadas, para finalidade exclusiva de auditoria e revisão dos dados, estando o acesso limitado aos pesquisadores envolvidos.

Os riscos em participar da pesquisa são mínimos e relacionados ao desconforto em ter que responder algumas das perguntas e o tempo dedicado à entrevista. Esta pesquisa não tem benefícios diretos para você. Os benefícios da pesquisa são indiretos, ou seja, os resultados da pesquisa reverterão em conhecimentos que podem esclarecer o impacto da COVID-19 na população brasileira.

As informações relacionadas à pesquisa poderão ser conhecidas por pessoas autorizadas como autoridades regulatórias ou sanitárias. Em razão dos procedimentos do estudo serem realizados por telefone, será necessário manter um cadastro com seu nome completo e contatos, para garantir a correta identificação durante os procedimentos da pesquisa. Essas informações serão de acesso exclusivo aos pesquisadores que realizam os contatos telefônicos e ficarão registradas em formulários separados dos demais dados que você fornecerá. Os seus dados pessoais não serão compartilhados e não estarão acessíveis para a Merck do Brasil, financiadora deste estudo. Todas as suas informações serão tratadas de forma confidencial e sigilosa pelos pesquisadores do estudo.

Você não terá quaisquer despesas para participar dessa pesquisa e você não receberá qualquer valor em dinheiro pela participação. Se ocorrer uma despesa por causa da participação da pesquisa (como, por exemplo, despesas de telefonia ou internet), o pesquisador responsável garante o ressarcimento dessa despesa. Em caso de danos comprovadamente decorrentes de sua participação na pesquisa, você terá direito a indenização.

Os resultados obtidos com esta pesquisa são para fins científicos. Você terá a garantia de que quando os resultados forem publicados, não aparecerá seu nome e não será possível

sua identificação em nenhuma forma. Quando os resultados forem publicados, você será avisado pela forma que for mais conveniente para você (telefone, e-mail ou carta) e você poderá acessar as informações na página eletrônica de divulgação da pesquisa (www.covidlonga.com.br).

Em caso de dúvidas sobre a pesquisa, você poderá entrar em contato com o pesquisador responsável Maicon Falavigna, (51) 9819-2778, no endereço Avenida General Flores da Cunha, 1050 sala 704, Cachoeirinha, Rio Grande do Sul, de segunda a sexta das 8:00 às 18:00 ou a qualquer momento pelo e-mail contato@covidlonga.com.br. Esta pesquisa foi analisada e aprovada pelo Comitê de Ética em Pesquisa (CEP) do Hospital Moinhos de Vento. O CEP é responsável pela avaliação e acompanhamento dos aspectos éticos de todas as pesquisas envolvendo seres humanos, visando garantir a dignidade, os direitos, a segurança e o bem-estar dos participantes de pesquisas. Caso você tenha dúvidas e/ou perguntas sobre seus direitos como participante desta pesquisa ou se estiver insatisfeito com a maneira como a pesquisa está sendo realizada, entre em contato com o Comitê de Ética em Pesquisa (CEP) do Hospital Moinhos de Vento, situado na Rua Ramiro Barcelos, 910, 4º andar, telefone 51-33143537, de segunda a sexta-feira, das 08:00 às 12:00 e das 13:00 às 18:00hs.

Consentimento do participante

A seguir, ao assinalar a opção "SIM, eu consinto participar da pesquisa", significa que você consente em participar como voluntário(a) desta pesquisa. Você confirma que foi devidamente informado(a) e esclarecido(a) sobre o objetivo desta pesquisa, que você leu ou que foi lido para você os procedimentos nela envolvidos, assim como os possíveis riscos e benefícios decorrentes da sua participação e que você sanou todas as suas dúvidas. Você confirma que foi garantido que você pode se recusar a participar e retirar o seu consentimento a qualquer momento, sem que isto cause qualquer prejuízo, penalidade ou responsabilidade a você ou sua família. Você autoriza a divulgação dos dados obtidos nesta pesquisa mantendo em sigilo a sua identidade. Você confirma que o pesquisador que conduziu o consentimento para participar nesta pesquisa garantiu que você receberá uma via deste documento com todas as páginas rubricadas e assinadas por você e pelo pesquisador que aplicou este termo.

Se você **não quiser participar da pesquisa**, você deve assinalar a opção "NÃO quero participar da pesquisa". Após isso, você não será mais contatado.

Você consente participar da pesquisa?

( ) SIM, consinto participar de forma voluntária da pesquisa.

( ) NÃO quero participar da pesquisa.

>>Se potencial participante selecionou "NÃO quero participar da pesquisa" aparecerá a seguinte mensagem:

Você decidiu NÃO participar da pesquisa.
Para confirmar essa decisão, por gentileza, clique em FINALIZAR.
Se deseja corrigir essa decisão, clique em VOLTAR.

>>Se potencial participante selecionou "FINALIZAR" o processo é encerrado e receberá por email a via do e-TCLE preenchida com essa decisão, contendo seu nome, data e hora que registrou a decisão, bem como o nome de quem disponibilizou e aplicou o e-TCLE.

>>Se potencial participante selecionou "SIM, consinto participar de forma voluntária da pesquisa." Segue para o consentimento sobre o convite para participar do subestudo.

Em relação ao convite para participar do **subestudo de avaliação da cognição (memória e atenção)**, caso você for selecionado(a):

( ) SIM, aceito ser convidado(a) para participar do subestudo, caso eu for selecionado(a).

( ) NÃO aceito ser convidado(a) para participar do subestudo, caso eu for selecionado(a).

>>Se potencial participante selecionou "SIM, consinto participar de forma voluntária da pesquisa." e ( ) SIM, aceito ser convidado(a) para participar do subestudo, caso eu for selecionado(a), aparecerá a seguinte mensagem:

> **Você consentiu participar da pesquisa e ser convidado(a) para participar do subestudo, caso selecionado(a).**
> *Para confirmar essa decisão, por gentileza, clique em FINALIZAR.*
> *Se deseja corrigir essa decisão, clique em VOLTAR.*

>>Ao selecionar "FINALIZAR" o processo de consentimento é concluído e o participante receberá por email a via do e-TCLE preenchida com essa decisão, contendo seu nome, data e hora que registrou a decisão, bem como o nome de quem disponibilizou e aplicou o e-TCLE.

>>Se potencial participante selecionou "SIM, consinto participar de forma voluntária da pesquisa." e ( ) NÃO aceito ser convidado(a) para participar do subestudo, caso eu for selecionado(a), aparecerá a seguinte mensagem:

> **Você consentiu participar da pesquisa, mas NÃO consentiu ser convidado(a) para participar do subestudo, caso selecionado(a).**
> *Para confirmar essa decisão, por gentileza, clique em FINALIZAR.*
> *Se deseja corrigir essa decisão, clique em VOLTAR.*

>> Ao selecionar "FINALIZAR" o processo de consentimento é concluído e o participante receberá por email a via do e-TCLE preenchida com essa decisão, contendo seu nome, data e hora que registrou a decisão, bem como o nome de quem disponibilizou e aplicou o e-TCLE.

Termo de Consentimento Livre e Esclarecido v.2 de 31 de julho de 2023.
Nome do participante: gerado automaticamente pelo sistema.
Data e hora do registro do consentimento pelo participante: gerado automaticamente pelo sistema.

Declaração do(a) pesquisador(a). Declaro que obtive de forma apropriada e voluntária o Consentimento Livre e Esclarecido deste participante para a participação nesta pesquisa. Declaro ainda, que me comprometo a cumprir o que está descrito neste termo.

Nome do pesquisador que disponibilizou e aplicou o e-TCLE: preenchido automaticamente pelo sistema.

>> OBS: as descrições em itálico são scripts sobre o processo do e-TCLE, não aparecem na via final do termo.

TERMO DE CONSENTIMENTO LIVRE E ESCLARECIDO

Versão 2 de 31 de julho de 2023

Título: Incidência, fatores associados e impacto da condição pós-COVID-19 no Brasil durante a fase Ômicron: Estudo observacional – Estudo COVID Longa Brasil ***/ Estudo para avaliação da cognição***

Pesquisador Responsável: Maicon Falavigna – CRM-RS 31.743

Patrocinador: INOVA MEDICAL - SERVICOS DE SAÚDE E GESTÃO LTDA

Local de realização: Brasil

Você está sendo convidado(a) a participar de forma voluntária do subestudo, que chamaremos de estudo para avaliação da cognição que faz parte da pesquisa "Incidência, fatores associados e impacto da condição pós-COVID-19 no Brasil durante a fase Ômicron: Estudo observacional – Estudo COVID Longa Brasil", pois você aceitou que a equipe da pesquisa entrasse contato, caso você fosse selecionado para participar. O estudo também está sendo realizado em todo o Brasil pela Inova Medical, financiada pela Merck Sharp Dohme (MSD) Farmacêutica LTDA

Antes de decidir pela participação no estudo, é importante que você entenda por que ele está sendo realizado, todos os procedimentos envolvidos, os possíveis benefícios, riscos e desconfortos que serão descritos e explicados a seguir. Participar ou não participar desse estudo não influenciará em quaisquer tipos de cuidados de saúde que você esteja realizando atualmente ou que realizar no futuro.

A qualquer momento, antes, durante e depois do estudo, você poderá solicitar maiores esclarecimentos, recusar ou desistir de participar. Em todos esses casos você não será prejudicado, penalizado ou responsabilizado de nenhuma forma.

Após ler este termo e ter suas dúvidas esclarecidas sobre a pesquisa, você precisará registar ao final deste documento digital se consente ou não participar do estudo. Seu consentimento para participar do estudo de forma voluntária só ocorrerá se, ao final da leitura deste documento, você assinalar a opção "SIM, consinto participar do estudo". Se você não quiser participar do estudo, deve assinalar a opção "NÃO quero participar do estudo". Independente do que você decidir, uma via será enviada para seu endereço eletrônico (e-mail), com o registro de sua decisão, para que possa consultá-la sempre que necessário. Caso queira, enviaremos uma cópia física do documento pelos correios.

Este estudo está sendo realizado para conhecer os efeitos pós-COVID-19 na cognição, ou seja, na memória e atenção, de adultos que tiveram COVID-19 durante a disseminação da variante Ômicron no Brasil. O estudo avaliará 126 participantes provenientes da pesquisa "Estudo Covid Longa Brasil", residentes em qualquer região do Brasil e que consentirem participar de forma voluntária.

Se você aceitar participar, os procedimentos do estudo envolvem a realização de uma avaliação mais detalhada da sua memória e atenção, realizada por um profissional habilitado para a aplicação dos testes específicos de cognição, por meio de videochamada. A videochamada será realizada em horário que for mais conveniente para você, considerando a recomendação de que você esteja em um local que se sinta confortável e seguro para

responder as perguntas da pesquisa e levará cerca de 45 minutos. Ao final da entrevista, você receberá por e-mail o resultado dos testes aplicados durante a entrevista com o significado e orientações referentes aos resultados. As videochamadas serão gravadas, para finalidade exclusiva de auditoria e revisão dos dados, estando o acesso limitado aos pesquisadores envolvidos.

Os riscos em participar do estudo são mínimos e relacionados ao desconforto em ter que responder algumas das perguntas e o tempo de duração da entrevista. Este estudo não tem benefícios diretos para você. Os benefícios são indiretos, ou seja, os resultados do estudo reverterão em conhecimentos que podem esclarecer o impacto da COVID-19 na população brasileira.

As informações relacionadas ao estudo poderão ser conhecidas por pessoas autorizadas como autoridades regulatórias ou sanitárias. Em razão dos procedimentos do estudo serem realizados por videochamada, será necessário manter um cadastro com seu nome completo e contatos, para garantir a correta identificação durante os procedimentos. Essas informações serão de acesso exclusivo aos pesquisadores que realizam os contatos telefônicos ou as videochamadas e ficarão registradas em formulários separados dos demais dados que você fornecerá. Os seus dados pessoais não serão compartilhados e não estarão acessíveis para a Merck do Brasil, financiadora deste estudo. Todas as suas informações serão tratadas de forma confidencial e sigilosa pelos pesquisadores do estudo.

Você não terá quaisquer despesas para participar desse estudo e você não receberá qualquer valor em dinheiro pela participação. Se ocorrer uma despesa por causa da sua participação (como por exemplos despesas de telefonia ou internet), o pesquisador responsável garante o ressarcimento dessa despesa. Em caso de danos comprovadamente decorrentes de sua participação no estudo, você terá direito a indenização.

Os resultados obtidos com este estudo são para fins científicos. Você terá a garantia de que quando os resultados forem publicados, não aparecerá seu nome e não será possível sua identificação em nenhuma forma. Quando os resultados forem publicados, você será avisado pela forma que for mais conveniente para você (telefone, e-mail ou carta) e você poderá acessar as informações na página eletrônica de divulgação do estudo (www.covidlonga.com.br).

Em caso de dúvidas sobre a pesquisa, você poderá entrar em contato com o pesquisador responsável Maicon Falavigna, (51) 9819-2778, no endereço Avenida General Flores da Cunha, 1050 sala 704, Cachoeirinha, Rio Grande do Sul, de segunda a sexta das 8:00 às 18:00 ou a qualquer momento pelo e-mail contato@covidlonga.com.br. Esta pesquisa foi analisada e aprovada pelo Comitê de Ética em Pesquisa (CEP) do Hospital Moinhos de Vento. O CEP é responsável pela avaliação e acompanhamento dos aspectos éticos de todas as pesquisas envolvendo seres humanos, visando garantir a dignidade, os direitos, a segurança e o bem-estar dos participantes de pesquisas. Caso você tenha dúvidas e/ou perguntas sobre seus direitos como participante desta pesquisa ou se estiver insatisfeito com a maneira como a pesquisa está sendo realizada, entre em contato com o Comitê de Ética em Pesquisa (CEP) do Hospital Moinhos de Vento, situado na Rua Ramiro Barcelos, 910, 4º andar, telefone 51-33143537, de segunda a sexta-feira, das 08:00 às 12:00 e das 13:00 às 18:00hs.

Consentimento do participante

A seguir, ao assinalar a opção "SIM, eu consinto participar do estudo", significa que você consente em participar como voluntário(a) deste estudo. Você confirma que foi devidamente informado(a) e esclarecido(a) sobre o objetivo deste estudo, que você leu ou que foi lido para você os procedimentos nela envolvidos, assim como os possíveis riscos e benefícios decorrentes da sua participação e que você sanou todas as suas dúvidas. Você confirma que foi garantido que você pode se recusar a participar e retirar o seu consentimento a qualquer momento, sem que isto cause qualquer prejuízo, penalidade ou responsabilidade a você ou sua família. Você autoriza a divulgação dos dados obtidos neste estudo mantendo em sigilo a sua identidade. Você confirma que o pesquisador que conduziu o consentimento para participar neste estudo garantiu que você receberá uma via deste documento com todas as páginas rubricadas e assinadas por você e pelo pesquisador que aplicou este termo.

Se você **não quiser participar deste estudo**, você deve assinalar a opção "NÃO quero participar do estudo". Após isso, você não será mais contatado.

Você consente participar do estudo?

( ) SIM, consinto participar de forma voluntária do estudo.

( ) NÃO quero participar do estudo.

Se potencial participante selecionou "NÃO quero participar do estudo" aparecerá a seguinte mensagem:

Você decidiu NÃO participar do estudo.
Para confirmar essa decisão, por gentileza, clique em FINALIZAR.
Se deseja corrigir essa decisão, clique em VOLTAR.

Se potencial participante selecionou "FINALIZAR", o processo é encerrado e receberá por email a via do e-TCLE preenchida com essa decisão, contendo seu nome, data e hora que registrou a decisão, bem como o nome de quem disponibilizou e aplicou o e-TCLE.

Você consentiu participar de forma voluntária do estudo.
Para confirmar essa decisão, por gentileza, clique em FINALIZAR.
Se deseja corrigir essa decisão, clique em VOLTAR.

Ao selecionar "FINALIZAR" o processo de consentimento é concluído e o participante receberá por email a via do e-TCLE preenchida com essa decisão, contendo seu nome, data e hora que registrou a decisão, bem como o nome de quem disponibilizou e aplicou o e-TCLE.

Termo de Consentimento Livre e Esclarecido v.2 de 31 de julho de 2023.
Nome do participante: gerado automaticamente pelo sistema.
Data e hora do registro do consentimento pelo participante: gerado automaticamente pelo sistema.

Declaração do(a) pesquisador(a). Declaro que obtive de forma apropriada e voluntária o Consentimento Livre e Esclarecido deste participante para a participação neste estudo. Declaro ainda que me comprometo a cumprir o que está descrito neste termo.
Nome do pesquisador que disponibilizou e aplicou o e-TCLE: preenchido automaticamente pelo sistema.

>> OBS: as descrições em itálico são scripts sobre o processo do e-TCLE, não aparecem na via final do termo.
