# Supplementary material for: Incidence, associated factors and impact of the post-COVID-19 condition in Brazil: Study protocol of an observational cohort during the Omicron phase
Source: PLoS One. 2025 Apr 24;20(4):e0322466. doi: 10.1371/journal.pone.0322466 (PMC12021252; doi:10.1371/journal.pone.0322466)
Supplement: S2 File — (PDF) [file pone.0322466.s002.pdf]

SPIRIT

STANDARD PROTOCOL ITEMS: RECOMMENDATIONS FOR INTERVENTIONAL TRIALS

SPIRIT 2013 Checklist: Recommended items to address in a clinical trial protocol and related documents*

Section/item	Item No	Description	Addressed onpage number
**Administrative information**			
Title	1	Descriptive title identifying the study design, population, interventions, and, if applicable, trial acronym	Page 1 (manuscript)<br>Page 1 (study protocol)
Trial registration	2a	Trial identifier and registry name. If not yet registered, name of intended registry	NA
	2b	All items from the World Health Organization Trial Registration Data Set	Page 5 (manuscript)
Protocol version	3	Date and version identifier	Page 5 (manuscript)<br>Pages 1,2 (study protocol)
Funding	4	Sources and types of financial, material, and other support	Page 3,29 (study protocol)
Roles and responsibilities	5a	Names, affiliations, and roles of protocol contributors	Page 1 (manuscript)<br>Page 3 (study protocol)
	5b	Name and contact information for the trial sponsor	Page 1 (manuscript)<br>Page 3 (study protocol)
	5c	Role of study sponsor and funders, if any, in study design; collection, management, analysis, and interpretation of data; writing of the report; and the decision to submit the report for publication, includingwhether they will have ultimate authority over any of these activities	Page 28 (study protocol)
	5d	Composition, roles, and responsibilities of the coordinating centre, steering committee, endpoint adjudication committee, data management team, and other individuals or groups overseeing the trial, ifapplicable (see Item 21a for data monitoring committee)	Page 28 (study protocol)

**Introduction**			
Background and rationale	6a	Description of research question and justification for undertaking the trial, including summary of relevant studies (published and unpublished) examining benefits and harms for each intervention	Pages 4,5 (manuscript)<br>Page 10-13 (study protocol)
	6b	Explanation for choice of comparators	NA
Objectives	7	Specific objectives or hypotheses	Page 5 (manuscript)<br>Pages 13,14 (study protocol
Trial design	8	Description of trial design including type of trial (eg, parallel group, crossover, factorial, single group),allocation ratio, and framework (eg, superiority, equivalence, noninferiority, exploratory)	Page 6 (manuscript)<br>Page 15 (study protocol
**Methods: Participants, interventions, and outcomes**			
Study setting	9	Description of study settings (eg, community clinic, academic hospital) and list of countries where data willbe collected. Reference to where list of study sites can be obtained	Pages 6,7 (manuscript)<br>Page 15,16 (study protocol)
Eligibility criteria	10	Inclusion and exclusion criteria for participants. If applicable, eligibility criteria for study centres andindividuals who will perform the interventions (eg, surgeons, psychotherapists)	Pages 7,8 (manuscript)<br>Pages 16,17 (study protocol)
Interventions	11a	Interventions for each group with sufficient detail to allow replication, including how and when they will be administered	NA - observational
	11b	Criteria for discontinuing or modifying allocated interventions for a given trial participant (eg, drug dosechange in response to harms, participant request, or improving/worsening disease)	NA - observational
	11c	Strategies to improve adherence to intervention protocols, and any procedures for monitoring adherence(eg, drug tablet return, laboratory tests)	NA - observational
	11d	Relevant concomitant care and interventions that are permitted or prohibited during the trial	NA - observational
Outcomes	12	Primary, secondary, and other outcomes, including the specific measurement variable (eg, systolic blood pressure), analysis metric (eg, change from baseline, final value, time to event), method of aggregation (eg,median, proportion), and time point for each outcome. Explanation of the clinical relevance of chosen efficacy and harm outcomes is strongly recommended	Pages 9-11 (manuscript)<br>Page 19,20 (study protocol)

Participant timeline	13	Time schedule of enrolment, interventions (including any run-ins and washouts), assessments, and visits forparticipants. A schematic diagram is highly recommended (see Figure)	Page 5 (manuscript)<br>Pages 21,22 (study protocol)
	14	Estimated number of participants needed to achieve study objectives and how it was determined, including clinical and statistical assumptions supporting any sample size calculations	Pages 13,14 (manuscript)<br>Pages 23,24 (study protocol)
	15	Strategies for achieving adequate participant enrolment to reach target sample size	Pages 12,13 (manuscript)<br>Pages 15,16 (study protocol)<br>Recruitment and dissemination plan
**Methods: Assignment of interventions (for controlled trials)**			
Allocation:			
Sequence generation	16a	Method of generating the allocation sequence (eg, computer-generated random numbers), and list of any factors for stratification. To reduce predictability of a random sequence, details of any planned restriction (eg, blocking) should be provided in a separate document that is unavailable to those who enrol participantsor assign interventions	NA
Allocation concealment mechanism	16b	Mechanism of implementing the allocation sequence (eg, central telephone; sequentially numbered, opaque, sealed envelopes), describing any steps to conceal the sequence until interventions are assigned	NA
Implementation	16c	Who will generate the allocation sequence, who will enrol participants, and who will assign participants tointerventions	NA
Blinding (masking)	17a	Who will be blinded after assignment to interventions (eg, trial participants, care providers, outcomeassessors, data analysts), and how	NA
	17b	If blinded, circumstances under which unblinding is permissible, and procedure for revealing a participant'sallocated intervention during the trial	NA
**Methods: Data collection, management, and analysis**			
Data collection methods	18a	Plans for assessment and collection of outcome, baseline, and other trial data, including any related processes to promote data quality (eg, duplicate measurements, training of assessors) and a description ofstudy instruments (eg, questionnaires, laboratory tests) along with their reliability and validity, if known. Reference to where data collection forms can be found, if not in the protocol	Pages 12,13 (manuscript)<br>Page 23 (study protocol)
	18b	Plans to promote participant retention and complete follow-up, including list of any outcome data to becollected for participants who discontin ue or deviate from intervention protocols	Recruitment and dissemination plan

Data management	19	Plans for data entry, coding, security, and storage, including any related processes to promote data quality (eg, double data entry; range checks for data values). Reference to where details of data managementprocedures can be found, if not in the protocol	Page 27 (study protocol)
Statistical methods	20a	Statistical methods for analysing primary and secondary outcomes. Reference to where other details of thestatistical analysis plan can be found, if not in the protocol	Pages 14,15 (manuscript)<br>Pages 24-25 (study protocol)
	20b	Methods for any additional analyses (eg, subgroup and adjusted analyses)	NA
	20c	Definition of analysis population relating to protocol non-adherence (eg, as randomised analysis), and anystatistical methods to handle missing data (eg, multiple imputation)	NA
**Methods: Monitoring**			
Data monitoring	21a	Composition of data monitoring committee (DMC); summary of its role and reporting structure; statement ofwhether it is independent from the sponsor and competing interests; and reference to where further details about its charter can be found, if not in the protocol. Alternatively, an explanation of why a DMC is not needed	NA
	21b	Description of any interim analyses and stopping guidelines, including who will have access to these interimresults and make the final decision to terminate the trial	NA
Harms	22	Plans for collecting, assessing, reporting, and managing solicited and spontaneously reported adverseevents and other unintended effects of trial interventions or trial conduct	Page 16 (manuscript)<br>Page 26 (study protocol)
Auditing	23	Frequency and procedures for auditing trial conduct, if any, and whether the process will be independentfrom investigators and the sponsor	Page 13 (manuscript)<br>Page 23 (study protocol)
**Ethics and dissemination**			
Research ethics approval	24	Plans for seeking research ethics committee/institutional review board (REC/IRB) approval	Page 15 (manuscript)<br>Page 25 (study protocol)
Protocol amendments	25	Plans for communicating important protocol modifications (eg, changes to eligibility criteria, outcomes,analyses) to relevant parties (eg, investigators, REC/IRBs, trial participants, trial registries, journals, regulators)	Page 15 (manuscript)<br>Page 25 (study protocol)

Consent or assent	26a	Who will obtain informed consent or assent from potential trial participants or authorised surrogates, and how (see Item 32)	Page 16 (manuscript)<br>Pages 25,26 (study protocol)<br>Recruitment and dissemination Plan
	26b	Additional consent provisions for collection and use of participant data and biological specimens in ancillarystudies, if applicable	NA
Confidentiality	27	How personal information about potential and enrolled participants will be collected, shared, and maintainedin order to protect confidentiality before, during, and after the trial	Page 16 (manuscript)<br>Page 27 (study protocol)
Declaration of interests	28	Financial and other competing interests for principal investigators for the overall trial and each study site	COI
Access to data	29	Statement of who will have access to the final trial dataset, and disclosure of contractual agreements thatlimit such access for investigators	Manuscript submission form<br>Page 30 (study protocol)
Ancillary and post-trial care	30	Provisions, if any, for ancillary and post-trial care, and for compensation to those who suffer harm from trial participation	ICFs
Dissemination policy	31a	Plans for investigators and sponsor to communicate trial results to participants, healthcare professionals,the public, and other relevant groups (eg, via publication, reporting in results databases, or other data sharing arrangements), including any publication restrictions	Pages 16,17 (manuscript)<br>Page 30 (study protocol)<br>Recruitment and dissemination Plan
	31b	Authorship eligibility guidelines and any intended use of professional writers	Page 17 (manuscript)<br>Page 30 (study protocol)
	31c	Plans, if any, for granting public access to the full protocol, participant-level dataset, and statistical code	Page 30 (study protocol)
**Appendices**			
Informed consent materials	32	Model consent form and other related documentation given to participants and authorised surrogates	ICFs in the Supporting information File
Biological specimens	33	Plans for collection, laboratory evaluation, and storage of biological specimens for genetic or molecularanalysis in the current trial and for future use in ancillary studies, if applicable	NA

*It is strongly recommended that this checklist be read in conjunction with the SPIRIT 2013 Explanation & Elaboration for important clarification on the items.Amendments to the protocol should be tracked and dated. The SPIRIT checklist is copyrighted by the SPIRIT Group under the Creative Commons "Attribution-NonCommercial-NoDerivs 3.0 Unported" license.
